# Internal validation of an improved system for forensic application: a 41-plex Y-STR panel

**DOI:** 10.1093/fsr/owad012

**Published:** 2023-04-11

**Authors:** Siyu Chai, Min Li, Ruiyang Tao, Ruocheng Xia, Qianqian Kong, Yiling Qu, Liqin Chen, Shiquan Liu, Chengtao Li, Pengyu Chen, Suhua Zhang

**Affiliations:** Key Laboratory of Cell Engineering of Guizhou Province, Clinical Stem Cell Research Institute, Affiliated Hospital of Zunyi Medical University, Zunyi, China; Shanghai Key Laboratory of Forensic Medicine, Shanghai Forensic Service Platform, Academy of Forensic Sciences, Ministry of Justice, China, Shanghai, China; Shanghai Key Laboratory of Forensic Medicine, Shanghai Forensic Service Platform, Academy of Forensic Sciences, Ministry of Justice, China, Shanghai, China; School of Basic Medical Sciences, Shandong First Medical University & Shandong Academy of Medical Sciences, Jinan, China; Shanghai Key Laboratory of Forensic Medicine, Shanghai Forensic Service Platform, Academy of Forensic Sciences, Ministry of Justice, China, Shanghai, China; Shanghai Key Laboratory of Forensic Medicine, Shanghai Forensic Service Platform, Academy of Forensic Sciences, Ministry of Justice, China, Shanghai, China; Shanghai Key Laboratory of Forensic Medicine, Shanghai Forensic Service Platform, Academy of Forensic Sciences, Ministry of Justice, China, Shanghai, China; Department of Forensic Medicine, Inner Mongolia Medical University, Hohhot, China; Shanghai Key Laboratory of Forensic Medicine, Shanghai Forensic Service Platform, Academy of Forensic Sciences, Ministry of Justice, China, Shanghai, China; Department of Forensic Science, Medical School of Soochow University, Suzhou, China; Department of Forensic Medicine, Inner Mongolia Medical University, Hohhot, China; Institute of Evidence Law and Forensic Science, China University of Political Science and Law, Beijing, China; Shanghai Key Laboratory of Forensic Medicine, Shanghai Forensic Service Platform, Academy of Forensic Sciences, Ministry of Justice, China, Shanghai, China; Key Laboratory of Cell Engineering of Guizhou Province, Clinical Stem Cell Research Institute, Affiliated Hospital of Zunyi Medical University, Zunyi, China; Shanghai Key Laboratory of Forensic Medicine, Shanghai Forensic Service Platform, Academy of Forensic Sciences, Ministry of Justice, China, Shanghai, China; School of Basic Medical Sciences, Fudan University, Shanghai, China

**Keywords:** 41-plex Y-STR panel, validation, forensic genetics, male identification, familial searching

## Abstract

Y-chromosome short tandem repeats (Y-STRs) have a unique role in forensic investigation. However, low–medium mutating Y-STRs cannot meet the requirements for male lineage differentiation in inbred populations, whereas rapidly mutating (RM) high-resolution Y-STRs might cause unexpected exclusion of paternal lineages. Thus, combining Y-STRs with low and high mutation rates helps to distinguish male individuals and lineages in family screening and analysis of genetic relationships. In this study, a novel 6-dye, 41-plex Y-STR panel was developed and validated, which included 17 loci from the Yfiler kit, nine RM Y-STR loci, 15 low–medium mutating Y-STR loci, and three Y-InDels. Developmental validation was performed for this panel, including size precision testing, stutter analysis, species specificity analysis, male specificity testing, sensitivity testing, concordance evaluation, polymerase chain reaction inhibitors analysis, and DNA mixture examination. The results demonstrated that the novel 41-plex Y-STR panel, developed in-house, was time efficient, accurate, and reliable. It showed good adaptability to directly amplify a variety of case-type samples. Furthermore, adding multiple Y-STR loci significantly improved the system’s ability to distinguish related males, making it highly informative for forensic applications. In addition, the data obtained were compatible with the widely used Y-STR kits, facilitating the search and construction of population databases. Moreover, the addition of Y-Indels with short amplicons improves the analyses of degraded samples.

**Key Points:**

- A novel multiplex comprising 41 Y-STR and 3 Y-InDel was developed for forensic application.
- The multiplex included rapidly mutating Y-STRs and low–medium mutating Y-STRs, which is compatible with many commonly used Y-STR kits.
- The multiplex is a powerful tool for distinguishing related males, familial searching, and constructing DNA databases.

## Introduction

The non-recombination region (NRY) of the human Y chromosome is a highly informative haplotype system. Theoretically, male individuals in the same paternal lineage share identical NRY genetic information without considering the gradual accumulation of mutations [1]. Currently, genetic markers on the Y chromosome have been widely used in human genetics. Y-chromosomal short tandem repeats (Y-STRs) have proven to be excellent markers in forensic casework such as patrilineal relationship analysis in kinship testing and mixture deconvolution in sexual assault cases [2]. However, the discriminatory power of a limited number of Y-STRs is insufficient due to the lack of recombination on Y-chromosome, especially for male differentiation in an inbred population [3, 4]. Some different Y-STR systems with >20 Y-STRs have been developed [5–13], of which some rapidly mutating (RM) Y-STRs (mutation rates >1 × 10^−2^) could yield high-resolution paternal lineage differentiation. However, the main limitation of RM Y-STRs is that they may also lead to a false exclusion of male individuals from the same familial lineages [14]. In regard to low–medium mutating Y-STRs, they were shown to play a vital role in familial searching [6]. Therefore, we aimed at designing a novel panel using Y-STRs with both low and high mutation rates to better distinguish male individuals in pedigree screening and analysis of genetic relationships.

In this study, a novel 41-plex Y-STR panel was developed for co-amplifying 41 Y-STRs plus three Y-Indels in a 6-dye configuration. The system contains all the 17 Y-STRs from the Yfiler kit [15], and the inclusion of the nine RM Y-STRs improved the discrimination of related males [16]. Conversely, the 15 low–medium mutating Y-STRs made it suitable for familial searching. This novel panel was found to be compatible with PowerPlex Y23 [17] and the Yfiler Plus PCR Amplification kit [18], providing powerful haplotype recognition and data comparison compatibility. In addition, the amplicons of these three Y-InDels were designed to be very short, providing information even for highly degraded samples. Since their mutation rates are close to zero, the Y-InDels may also contribute to familial searching. Two internal quality controls, IPC60 and IPC500, were added to monitor polymerase chain reaction (PCR) efficiency and sample quality. To validate the efficiency of this 41-plex Y-STR panel, we tested size precision, stutter effect, species and male specificity, sensitivity, DNA mixture, concordance, PCR inhibition, and case-type samples, and performed a population study following the guidelines of the Scientific Working Group on DNA Analysis Methods [19].

## Materials and methods

### DNA samples

This study was approved by the Ethics Committee of the Academy of Forensic Science, Ministry of Justice, P. R. China. A total of 595 peripheral blood samples were obtained from Jilin Han males (*n* = 233) and Jilin Korean males (*n* = 362). Informed consent and declaration of genetic relationships with other participants were obtained from each recruited volunteer. Genomic DNA was extracted from the blood samples using the QIAamp DNA Blood Mini kit (Qiagen, Hilden, Germany), followed by quantification using the Qubit® dsDNA High Sensitivity Assay kit and a Qubit® 3.0 Fluorometer (Thermo Fisher Scientific, Waltham, MA, USA). Control DNA 9948 was purchased from Promega (Promega, Madison, WI, USA) and was used for sensitivity and inhibition studies. ddH_2_O was used as the negative control. For real casework samples, six different types of DNA samples: semen stains, saliva, buccal swabs, hair with follicles, nails, and muscle tissue were obtained from our laboratory (Academy of Forensic Sciences, Shanghai, China).

### Y-STRs selection

A total of 41 Y-STRs and three Y-InDels were selected, including 17 loci from the Yfiler kit [15] (DYS19, DYS385a/b, DYS389I, DYS389II, DYS390, DYS391, DYS392, DYS393, DYS437, DYS438, DYS439, DYS448, DYS456, DYS458, DYS635, and Y-GATA-H4), nine RM Y-STR loci [20] (DYS449, DYS518, DYS570, DYS576, DYS627, DYF387S1a/b, and DYF404S1a/b), 15 low-medium mutating loci [12, 20] (DYS549, DYS481, DYS533, DYS447, DYS527a/b, DYS460, DYS444, DYS643, DYS557, DYS596, DYS388, DYS522, DYS593, and DYS645), and rs199815934, rs759551978, and rs771783753. The system contains all loci from Yfiler [15], PowerPlex Y23 [17], and the Yfiler Plus PCR Amplification kit [18], compatible with the current worldwide mainstream Y-STR loci.

### Primer design

Primers were designed using the Primer Premier v5.0 and Oligo v6.0 software (Premier Biosoft International, Palo Alto, CA, USA) as previously described [21], with a primer length of 18–22 bp; optimum temperature of 50°C–60°C; optimum cytosine and guanine content of 40%C–60%; and amplicon length of 50–500 bp. Nonspecific hybridization of primers to other genome regions was filtered using the NCBI BLAST tool (https://blast.ncbi.nlm.nih.gov/Blast.cgi). Self-dimerization within the primers was checked using the AutoDimer v1.1 software (http://www.cstl.nist.gov/biotech/strbase/AutoDimerHomepage/AutoDimerProgramHomepage.htm). All loci were grouped in a single reaction according to the expected amplicon length and assigned to different dye-labeling fluorochromes at the 5′ end with either FAM (blue), HEX (green), TAMRA (yellow), ROX (red), or VIG (purple) (Applied Biosystems: Thermo Fisher Scientific).

### PCR amplification and electrophoresis

PCR amplification was performed by the GeneAmp 9700 PCR system (Thermo Fisher Scientific). To determine the optimal annealing temperature for PCR, 44 qualified primer pairs were amplified in triplicate at different annealing temperatures (58°C, 60°C, and 62°C) under the recommended 28 cycles with 1 ng of positive-control DNA 9948. At the optimized annealing temperature, three PCR cycle numbers (26, 28, and 30) were tested to determine the appropriate number of PCR cycles for the new panel.

The 3500XL Genetic Analyzer (Thermo Fisher Scientific) was used to analyze the PCR products at the default settings. The dye set of J6 Matrix Standards (Peoplespot Technology Ltd, Beijing, China) was used for spectral calibration. The T500 (orange) was used as the internal size standard. The fragments of T500 were 65, 70, 80 100, 120, 140, 160, 180, 200, 225, 250, 275, 300, 330, 360, 390, 420, 450, 490, and 500 bp. Capillary electrophoresis (CE) was performed by adding 1 μL of allelic ladder or amplified product and 0.5 μL of T500 to 8.5 μL deionized Hi-Di™ Formamide (Thermo Fisher Scientific). Then, the samples were denatured at 95°C for 3 min and chilled on ice before CE detection. Sample injection was performed in a POP 4 polymer (Thermo Fisher Scientific) using the operating conditions: injection at 3 kV for 10 s and electrophoresis at 15 kV for 1 500 s. GeneMapper® ID-X v1.2 (Thermo Fisher Scientific) was used to analyze the results with a 150 relative fluorescence unit (RFU) peak height threshold for genotyping.

**Figure 1:**
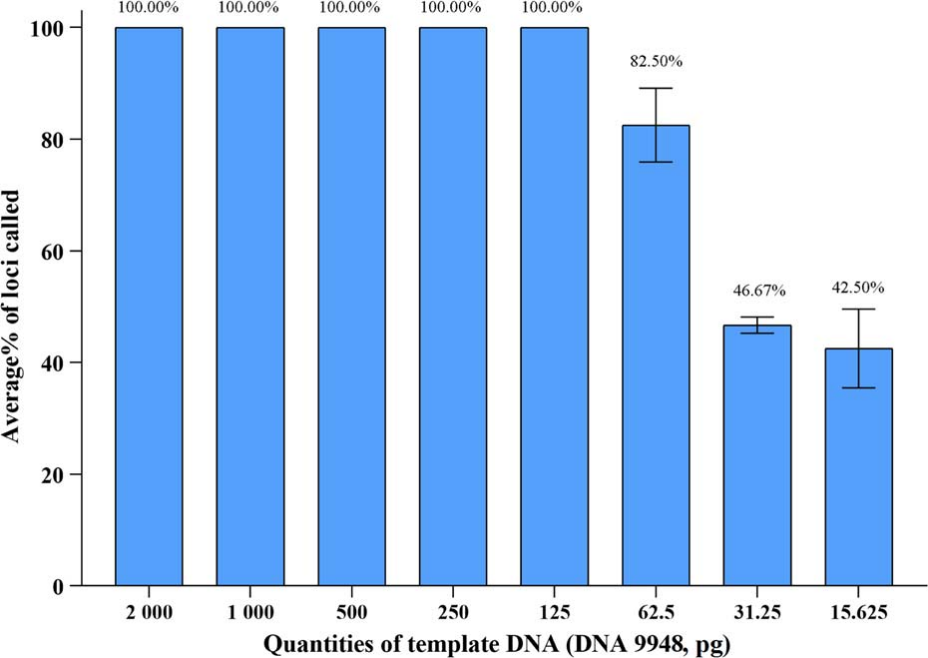
Electropherogram of the allelic ladder designed for the 41-plex Y-STR panel.

The allelic ladder was developed as previously described [22]. Briefly, the alleles reported in STRbase (https://strbase.nist.gov) and YHRD (http://www.yhrd.org/) and the data included in our previous works [21, 23–25] were used to determine locus ranges. At each locus, the PCR product of each allele was cloned into the T-vector and validated by Sanger sequencing. The PCR products of the cloned alleles were diluted, mixed, analyzed, and balanced to generate an allelic ladder for each locus, which were then mixed in appropriate proportions to form an allelic ladder for the panel [26] (Figure 1). All alleles presented in the ladder were validated by Sanger sequencing. The Panel and Bin files were programmed, and GeneMapper® ID-X v1.2 (Thermo Fisher Scientific) was used for genotyping. The nomenclature of the Y-STR alleles was defined following the latest recommendations of the DNA commission of the International Society of Forensic Genetics [27].

**Table 1:** General information of 41 Y-STRs and three Indels in the 41-plex Y-STR panel.

Locus	Mutation rate [20]	Dye	Repeat motif/alleles	Variation type	Allelic range	Haplotype of 9948
rs199815934	–	FAM	delTTCTC	InDel	1 or 2^a^	2
DYS456	4.94 × 10^−3^	FAM	AGAT	STR	13–18	17
DYS549	4.55 × 10^−3^	FAM	GATA	STR	7–17	13
DYS439	3.84 × 10^−3^	FAM	AGAT	STR	9–15	12
DYS19	4.37 × 10^−3^	FAM	TAGA	STR	10–20	14
DYS392	9.70 × 10^−4^	FAM	TAT	STR	7–20	13
DYS643	1.50 × 10^−3^	FAM	CTTTT	STR	6–17	11
DYS447	2.12 × 10^−3^	FAM	TAAWA	STR	21–28	25
DYS557	3.80 × 10^−3^	FAM	TTTC	STR	13–20	16
rs771783753	–	HEX	delCAT	InDel	1 or 2^a^	2
DYS391	3.23 × 10^−3^	HEX	TCTA	STR	5–16	10
DYS388	4.25 × 10^−4^	HEX	ATT	STR	9–16	12
DYS570	1.24 × 10^−2^	HEX	TTTC	STR	10–25	18
DYS635	3.85 × 10^−3^	HEX	TSTA	STR	17–27	23
DYS448	3.94 × 10^−4^	HEX	AGAGAT	STR	16–24	19
DYS437	1.53 × 10^−3^	HEX	TCTA	STR	13–16	15
DYS527	12.18 × 10^−3^ [12]	HEX	GGAA	STR	13–28	21,22
DYS444	5.45 × 10^−3^	HEX	TAGA	STR	10–16	12
rs759551978	–	TAMRA	delAGAT	InDel	1 or 2^a^	2
DYS393	2.11 × 10^−3^	TAMRA	AGAT	STR	8–16	13
DYS389I	5.51 × 10^−3^	TAMRA	[TCTG][TCTA]	STR	10–15	13
DYS390	1.52 × 10^−3^	TAMRA	[TCTA][TCTG]	STR	17–28	24
DYS389II	3.83 × 10^−3^	TAMRA	[TCTG][TCTA] [TCTG][TCTA]	STR	24–34	31
DYS438	9.56 × 10^−4^	TAMRA	TTTTC	STR	8–14	11
DYS576	1.43 × 10^−2^	TAMRA	GATA	STR	11–23	16
DYS645	4.07 × 10^−4^	TAMRA	GTTTT	STR	7–9	8
DYF404S1	1.25 × 10^−2^	TAMRA	[TTTC]N42[TTTC]	STR	12–17	12,14
DYS460	6.22 × 10^−3^	ROX	TCCT	STR	8–14	11
DYS458	8.36 × 10^−3^	ROX	GAAA	STR	13–23	18
DYS481	4.97 × 10^−3^	ROX	CTT	STR	16–32	24
DYS385	2.08 × 10^−3^	ROX	GAAA	STR	7–24	11,14
DYS449	1.22 × 10^−2^	ROX	TTTC	STR	22–42	30
DYS596	4.24 × 10^−4^	ROX	[GAA][GGAGAA]	STR	14–17	16
Y_GATA_H4	3.22 × 10^−3^	VIG	TAGA	STR	8–14	12
DYS533	5.01 × 10^−3^	VIG	ATCT	STR	7–16	12
DYS627	1.23 × 10^−2^	VIG	[AGAA]N16[AGAG] [AAAG]N81[AAGG]	STR	16–25	22
DYS518	1.84 × 10^−2^	VIG	[AAAG][GAAG][AAAG] [GGAG][AAAG]	STR	32–43	38
DYF387S1	1.59 × 10^−2^	VIG	[AAAG] [GTAG] [GAAG] [AAAG] [GAAG] [AAAG] [GAAG] [AAAG]	STR	33–41	35,38
DYS593	1.51 × 10^−3^	VIG	AAAAC/AAAAT	STR	15–18	15
DYS522	1.04 × 10^−3^	VIG	GATA	STR	9–14	10

a “1 or 2” of InDels means insertion or delection.

Y-STR: Y-chromosome short tandem repeat.

### Sizing precision and stutter study

Sizing precision was tested by calculating the average base pair sizes and standard deviation (SD) for each allele from 10 injections of the allelic ladder on the same CE platform. Stutter peaks are common artifacts observed during PCR process. Fifty male samples were analyzed on a 3500XL Genetic Analyzer to assess the effects of the stutter peaks. Peaks that differed from the true allele by one repeat motif (*n* ± 1 repeat units) were considered stutter peaks. Stutter values were calculated by dividing the height of the stutter peaks by that of the true alleles. In this work, the analytical threshold of the stutter peak height was set to 50 RFU. A stutter filter (average stutter value plus 3 SDs) was also configured.

### Sensitivity

To evaluate the sensitivity of the 41-plex Y-STR panel, we measured the allele call rate for control DNA 9948 with inputs of 2, 1, 0.5, 0.25 ng, 125, 62.5, 31.25, and 15.625 pg.

### Species specificity

The DNAs of nine common animals (cat, chicken, cow, dog, duck, long-tailed macaque, pig, rabbit, and sheep) were extracted from saliva or tissue samples. Standard PCR protocol with 1 ng of the animal template DNA was used to evaluate the species specificity of the new panel. All procedures were conducted in accordance with the United Kingdom Animal (Scientific Procedures) Act 1986 (https://www.gov.uk/guidance/research-and-testing-using-animals), and approved by the Ethics Committee at the Academy of Forensic Science, Ministry of Justice, P.R. China.

### Male specificity

Ten female samples were used to verify the male specificity with a DNA input of 1 ng. Besides, mixed DNA samples from females and males were used to assess male specificity by mixing 125 ng of female DNA with 125, 12.5, 1.25, or 0.125 ng of male DNA 9948.

### DNA mixtures

Two random samples, M1 and M2, were selected for the male–male mixture study. A total of 1 ng of the mixture in known ratios of 1:1, 1:3, 3:1, 1:9, 9:1, 1:19, and 19:1 was amplified. Each mixture was tested in triplicate to reduce accidental errors and ensure the accuracy of the results.

### Concordance evaluation

The Yfiler plus kit, Yfiler kit, PowerPlex Y23 kit, and the 41-plex Y-STR panel were used, respectively, to genotype 50 unrelated males. The genotyping results of the same loci were consistent.

### PCR inhibition study

Three common PCR inhibitors, hematin, humic acid, and nigrosine (Sigma-Aldrich, Darmstadt, Germany), were used to assess the performance of the 41-plex Y-STR panel. Stock solutions with high concentration were prepared by dissolving each of the inhibitors in 0.1 N NaOH (nigrosine and hematin) or DNA suspension buffer (humic acid), which were further diluted in ddH_2_O to obtain a working stock. The quantity of control DNA 9948 was constant at 1 ng with the inhibitors at the following concentrations: 20, 40, 60, 80, 100, and 150 ng/μL of humic acid; 100, 150, 200, 250, 300, 400, and 500 ng/μL of nigrosine; and 100, 200, 300, 500, and 750 μmol/L of hematin. The analysis was performed in triplicate for each condition.

### Casework sample testing

Using standard conditions for real casework samples, semen stains, saliva, buccal swabs, hair with follicles, nails, and tissue were also amplified with the 41-plex Y-STR panel.

### Population study

A total of 595 unrelated males from the Jilin Han and Jilin Korean populations were genotyped using the 41-plex Y-STR panel under standard conditions. Allele and haplotype frequencies were calculated by direct counting. The gene diversity (GD) was computed as GD = *n* (1−Σ*P_a_*^2^)/(*n* − 1) [28], where *n* represents the total number of samples, and *P_a_* represents the frequency of the *a-*th allele at the locus. Haplotype diversity (HD) was computed in the same way as GD, as HD = *n*(1 − Σ*P_i_*^2^)/(*n* − 1) [29], where *P_i_* represents the frequency of the *i-*th haplotype, and *n* represents the total sample number. The match probability (MP) was computed as the sum of the squared haplotype frequencies. Discrimination capacity (DC) was determined by DC = h/*n*, where h represents the total number of unique haplotypes.

## Results and discussion

### Construction of the 41-plex Y-STR panel

In this study, a total of 41 Y-STR loci were selected to construct a robust panel able to co-amplify 10 RM Y-STRs, 31 low–medium mutating Y-STRs, and three Y-InDels in a single PCR procedure. The detailed information on all the loci is presented in Table 1, and the primer information is presented in Supplementary Table S1.

After the construction and optimization, the 41-plex Y-STR panel was optimized in a 10-μL reaction volume, including 2.0 μL 5 × Master Mix, 2 μL 5 × Primer Mix, 1 μL template DNA, or a 1.2-mm punch of the blood card sample and ddH_2_O to obtain a final reaction volume of 10 μL. The PCR samples were amplified in MicroAmp® Optical 96-well reaction plates using the GeneAmp PCR system 9700 based on the following conditions: 95°C for 2 min; 28 cycles of 94°C for 5 s, 60°C for 90 s, and 62°C for 60 s; 60°C for 5 min, and followed by a final hold at 15°C. The profile of positive-control DNA 9948 is shown in Supplementary Figure S1.

### Sizing precision and stutter study

**Figure 2:**
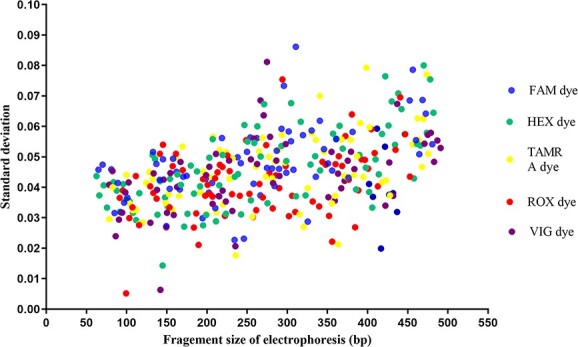
Sizing variation of the allelic ladders across 10 electrophoresis runs, analyzed on an AB 3500XL Genetic Analyzer.

Sizing precision was evaluated, and a targeted SD <0.1 was obtained for all alleles (Figure 2), indicating that the precision of the 41-plex Y-STR panel was better than those obtained in validation studies of other Y-STR kits [13, 15, 18] and good enough to distinguish microvariants or ladder peaks.

Stutters, caused by strand slippage, are the common by-products of PCR amplification [30–32]. To avoid complications in interpreting the profiles, the expected stutter ratio of each STR was accessed for the novel panel. We tested 50 male samples with 1 ng DNA inputs using the 41-plex Y-STR panel to obtain the ratio of stutter products. The average stutter ratio plus 3 SDs were applied to set a stutter file for GeneMapper® ID-X v1.2 (Table 2). A one repeat unit shorter or longer (*n* ± 1) than the true allele peak (*n*) was the dominant of two stutter products. The results showed that the repeated trinucleotide locus DYS481 had the highest *n*−1 stutter filter value (0.2468), whereas DYS385 had the highest *n* + 1 stutter filter value (0.2101).

**Table 2:** Stutter analysis of the 41-plex Y-STR panel per locus (*n* = 50).

Locus	*n* − 1 repeat units					*n* + 1 repeat units				
	Min	Max	Median	SD	Stutter filter	Min	Max	Median	SD	Stutter filter
DYF387S1	0.0645	0.1649	0.0942	0.0172	0.1458	0.0033	0.1271	0.0111	0.0367	0.1211
DYF404S1	0.0254	0.2009	0.0525	0.0252	0.1280	0.0031	0.0326	0.0079	0.0056	0.0247
DYS19	0.0454	0.1079	0.0663	0.0147	0.1105	0.0054	0.0375	0.0114	0.0064	0.0306
DYS385	0.0475	0.1443	0.0870	0.0280	0.1710	0.0032	0.4912	0.0098	0.0668	0.2101
DYS388	0.0503	0.1605	0.0863	0.0195	0.1449	0.0384	0.1241	0.0697	0.0172	0.1212
DYS389I	0.0369	0.2107	0.0520	0.0254	0.1283	0.0026	0.0242	0.0043	0.0046	0.0181
DYS389II	0.0694	0.2365	0.1210	0.0237	0.1922	0.0055	0.0386	0.0111	0.0071	0.0323
DYS390	0.0673	0.1168	0.0837	0.0122	0.1204	0.0023	0.0374	0.0045	0.0083	0.0295
DYS391	0.0453	0.1621	0.0713	0.0204	0.1326	0.0049	0.0214	0.0104	0.0049	0.0250
DYS392	0.0729	0.1571	0.1221	0.0203	0.1829	0.0329	0.1043	0.0727	0.0168	0.1232
DYS393	0.0588	0.1167	0.0748	0.0104	0.1061	0.0055	0.0461	0.0121	0.0066	0.0318
DYS437	0.0291	0.0671	0.0485	0.0124	0.0856	0.0025	0.0144	0.0062	0.0033	0.0160
DYS438	0.0155	0.0482	0.0212	0.0057	0.0382	0.0023	0.0043	0.0040	0.0011	0.0073
DYS439	0.0442	0.0944	0.0654	0.0096	0.0941	0.0083	0.0782	0.0239	0.0307	0.1160
DYS444	0.0395	0.1507	0.0616	0.0216	0.1263	0.0046	0.1319	0.0210	0.0372	0.1326
DYS447	0.0108	0.0552	0.0020	0.0089	0.0286	0.0035	0.0286	0.0107	0.0052	0.0264
DYS448	0.0132	0.0556	0.0254	0.0078	0.0487	0.0027	0.0204	0.0100	0.0069	0.0306
DYS449	0.0892	0.2007	0.1389	0.0199	0.1986	0.0065	0.0433	0.0149	0.0078	0.0383
DYS456	0.0863	0.1316	0.1056	0.0099	0.1353	–	–	–	–	–
DYS458	0.0602	0.1344	0.0953	0.0174	0.1475	0.0047	0.0595	0.0085	0.0140	0.0504
DYS460	0.0423	0.0879	0.0594	0.0105	0.0910	0.0056	0.0456	0.0105	0.0084	0.0358
DYS481	0.1355	0.2814	0.1791	0.0226	0.2468	0.0156	0.0447	0.0244	0.0061	0.0427
DYS518	0.0913	0.2182	0.1314	0.0211	0.1947	0.0065	0.0459	0.0131	0.0105	0.0445
DYS522	0.0333	0.0758	0.0556	0.0097	0.0848	0.0047	0.0124	0.0086	0.0024	0.0157
DYS527	0.0521	0.1507	0.0778	0.0255	0.1542	0.0034	0.1365	0.0182	0.0329	0.1170
DYS533	0.0504	0.1153	0.0751	0.0149	0.1197	0.0048	0.0533	0.0137	0.0132	0.0534
DYS549	0.0431	0.1007	0.0688	0.0119	0.1046	0.0103	0.0410	0.0155	0.0054	0.0318
DYS557	0.0703	0.1463	0.0855	0.0174	0.1378	0.0032	0.0234	0.0068	0.0042	0.0193
DYS570	0.0692	0.2995	0.0999	0.0318	0.1953	0.0058	0.0438	0.0128	0.0088	0.0391
DYS576	0.0658	0.1125	0.0837	0.0094	0.1120	0.0045	0.0375	0.0095	0.0071	0.0308
DYS593	0.0081	0.0473	0.0185	0.0089	0.0453	–	–	–	–	–
DYS596	0.0049	0.0177	0.0081	0.0028	0.0166	0.0021	0.0237	0.0124	0.0108	0.0448
DYS627	0.0368	0.1254	0.0871	0.0177	0.1401	0.0051	0.0449	0.0103	0.0078	0.0338
DYS635	0.0300	0.1219	0.0759	0.0192	0.1334	0.0036	0.0425	0.0105	0.0093	0.0384
DYS643	0.0143	0.0420	0.0213	0.0056	0.0382	0.0022	0.0187	0.0063	0.0061	0.0245
DYS645	0.0064	0.0170	0.0108	0.0022	0.0175	0.0087	0.0115	0.0090	0.0016	0.0137
Y_GATA_H4	0.0459	0.1150	0.0744	0.0132	0.1139	0.0078	0.0340	0.0114	0.0083	0.0362

Y-STR: Y-chromosome short tandem repeat.

### Sensitivity studies

**Figure 3:**
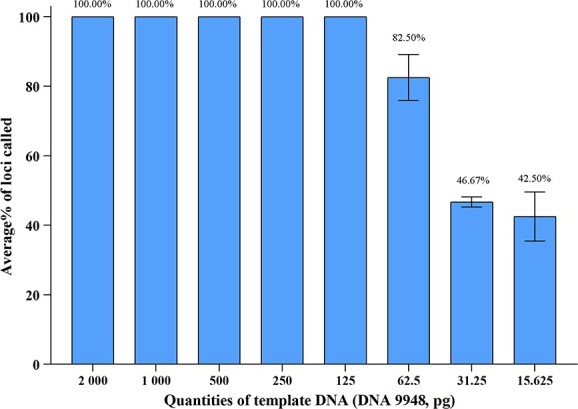
Sensitivity study of the 41-plex Y-STR panel with a template DNA ranging from 2 ng to 15.625 pg. Error bars represent the SD of triplicate experiments.

The sensitivity of the 41-plex Y-STR panel was assessed by serial dilution of control DNA 9948 from 15.625 pg to 2 ng in triplicate, resulting in complete Y-STR profiles obtained with DNA inputs ≥125 pg (Figure 3). Allele dropouts were observed when the DNA inputs decreased to 62.5 pg. The average call rates of 62.5, 31.25, and 15.625 pg input DNA were 82.5%, 46.67%, and 42.50%, respectively. Based on these observations, we concluded that the 41-plex Y-STR panel could generate reliable profiles with DNA inputs ≥125 pg (at a threshold of 150 RFU). Compared with other commonly used Y-STR kits [12, 15, 18], the 41-plex Y-STR panel demonstrated similar performance in terms of sensitivity.

### Species specificity

The 41-plex Y-STR panel was used to test nonhuman genomic DNA samples from cat, chicken, cow, dog, duck, long-tailed macaque, pig, rabbit, and sheep. The results showed no cross-reactions at any locus range above 150 RFU, except for the long-tailed macaque (Supplementary Figures S2 and S3). For this monkey species, an “OL” peak with a peak height of 1 705 RFU was found at DYS389II (Supplementary Figure S4), unlikely to disturb human genotyping. These results demonstrated the high human specificity of the 41-plex Y-STR panel, whereas samples mixed with primate DNA should be analyzed with caution.

### Male specificity

When processing mixed samples with DNA from both females and males, the specificity for the male component is critical for a given Y-STR system. The 41-plex Y-STR panel identified one to three reproducible artifacts marked as “OL” from 10 female samples (Supplementary Figure S5), which were out on the bin set and would not affect the correct genotyping profile. Besides, as shown in Supplementary Figures S6 and S7, although there was a general decrease in the peak heights, the 41-plex Y-STR panel could still detect male samples at an extreme mixing ratio of the males and females (1:1 000).

### DNA mixture examination

**Figure 4:**
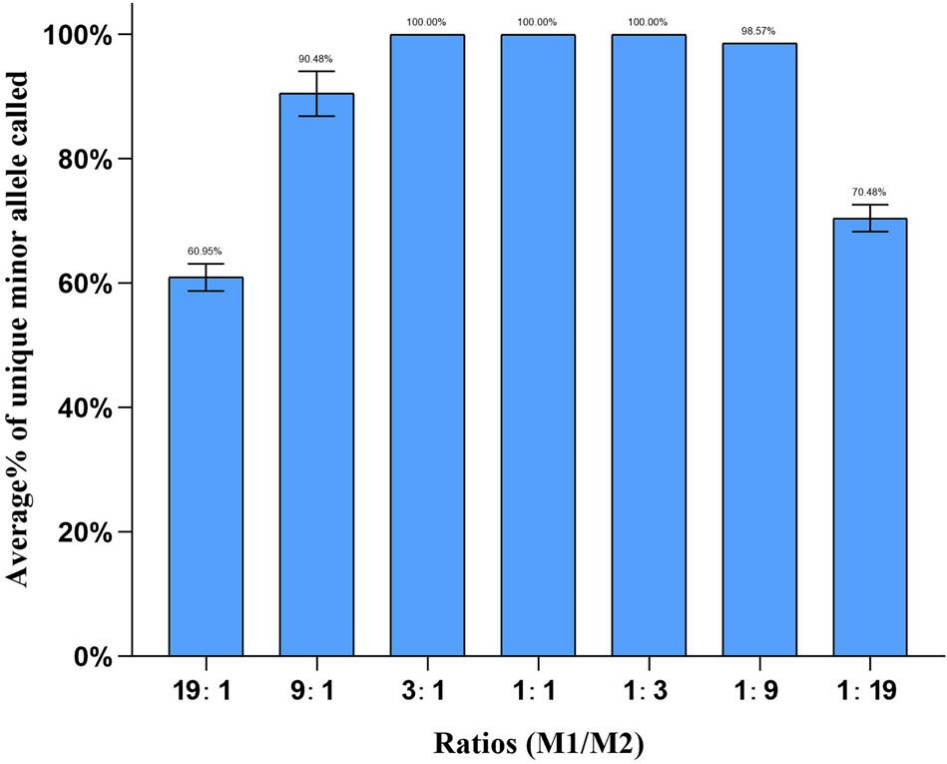
Mixture study of the 41-plex Y-STR panel detecting minor alleles from mixtures of two male samples (M1/M2). Error bars represent the SD of triplicate experiments.

DNA mixtures are usually encountered in daily forensic works. Here, two male donors were used to evaluate the performance of the 41-plex Y-STR panel on DNA mixtures. The genotypes of sample M1 and sample M2 are shown in Supplementary Table S2. They had only eight identical alleles, making them beneficial for mixture profile discrimination. The peak heights of minor alleles declined with an increase in mixing ratio. Complete genotypes of the minor donor at nonoverlapping and nonstutter positions were observed at the mixing ratios of 1:1, 1:3, and 3:1 (Figure 4). The profiles generated at the mixing ratios of 1:1, 1:3, and 3:1 are shown in Supplementary Figures S8–S10. Allele loss was found at ratios of 1:9, 9:1, 1:19, and 19:1, and the unique minor profile was observed at an average of 98.57% and 90.48%, 70.48% and 60.95%, respectively. These findings indicate that the 41-plex Y-STR panel allows all alleles to be correctly detected for 1:1, 1:3, or 3:1 male:male DNA mixtures.

### Inhibition study

**Figure 5:**
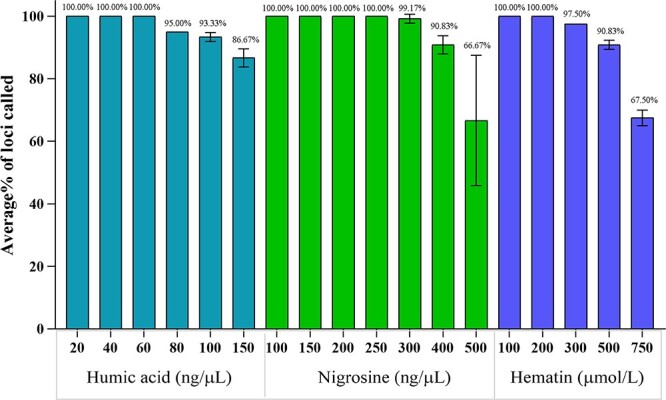
Effects of PCR inhibitors on the performance of the 41-plex Y-STR panel. The average percentage of loci detected in reactions containing 1 ng of control DNA 9948, with increasing concentrations of humic acid, nigrosine, and hematin.

Mock inhibition samples with 1 ng of male control DNA 9948 containing varying concentrations of humic acid, nigrosine, and hematin were prepared to evaluate the inhibitor tolerance of the 41-plex Y-STR panel. Complete profiles were observed with ≤60 ng/μL of humic acid, ≤250 ng/μL of nigrosine, and ≤ 200 μmol/L of hematin (Figure 5). When the concentration further increased, allelic dropouts were observed at the loci with longer fragment size, whereas shorter ones were preferentially detected. These results indicated that the 41-plex Y-STR panel had greater resistance to the PCR inhibitors than the 36-plex Y-STR system [7] and Yfiler® kit [15].

### Case-type sample testing

Apart from blood samples, various biological materials are encountered in routine forensic works. Here, the 41-plex Y-STR panel was applied to several nonhematological case-type samples to evaluate its ability in obtaining reliable genotypes from common case-type samples. The results showed that each sample yielded full genotypes, indicating that the 41-plex Y-STR panel was reliable and suitable for forensic application. The 41-plex Y-STR panel demonstrated good adaptability and could be used for direct amplification of various samples, such as blood cards, saliva cards, FTA cards, and cotton swabs (data not shown), and can also be used for amplification detection of extracted template DNA.

**Table 3:** Forensic parameters of the Yfiler, PowerPlex Y23, Yfiler plus, and 41-plex Y-STR panels.

Population	Maker set (number of Y-STRs)	Times haplotype observed		Distinct haplotypes	HD	DC	MP
		1	2				
Han (*n* = 233)	Yfiler (17)	225	4	229	0.999 852	0.982 833	0.000 018
	PowerPlex Y23 (23)	229	2	231	0.999 926	0.991 416	0.000 018
	Yfiler Plus (27)	231	1	232	0.999 963	0.995 708	0.000 074
	41Y (41)	233	0	233	1	1	0.004 292
Korean (*n* = 362)	Yfiler (17)	338	12	350	0.999 816	0.966 851	0.000 008
	PowerPlex Y23 (23)	352	5	357	0.999 923	0.986 188	0.000 008
	Yfiler Plus (27)	362	0	362	1	1	0.002 808
	41Y (41)	362	0	362	1	1	0.002 762
Total (*n* = 595)	Yfiler (17)	563	16	579	0.999 909	0.973 109	0.000 003
	PowerPlex Y23 (23)	581	7	589	0.999 960	0.988 235	0.000 003
	Yfiler Plus (27)	593	1	594	0.999 994	0.998 319	0.000 003
	41Y (41)	595	0	595	1	1	0.001 681

HD: haplotype diversity;

DC: discrimination capacity;

MP: match probability;

Y-STR: Y-chromosome short tandem repeat.

### Population study

A total of 595 unrelated males recruited from Chinese Jilin Han and Jilin Korean were detected at 41 Y-STRs and three Y-InDels, which generated 595 distinct haplotypes (Supplementary Table S3). We identified 20 copy number variations (CNVs) and 42 microvariants including 11 single-copy loci (DYS522, DYS570, DYS576, etc*.*) and three multicopy loci DYS444, DYS447, and DYS449. All samples with variants are co-listed in Supplementary Table S4. The allele frequencies and GDs of all 41 Y-STRs and three Indel markers were calculated (Supplementary Tables S5 and S6), and as shown in Supplementary Figure S11, the number of observed alleles for these 41 Y-STRs ranged from four (DYS391 and DYS437) to 18 (DYS527a/b), and the GD values were distributed from 0.1128 (DYS645) to 0.9732 (DYS385a/b). In addition, compared with the Yfiler® Plus kit, the GD values of the added Y-STR loci, such as DYS522, DYS444, DYS557, DYF404S1a/b1, DYS447, DYS527a/b, were all >0.6585, indicating that these loci had higher GD.

Moreover, all Y-STR markers were used to analyze the forensic parameters in the two populations. According to the different panels of the Yfiler, PowerPlex Y23, and Yfiler Plus amplification systems (17, 23 and 27 Y-STR loci), we calculated the standard forensic parameters (HD, DC, and MP) for 233 Jilin Han samples and 362 Korean samples (Table 3). The 41-plex Y-STR panel showed that the number of observed unique haplotypes was higher than for the three commercial Y-STR kits and that this new panel provided improved HD and DC. Compared with Yfiler®, the 41-plex Y-STR panel demonstrated higher power of discrimination, with an increase in DC from 0.973 109 to 1.

## Conclusion

The 41-plex Y-STR panel developed in this study is a 6-dye multiplex that combines 41 Y-STRs and three Y-Indels, and could be at least complementary to or even substitute the commonly used Yfiler® kit, PowerPlex® Y23 System, and Yfiler® Plus kit. Compared with commonly used Y-STR systems, the co-amplification of 41 Y-STRs and three Y-InDel markers not only enhanced the system effectiveness but also had advantages such as high HD, DC, and data compatibility. In addition, a series of validation studies of the 41-plex Y-STR panel indicated that the panel possessed stable sizing precision, male and species specificity, and high sensitivity. Overall, these results demonstrate the robustness and validity of the 41-plex Y-STR panel for forensic sampling and indicate that it could be a convenient and reliable tool for distinguishing related males, familial searching, and constructing DNA databases.

## Authors’ contributions

Siyu Chai and Min Li developed the concept of the research and wrote the original draft of the manuscript. Ruiyang Tao, Ruocheng Xia, Qianqian Kong, Yiling Qu, Liqin Chen, Shiquan Liu, and Chengtao Li collected data and performed the analysis. Pengyu Chen and Suhua Zhang supervised and secured funds for the research. All authors contributed to manuscript revision and approved the final version.

## Compliance with ethical standards

All procedures performed in studies involving human participants were in accordance with the ethical standards of the institutional and/or national research committee and with the 1964 Declaration of Helsinki and its later amendments or comparable ethical standards. All applicable international, national, and/or institutional guidelines for the care and use of animals were followed. The study was approved by the Ethics Committee at the Academy of Forensic Science, Ministry of Justice, P.R. China. All participants were adequately informed and signed informed consent before sample collection. Chengtao Li initial holds the position of Editorial Board Member for *Forensic Sciences Research* and is blinded from reviewing or making decisions for the manuscript.

## Disclosure statement

None declared.

## Funding

This study was supported by grants from the National Youth Top-notch Talent of Ten Thousand Program (WRQB2019), the Shanghai Science and Technology Innovation Fund (21DZ2202700), National Natural Science Foundation of China (81930056), and Open Project of Ministry of Justice of China (KF202010). The funders had no role in study design, data analysis, publishing decisions, or manuscript preparation.

## Supplementary Material

Supplementary_Figures_owad012

Supplementary_Tables_owad012
