# Supplementary material for: Internal validation of an improved system for forensic application: a 41-plex Y-STR panel
Source: Forensic Sci Res. 2023 Apr 11;8(1):70–8. doi: 10.1093/fsr/owad012 (PMC10265952; doi:10.1093/fsr/owad012)
Supplement: Supplementary_Figures_owad012 [file supplementary_figures_owad012.docx]

**Internal Validation of an Improved System for Forensic Application: A 41-plex Y-STR Panel**

**Supplementary Figures**

**
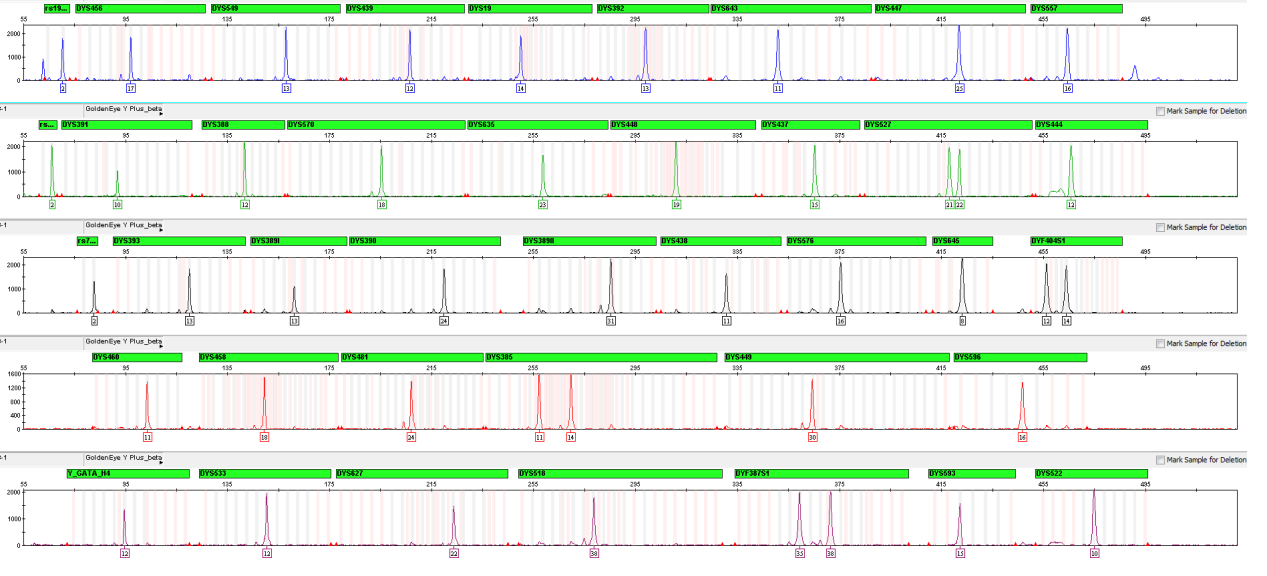
**

Figure S1. Genotyping profile of 9948 with 1 ng DNA amount.


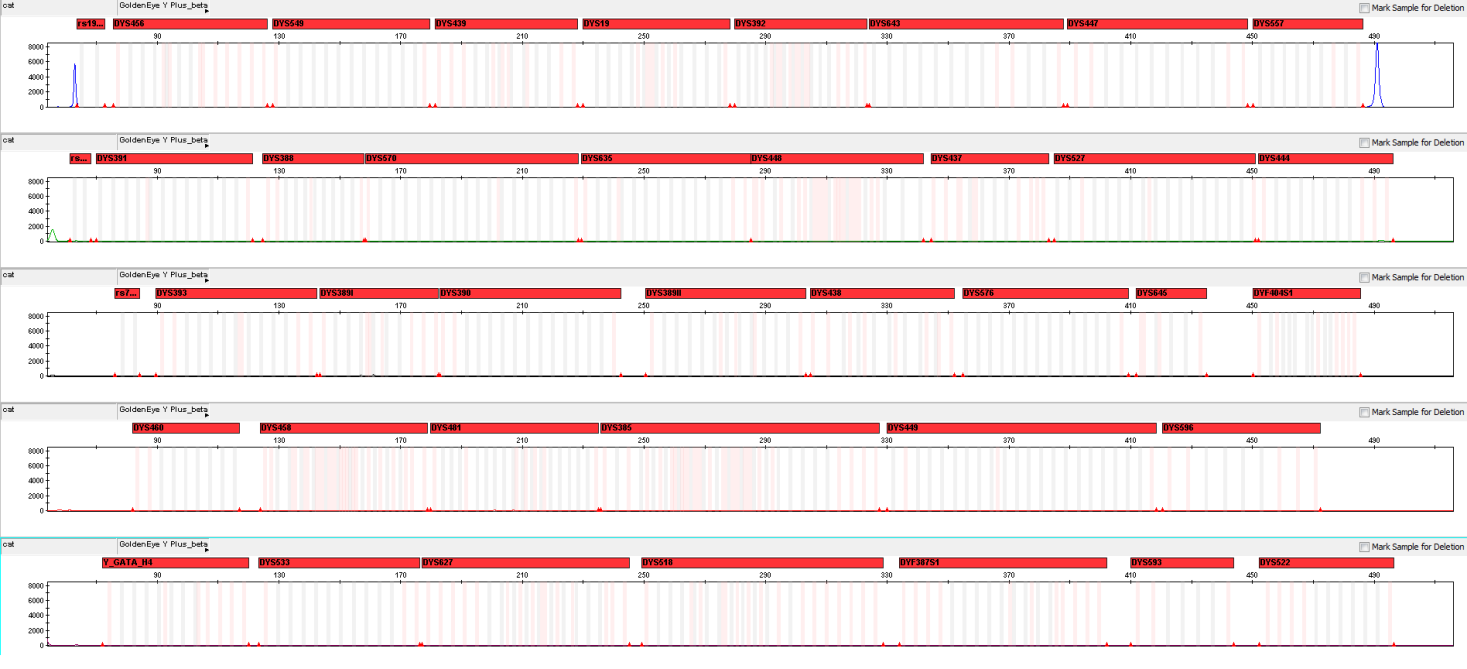


Figure S2. Electropherogram of 1 ng cat DNA with no reproducible peaks above 150 RFU.


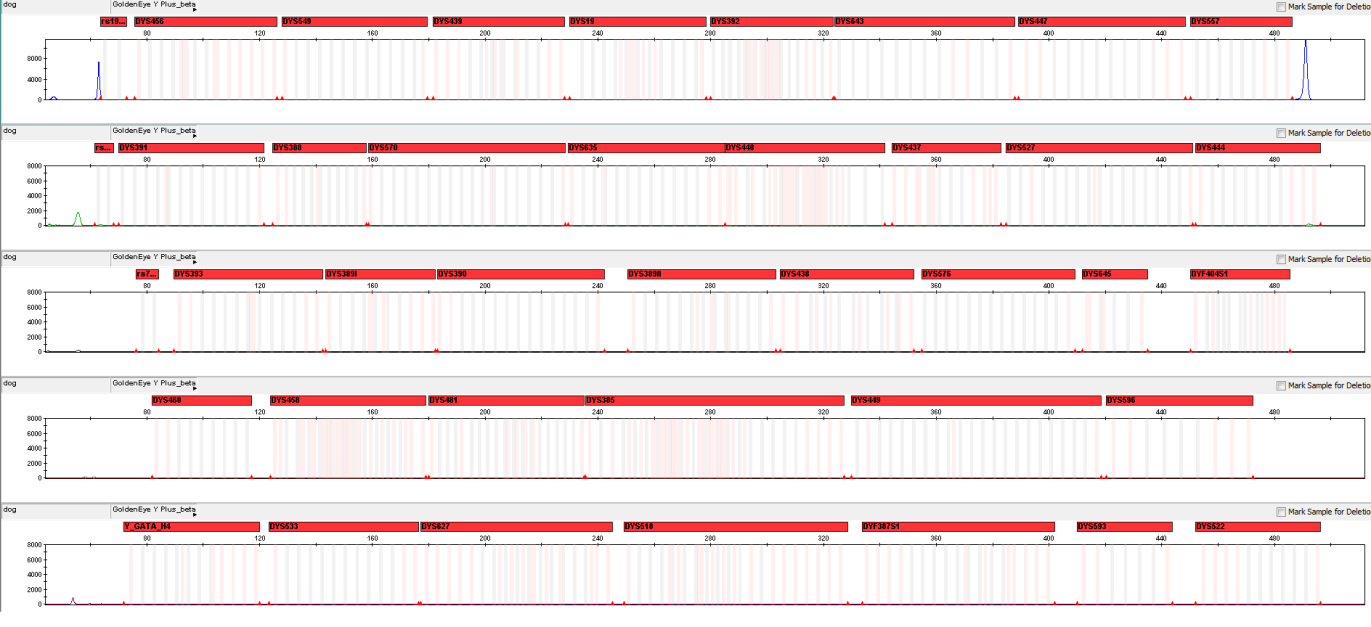


Figure S3. Electropherogram of 1 ng dog DNA with no reproducible peaks above 150 RFU.


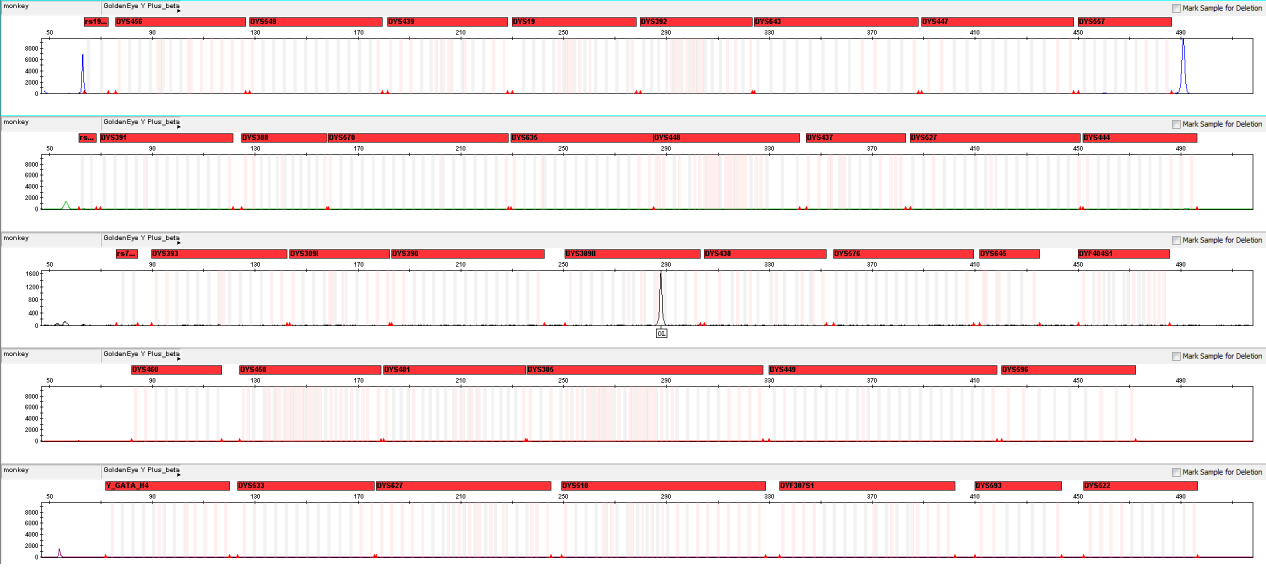


Figure S4. Electropherogram of 1 ng monkey DNA. One peak marked with "OL" was detected on DYS389II.


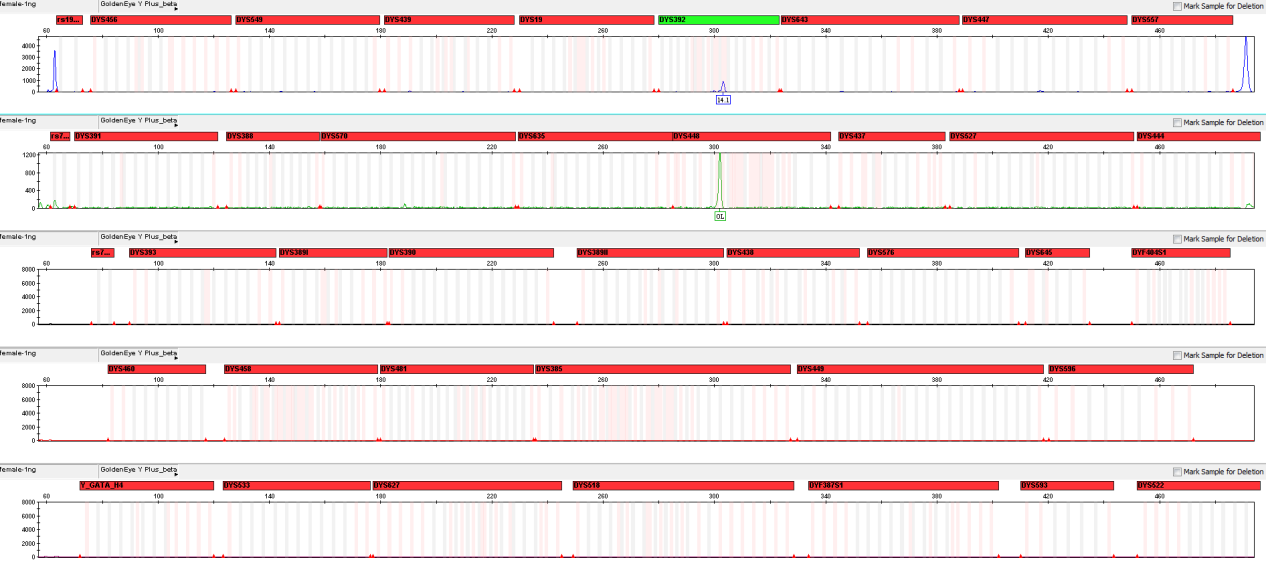


Figure S5. Genotyping profile of one female sample. One peak marked with allele 14.1 was detected on DYS392 and one off ladder (OL) peaks were detected on DYS448.


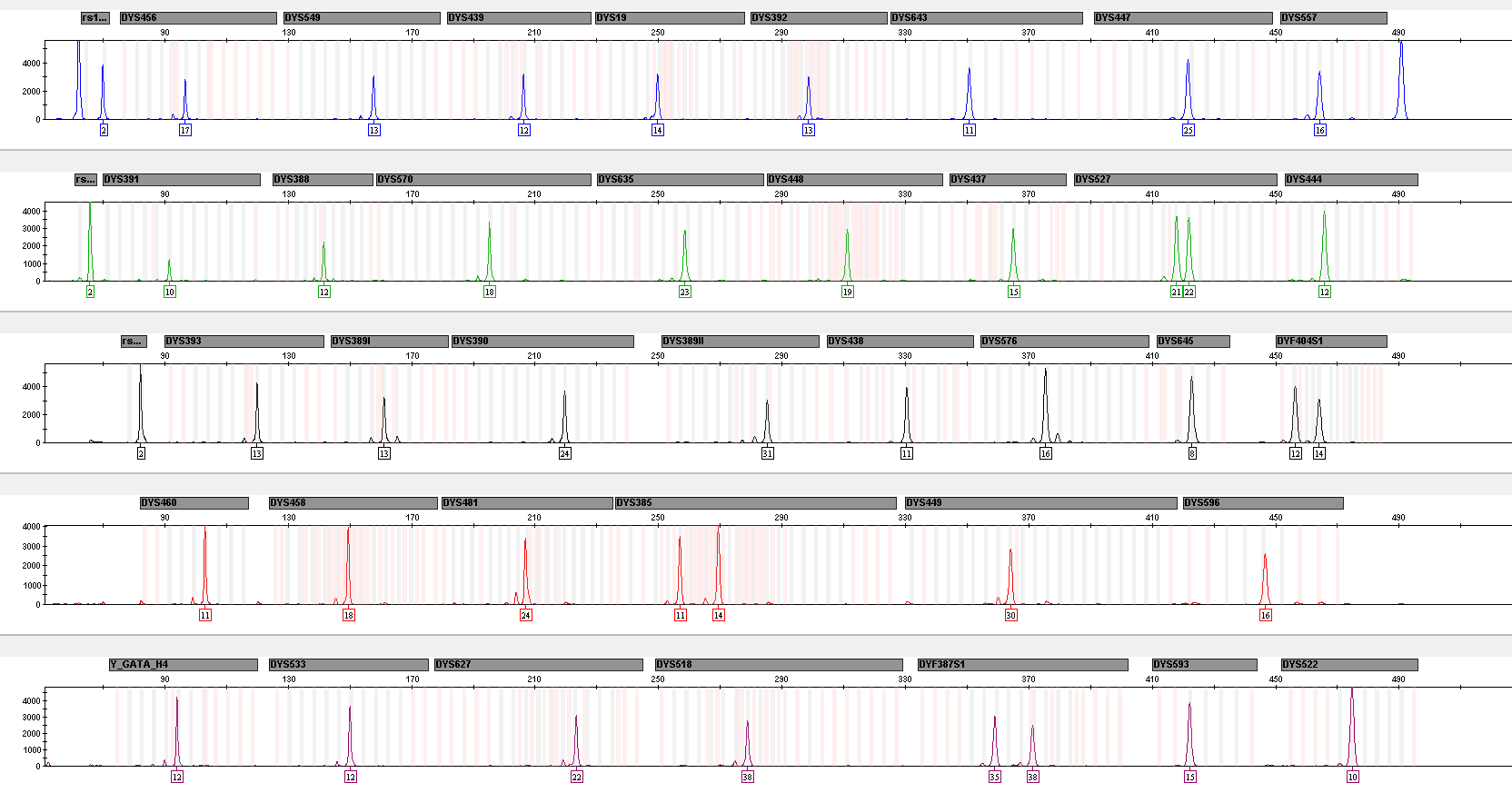


Figure S6. Genotyping profile of DNA 9948.


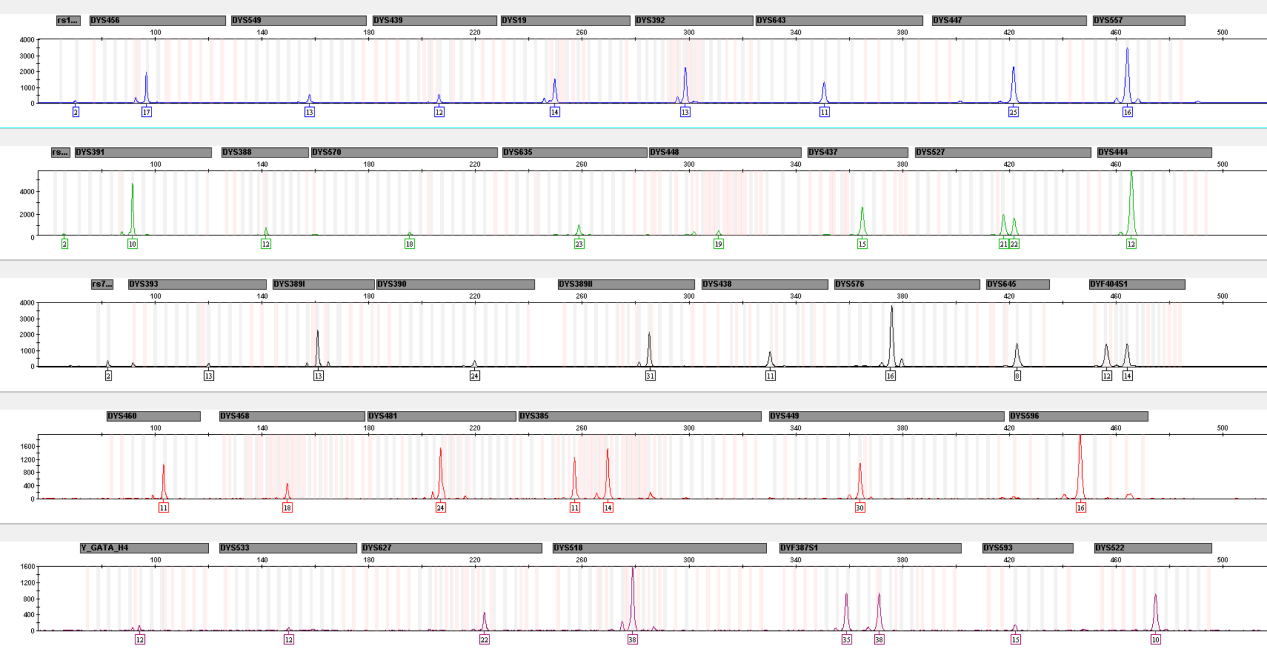


Figure S7. Genotyping profile of a 1:1000 male:female DNA mixture.


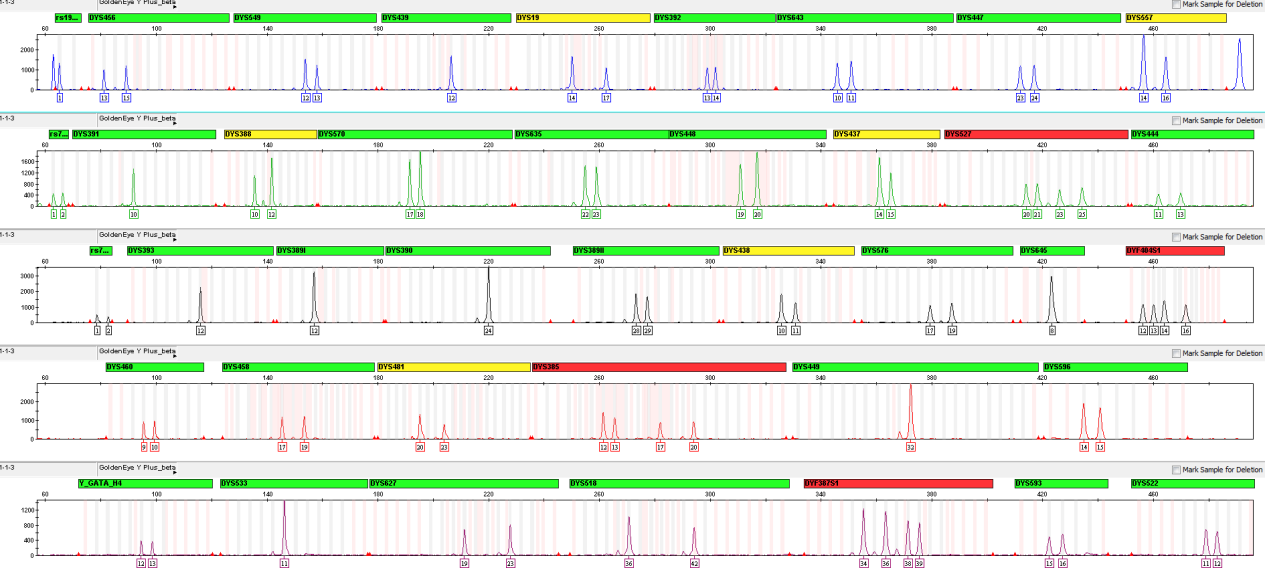


Figure S8. Genotyping profile of a 1:1 DNA mixture from two males.


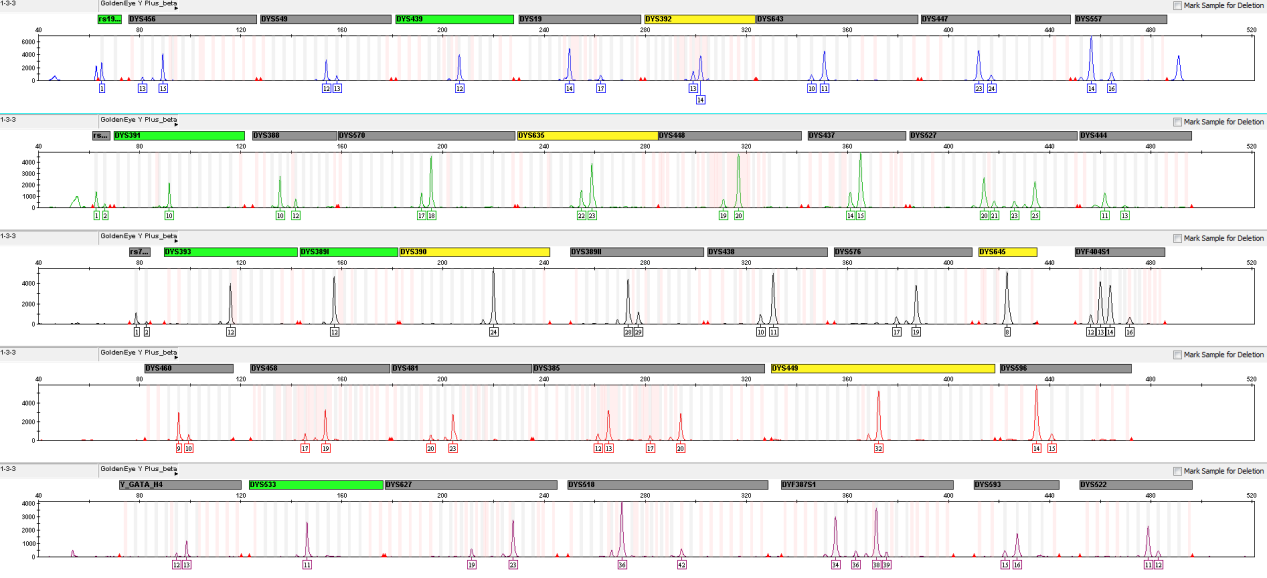


Figure S9. Genotyping profile of a 1:3 DNA mixture from two males.


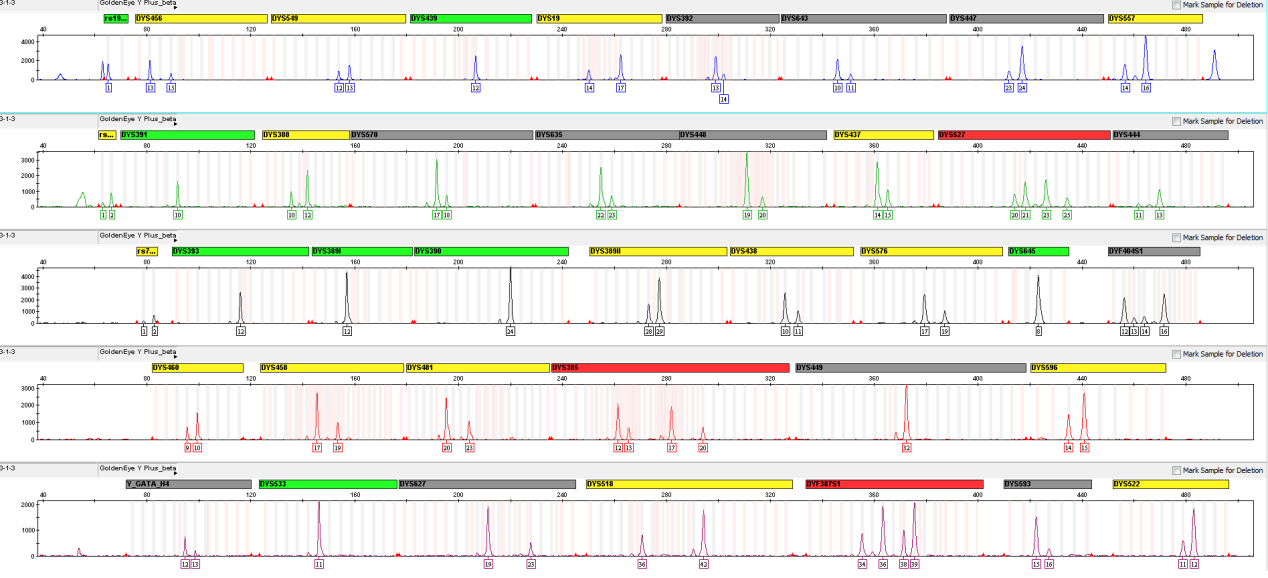


Figure S10. Genotyping profile of a 3:1 DNA mixture from two males.


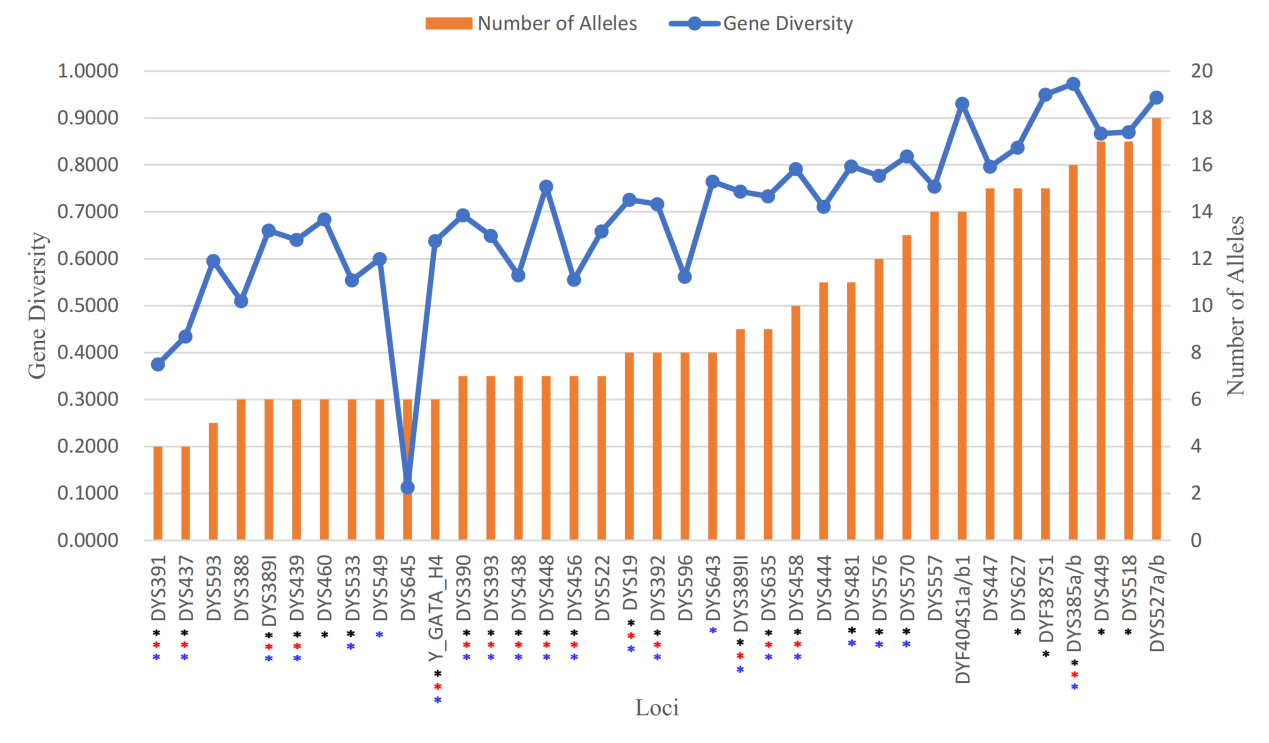


Figure S11. The number of alleles and gene diversities of 41 Y-STRs in the Jilin Han and Korean populations. Asterisks in black, red and blue represent the Y-STRs also included in the Yfiler^®^ Plus Kit, Yfiler^®^ Kit and PowerPlex^®^ Y23 System, respectively.
