# Supplementary material for: Internal validation of an improved system for forensic application: a 41-plex Y-STR panel
Source: Forensic Sci Res. 2023 Apr 11;8(1):70–8. doi: 10.1093/fsr/owad012 (PMC10265952; doi:10.1093/fsr/owad012)
Supplement: Supplementary_Tables_owad012 [file supplementary_tables_owad012.docx]

**TableS1. The primer information of the 41-plex Y-STR Panel.**

| loci | Forward primer | Reverse primer |
| --- | --- | --- |
| rs199815934 | TTTTCTACTGATACCTTTGTTTCTGT | GATTTAAACTCTCTGAATCAGGCACATG |
| DYS456 | GGGACCTTGTGATAATGTAAGAT | AGAGGGACAGAACTAATGGAATA |
| DYS549 | GCAATTAGGTAGGTAAAGAGGAAG | AAGGTTTTTTTTGGTGGCATAAGTG |
| DYS439 | TCGAGTTGTTATGGTTTTAGGT | TGGCTTGGAATTCTTTTACCCA |
| DYS19 | CACTATGACTACTGAGTTTCTGTTATAGTG | GCTGGTCAATCTCTGCACCTG |
| DYS392 | AGAGGGATCATTAAACCTACCAAT | GCAGTGGCCCAAGTGATCTT |
| DYS643 | AAATTTATGCATAGGAATGGTGGGT | ACACAATAAACTTTTGGGGACTT |
| DYS447 | CTTTGCGTTATCTCTGCCTT | GTTTCAGCCATAAAGTTAGCTCA |
| DYS557 | CTGAACCTACTGGCAGGTGT | TCTCTCCCGTGATGGAACAA |
| rs771783753 | CTTGTCACTAATTTTTGTTGAGTAATA | TACTAGTGACTGTTCTCAAGGTC |
| DYS391 | TTCAATCATACACCCATATCTG | ATAGAGGGATAGGTAGGCAGGCA |
| DYS388 | GAGGCGGAGCTTTTAGTGAGCC | CATGTGAGTTAGCCGTTTAGCGA |
| DYS570 | TGAAACGTAAAATGAATGATGACTA | TTCAGCATAGTCAAGAAACCAG |
| DYS635 | ATAGCAGCAAAATTCACAGTTG | CAAGAGTGTCTCACTTCAAGCAC |
| DYS448 | CGCGAGACAGAAAGGGAGATAGA | CCTTTTTTCTAGCCTCTTTCCTG |
| DYS437 | TATCTATGAATGATATTTATCTGTGGTCTA | CTGTCATTCACAGATGATATAGATAGATAG |
| DYS527ab | ggAAAAAATACTCACCAAAAATATAAACA | AGCACTTCAGCCCAGACAACA |
| DYS444 | ATCTGAAATATTTACATTTATGTATTTGTCAT | AAGAGGAGGCTTTGGTCTGGCT |
| rs759551978 | ATTAAGATAATTAAAATTGACAGTTATCAGT | TATAAAATAACTCACCAAAGGAATGC |
| DYS393 | TGTGGTCTTCTACTTGTGTCAAT | AACTCAAGTCCAAAAAATGAGGTAT |
| DYS389I | ACAATTATCCCTGAGTAGCAGAAGA | GTATCCAACTCTCATCTGTATTATCTATGTAT |
| DYS390 | TTATGAGTGGGAGAAATGGATGACAGTA | ATTTTTGGGCCCTGCATTTTGG |
| DYS389II | ACAATTATCCCTGAGTAGCAGAAGA | GTATCCAACTCTCATCTGTATTATCTATGTGT |
| DYS438 | GATTACCAAAATTAGTGGGGAATAGTTGAA | GGTGGATCACCCAGGGTCTGGA |
| DYS576 | TTTTTAATGTATGAGCAAGAATATCCTAG | GGATTATGGGAGCTAGAATTCAAGAT |
| DYS645 | GTGGCAATCATAGTCCATATAATTAACTTA | AACCACTGGTGACTGAATGCC |
| DYS404S1ab | AAAGATCAAAGGAGCCCAGGA | AAAAGATGATTTGCTTTATAATCAGTG |
| DYS460 | TCATCTATCCTCTGCCTATCATTTATTAT | GAGGAATCTGACACCTCTGACATA |
| DYS458 | AGCAACAGGAATGAAACTCCAATGA | AAAGTTCTGGCATTACAAGCATG |
| DYS481 | AAAGGAATGTGGCTAACGCTGT | ATTCATTGCAGATTCTTGGTCC |
| DYS385ab | CTATTCCAATTACATAGTCCTCCTTTCTT | GCATGGGTGACAGAGCTAGAC |
| DYS449 | CTAGAGATTCTTGGAGTCTCTCAA | GAAGTGGAGTTTGCTGTAAGCTA |
| DYS596 | CATTGATTTTTGTTTGTTTTCTAGTGAGAGA | TTCCAAGTGGTTCACAGAAATAAAGAATT |
| DYS443 | CAGGTCATGCCATACAGTGTAA | CTATTTCTGTGAACTACTCCTACTCAG |
| Y GATA H4 | ATCTATTCATCCATCTAATCTATCCATTCTA | AGATCTATAGATAGATAGGTAGGTAGGTAGA |
| DYS533 | TACCTATCATCTTTCTAGCTAGCTATCA | GTAAGTAGAGATCACCAATGAAATGTA |
| DYS627 | GGTGACAGCGCAGGATTCCA | TCCTTCTTACCTTCTTTACTTCCTCCCT |
| DYS518 | TGGGCAACACAAGTGAAACTGCTTCT | CACATCTTCAGCTCTTACCATGGGTGATT |
| DYF387S1ab | AGGGCCAAAGTTGCAACTGTTTT | GGTGACAGAGCTAGATTCCATTTTA |
| DYS593 | CAGGAAGCAGACCTTACATTGATAGA | GTTATCCTTTTTCTAGGTGGGAACTT |
| DYS522 | TGACCTCGAATCCTTTGAAATCATT | GCAGCAGTGTGACTTCTTAGGG |

**Table S2. Genotypes of sample M1,M2 for mixture study of 41-plex Y-STR Panel.**

| Sample | M1 | M2 |
| --- | --- | --- |
| DYF387S1 | 36,39 | 34,38 |
| DYF404S1 | 12,16 | 13,14 |
| DYS385 | 12,17 | 13,20 |
| DYS527 | 21,23 | 20,25 |
| DYS19 | 17 | 14 |
| DYS388 | 12 | 10 |
| DYS389I | 12 | 12 |
| DYS389II | 29 | 28 |
| DYS390 | 24 | 24 |
| DYS391 | 10 | 10 |
| DYS392 | 13 | 14 |
| DYS393 | 12 | 12 |
| DYS437 | 14 | 15 |
| DYS438 | 10 | 11 |
| DYS439 | 12 | 12 |
| DYS444 | 13 | 11 |
| DYS447 | 24 | 23 |
| DYS448 | 19 | 20 |
| DYS449 | 32 | 32 |
| DYS456 | 13 | 15 |
| DYS458 | 17 | 19 |
| DYS460 | 10 | 9 |
| DYS481 | 20 | 23 |
| DYS518 | 42 | 36 |
| DYS522 | 12 | 11 |
| DYS533 | 11 | 11 |
| DYS549 | 13 | 12 |
| DYS557 | 16 | 14 |
| DYS570 | 17 | 18 |
| DYS576 | 17 | 19 |
| DYS593 | 15 | 16 |
| DYS596 | 15 | 14 |
| DYS627 | 19 | 23 |
| DYS635 | 22 | 23 |
| DYS643 | 10 | 11 |
| DYS645 | 8 | 8 |
| Y_GATA_H4 | 12 | 13 |
| rs199815934 | 1 | 1 |
| rs759551978 | 2 | 1 |
| rs771783753 | 2 | 1 |

**Table S3. Genotyping results of the 595 unrelated Chinese male subjects by 41Y-STRs and 3 Indels.**

| Sample name | Population | DYF387S1 | DYF404S1 | DYS385 | DYS527 | DYS19 | DYS388 | DYS389I | DYS389II | DYS390 | DYS391 | DYS392 | DYS393 | DYS437 | DYS438 | DYS439 | DYS444 | DYS447 | DYS448 | DYS449 | DYS456 | DYS458 | DYS460 | DYS481 | DYS518 | DYS522 | DYS533 | DYS549 | DYS557 | DYS570 | DYS576 | DYS593 | DYS596 | DYS627 | DYS635 | DYS643 | DYS645 | Y_GATA_H4 | rs199815934 | rs759551978 | rs771783753 |
| --- | --- | --- | --- | --- | --- | --- | --- | --- | --- | --- | --- | --- | --- | --- | --- | --- | --- | --- | --- | --- | --- | --- | --- | --- | --- | --- | --- | --- | --- | --- | --- | --- | --- | --- | --- | --- | --- | --- | --- | --- | --- |
| S001 | Han | 36,40 | 15,16 | 16,17 | 21,22 | 14 | 12 | 13 | 28 | 25 | 11 | 13 | 12 | 15 | 11 | 12 | 12 | 24 | 20 | 32 | 15 | 18 | 10 | 23 | 35 | 11 | 10 | 12 | 14 | 19 | 21 | 15 | 15 | 23 | 21 | 12 | 8 | 11 | 1 | 2 | 2 |
| S002 | Han | 35,38 | 13,13 | 17,17 | 20,25 | 14 | 10 | 12 | 28 | 24 | 10 | 14 | 12 | 15 | 11 | 12 | 11 | 23 | 20 | 33 | 15 | 19 | 10 | 23 | 34 | 10 | 12 | 12 | 14 | 18 | 18 | 16 | 14 | 20 | 21 | 12 | 8 | 12 | 1 | 1 | 1 |
| S003 | Han | 35,38 | 13,14 | 13,17 | 20,22 | 15 | 10 | 12 | 27 | 25 | 10 | 14 | 12 | 16 | 11 | 12 | 13 | 23 | 20 | 34 | 15 | 17 | 9 | 24 | 35 | 12 | 12 | 13 | 13 | 18 | 20 | 16 | 14 | 20 | 21 | 10 | 8 | 12 | 1 | 1 | 1 |
| S004 | Han | 37,38 | 14,14 | 12,15 | 20,21 | 16 | 12 | 12 | 28 | 22 | 10 | 14 | 13 | 14 | 10 | 12 | 10 | 24 | 19 | 32 | 16 | 15 | 10 | 24 | 36 | 9 | 11 | 13 | 15 | 19 | 17 | 15 | 15 | 19.2 | 20 | 11 | 8 | 12 | 1 | 2 | 2 |
| S005 | Han | 37,39 | 13,13 | 12,18 | 19,20 | 16 | 13 | 13 | 29 | 24 | 11 | 11 | 14 | 14 | 10 | 10 | 13 | 27 | 21 | 32 | 15 | 17 | 10 | 25 | 37 | 11 | 12 | 12 | 17 | 16 | 16 | 17 | 15 | 22 | 20 | 9 | 8 | 12 | 2 | 2 | 2 |
| S006 | Han | 37,41 | 12,15 | 12,15 | 21,23 | 13 | 12 | 12 | 28 | 23 | 10 | 12 | 12 | 14 | 10 | 12 | 13 | 25 | 19 | 35 | 16 | 17 | 9 | 22 | 37 | 12 | 11 | 12 | 15 | 17 | 17 | 16 | 14 | 22 | 20 | 11 | 8 | 11 | 1 | 2 | 2 |
| S007 | Han | 36,36 | 14,14 | 11,12 | 21,21 | 14 | 13 | 14 | 31 | 23 | 10 | 14 | 13 | 14 | 11 | 11 | 12 | 25 | 19 | 28 | 16 | 16 | 10 | 22 | 38 | 13 | 11 | 12 | 14 | 18 | 20 | 15 | 14 | 20 | 21 | 10 | 8 | 12 | 2 | 2 | 2 |
| S008 | Han | 37,38 | 12,13 | 14,16 | 21,22 | 15 | 12 | 14 | 30 | 23 | 10 | 13 | 14 | 14 | 10 | 14 | 14 | 27 | 20 | 30 | 15 | 16 | 10 | 25 | 37 | 11 | 12 | 11 | 15 | 17 | 20 | 15 | 16 | 24 | 22 | 11 | 8 | 12 | 1 | 2 | 2 |
| S009 | Han | 36,36 | 14,14 | 13,18 | 23,24 | 15 | 12 | 14 | 30 | 24 | 11 | 13 | 14 | 14 | 10 | 11 | 13 | 27 | 18 | 27 | 15 | 19 | 10 | 23 | 43 | 13 | 10 | 12 | 14 | 18 | 19 | 15 | 15 | 20 | 21 | 10 | 8 | 10 | 1 | 2 | 2 |
| S010 | Han | 36,38,41 | 13,15,16 | 16,17 | 22,22 | 14 | 12 | 13 | 29 | 23 | 10 | 16 | 13 | 14 | 10 | 13 | 12 | 27 | 21 | 27 | 17 | 17 | 10 | 24 | 37 | 10 | 11 | 11 | 16 | 16 | 17 | 15 | 16 | 20 | 22 | 10 | 9 | 11 | 2 | 2 | 2 |
| S011 | Han | 34,38 | 12,13 | 13,18 | 21,22 | 14 | 10 | 12 | 28 | 24 | 10 | 14 | 12 | 15 | 11 | 12 | 13 | 24 | 20 | 33 | 16 | 17 | 10 | 24 | 37 | 11 | 11 | 13 | 14 | 19 | 17 | 16 | 14 | 18 | 21 | 11 | 8 | 12 | 1 | 1 | 1 |
| S012 | Han | 35,38 | 13,13 | 13,19 | 20,22 | 14 | 10 | 12 | 28 | 23 | 9 | 14 | 12 | 14 | 11 | 11 | 12 | 23 | 20 | 34 | 15 | 18 | 9 | 23 | 37 | 11 | 11 | 13 | 15 | 20 | 19 | 16 | 14 | 18 | 20 | 11 | 8 | 12 | 1 | 1 | 1 |
| S013 | Han | 34,39 | 13,13 | 13,19 | 20,25 | 14 | 10 | 12 | 27 | 23 | 11 | 14 | 12 | 15 | 11 | 12 | 13 | 23 | 20 | 34 | 16 | 19 | 9 | 27 | 35 | 11 | 12 | 12 | 14 | 17 | 19 | 16 | 14 | 19 | 20 | 10 | 8 | 11 | 1 | 1 | 1 |
| S014 | Han | 35,40 | 11,12 | 12,17 | 21,26 | 15 | 12 | 12 | 28 | 23 | 10 | 12 | 12 | 15 | 10 | 12 | 13 | 24 | 19 | 28 | 16 | 20 | 8 | 26 | 37 | 12 | 11 | 13 | 16 | 17 | 19 | 16 | 14 | 22 | 21 | 11 | 9 | 12 | 1 | 2 | 2 |
| S015 | Han | 37,37 | 12,15 | 10,17 | 19,22 | 16 | 13 | 13 | 31 | 23 | 10 | 11 | 14 | 14 | 10 | 11 | 12 | 29 | 21 | 29 | 15 | 16 | 11 | 24 | 38 | 11 | 11 | 12 | 16 | 16 | 16 | 17 | 15 | 22 | 21 | 9 | 8 | 11 | 2 | 2 | 2 |
| S016 | Han | 38,39 | 14,15 | 13,14 | 21,23 | 15 | 12 | 12 | 28 | 23 | 11 | 14 | 13 | 14 | 10 | 11 | 11 | 25 | 18 | 32 | 17 | 15 | 10 | 25 | 39 | 10 | 11 | 11 | 15 | 19 | 17 | 15 | 15 | 23 | 19 | 11 | 8 | 12 | 1 | 2 | 2 |
| S017 | Han | 38,38 | 15,15 | 11,13 | 21,21 | 14 | 12 | 14 | 30 | 23 | 11 | 17 | 14 | 14 | 11 | 10 | 14 | 25 | 19 | 28 | 14 | 15 | 11 | 20 | 38 | 12 | 11 | 12 | 14 | 19 | 16 | 15 | 14 | 22 | 21 | 11 | 8 | 12 | 2 | 2 | 2 |
| S018 | Han | 37,39 | 13,17 | 14,25 | 22,22 | 15 | 12 | 13 | 30 | 23 | 10 | 13 | 13 | 15 | 10 | 11 | 14 | 23 | 20 | 32 | 15 | 16 | 9 | 24 | 38 | 11 | 11 | 14 | 15 | 20 | 18 | 16 | 15 | 21 | 20 | 12 | 8 | 11 | 1 | 2 | 2 |
| S019 | Han | 35,35 | 13,15 | 13,16 | 19,24 | 14 | 12 | 12 | 28 | 23 | 10 | 12 | 12 | 15 | 8 | 12 | 13 | 24 | 17 | 34 | 15 | 18 | 9 | 23 | 39 | 12 | 12 | 13 | 15 | 16 | 17 | 16 | 14 | 22 | 20 | 11 | 8 | 11 | 1 | 2 | 2 |
| S020 | Han | 38,38 | 14,14 | 11,11 | 24,24 | 14 | 13 | 14 | 30 | 23 | 10 | 14 | 13 | 14 | 10 | 12 | 12 | 22 | 18 | 27 | 16 | 15 | 11 | 22 | 37 | 12 | 12 | 12 | 14 | 19 | 18 | 15 | 14 | 20 | 21 | 10 | 8 | 12 | 2 | 2 | 2 |
| S021 | Han | 37,39 | 16,17 | 13,21 | 21,24 | 15 | 12 | 13 | 28 | 24 | 11 | 14 | 12 | 15 | 10 | 14 | 13 | 25 | 19 | 32 | 15 | 18 | 9 | 29 | 40 | 13 | 11 | 12 | 14 | 18 | 16 | 17 | 15 | 21 | 21 | 11 | 8 | 11 | 1 | 2 | 2 |
| S022 | Han | 30,39 | 13,13 | 13,20 | 21,21 | 14 | 12 | 14 | 31 | 23 | 10 | 14 | 12 | 15 | 10 | 13 | 13 | 22 | 20 | 30 | 13 | 18 | 10 | 25 | 38 | 13 | 11 | 13 | 14 | 17 | 16 | 16 | 15 | 22 | 20 | 11 | 9 | 12 | 1 | 2 | 2 |
| S023 | Han | 37,40 | 15,16 | 14,18 | 21,22 | 13 | 12 | 14 | 29 | 26 | 11 | 13 | 12 | 15 | 10 | 12 | 14 | 25 | 20 | 30 | 15 | 16 | 10 | 23 | 34 | 11 | 10 | 12 | 15 | 21 | 21 | 15 | 15 | 23 | 21 | 12 | 8 | 11 | 1 | 2 | 2 |
| S024 | Han | 37,37 | 14,14 | 12,17 | 21,21 | 16 | 13 | 13 | 32 | 24 | 10 | 11 | 14 | 14 | 10 | 12 | 13 | 25 | 21 | 30 | 15 | 15 | 11 | 24 | 38 | 12 | 12 | 12 | 17 | 18 | 15 | 17 | 15 | 21 | 21 | 10 | 8 | 11 | 2 | 2 | 2 |
| S025 | Han | 38,39 | 15,15 | 13,13 | 21,23 | 15 | 12 | 12 | 29 | 23 | 11 | 14 | 13 | 14 | 10 | 11 | 11 | 25 | 18 | 31 | 16 | 15 | 10 | 25 | 38 | 10 | 11 | 12 | 16 | 18 | 17 | 15 | 15 | 24 | 19 | 11 | 8 | 13 | 1 | 2 | 2 |
| S026 | Han | 35,38 | 13,14 | 13,18 | 22,23 | 14 | 10 | 12 | 28 | 24 | 10 | 14 | 12 | 15 | 11 | 12 | 12 | 23 | 20 | 33 | 15 | 17 | 9 | 23 | 36 | 11 | 10 | 12 | 14 | 15 | 18 | 16 | 14 | 17 | 20 | 11 | 8 | 11 | 1 | 1 | 1 |
| S027 | Han | 36,37 | 14,14 | 13,19 | 21,22 | 15 | 10 | 12 | 28 | 24 | 10 | 14 | 12 | 14 | 11 | 12 | 12 | 23 | 20 | 36 | 15 | 16 | 12 | 23 | 34 | 11 | 11 | 13 | 13 | 18 | 18 | 16 | 14 | 18 | 21 | 11 | 8 | 13 | 1 | 1 | 1 |
| S028 | Han | 38,38 | 14,14 | 12,15 | 21,21 | 16 | 13 | 13 | 30 | 22 | 10 | 11 | 14 | 14 | 10 | 11 | 13 | 26 | 20 | 31 | 15 | 16 | 11 | 23 | 37 | 11 | 12 | 13 | 19 | 16 | 19 | 17 | 15 | 24 | 20 | 9 | 8 | 11 | 2 | 2 | 2 |
| S029 | Han | 36,36 | 15,15 | 12,13 | 25,25 | 15 | 12 | 13 | 29 | 21 | 11 | 14 | 15 | 14 | 10 | 11 | 14 | 28 | 19 | 32 | 16 | 18 | 12 | 24 | 40 | 13 | 10 | 12 | 15 | 17 | 21 | 15 | 15 | 21 | 23 | 12 | 8 | 11 | 1 | 2 | 2 |
| S030 | Han | 40,41 | 12,15 | 12,12 | 19,20 | 16 | 13 | 13 | 29 | 24 | 9 | 11 | 13 | 14 | 10 | 12 | 13 | 29 | 20 | 27 | 15 | 17 | 11 | 27 | 38 | 11 | 11 | 13 | 14 | 13 | 19 | 16 | 16 | 22 | 23 | 10 | 9 | 10 | 2 | 2 | 2 |
| S031 | Han | 35,39 | 15,15 | 10,19 | 20,21 | 15 | 12 | 14 | 30 | 23 | 10 | 13 | 13 | 15 | 13 | 12 | 14 | 25 | 19 | 30 | 15 | 18 | 12 | 22 | 40 | 12 | 11 | 11 | 14 | 17 | 17 | 16 | 15 | 20 | 21 | 12 | 8 | 11 | 1 | 2 | 2 |
| S032 | Han | 37,39 | 14,14 | 13,13 | 22,23 | 15 | 12 | 12 | 29 | 23 | 10 | 14 | 13 | 14 | 10 | 11 | 11 | 25 | 18 | 31 | 15 | 18 | 11 | 24 | 38 | 10 | 11 | 12 | 14 | 18 | 19 | 15 | 15 | 21 | 20 | 11 | 8 | 11 | 1 | 2 | 2 |
| S033 | Han | 35,38 | 13,13 | 13,17 | 19,23 | 15 | 10 | 12 | 28 | 24 | 10 | 14 | 12 | 15 | 11 | 13 | 14 | 24 | 21 | 32 | 14 | 19 | 9 | 23 | 36 | 11 | 11 | 12 | 14 | 19 | 18 | 16 | 14 | 14 | 20 | 11 | 8 | 12 | 1 | 1 | 1 |
| S034 | Han | 38,40 | 13,14 | 12,12 | 21,23 | 15 | 12 | 14 | 31 | 23 | 10 | 13 | 14 | 14 | 10 | 11 | 14 | 27 | 18 | 29 | 15 | 16 | 11 | 23 | 39 | 11 | 10 | 13 | 17 | 22 | 19 | 15 | 15 | 21 | 22 | 11 | 8 | 13 | 1 | 2 | 2 |
| S035 | Han | 36,38 | 14,16 | 12,19 | 19,21 | 16 | 12 | 14 | 29 | 23 | 11 | 11 | 14 | 14 | 10 | 12 | 12 | 26 | 21 | 27 | 16 | 16 | 9 | 24 | 36 | 11 | 12 | 12 | 18 | 16 | 17 | 17 | 15 | 24 | 22 | 9 | 8 | 11 | 2 | 2 | 2 |
| S036 | Han | 36,36 | 13,13 | 13,19 | 22,24 | 14 | 10 | 12 | 29 | 24 | 10 | 14 | 12 | 16 | 11 | 12 | 12 | 23 | 20 | 32 | 15 | 19 | 9 | 23 | 35 | 11 | 11 | 14 | 15 | 18 | 19 | 16 | 14 | 18 | 20 | 10 | 8 | 13 | 1 | 1 | 1 |
| S037 | Han | 35,38 | 13,15 | 13,20 | 20,24 | 14 | 10 | 12 | 28 | 23 | 10 | 15 | 12 | 14 | 11 | 11 | 11 | 23 | 20 | 35 | 15 | 17 | 9 | 23 | 37 | 11 | 11 | 13 | 14 | 19 | 18 | 16 | 14 | 18 | 20 | 11 | 8 | 13 | 1 | 1 | 1 |
| S038 | Han | 36,38 | 13,14 | 11,15 | 21,24 | 16 | 12 | 13 | 30 | 25 | 11 | 11 | 13 | 14 | 11 | 10 | 14 | 24 | 20 | 32 | 15 | 15 | 12 | 23 | 39 | 10 | 12 | 13 | 15 | 17 | 21 | 15 | 16 | 18 | 23 | 10 | 8 | 12 | 2 | 2 | 2 |
| S039 | Han | 35,38 | 13,13 | 13,20 | 20,23 | 14 | 10 | 12 | 28 | 23 | 10 | 14 | 12 | 14 | 11 | 12 | 12 | 23 | 20 | 36 | 15 | 17 | 9 | 23 | 37 | 11 | 11 | 13 | 14 | 19 | 18 | 16 | 14 | 18 | 20 | 11 | 8 | 13 | 1 | 1 | 1 |
| S040 | Han | 36,37 | 15,16 | 12,19 | 24,24 | 16 | 12 | 12 | 28 | 24 | 10 | 13 | 12 | 14 | 10 | 12 | 12 | 23 | 19 | 31 | 15 | 18 | 11 | 25 | 42 | 12 | 11 | 15 | 16 | 17 | 20 | 16 | 15 | 20 | 23 | 10 | 8 | 11 | 1 | 2 | 2 |
| S041 | Han | 35,37 | 13,13 | 13,19 | 20,23 | 14 | 10 | 13 | 29 | 24 | 10 | 14 | 12 | 15 | 11 | 12 | 13 | 23 | 20 | 34 | 15 | 14 | 9 | 23 | 39 | 10 | 11 | 12 | 14 | 19 | 18 | 18 | 14 | 19 | 20 | 13 | 8 | 13 | 1 | 1 | 1 |
| S042 | Han | 36,39 | 13,13 | 11,16 | 21,23 | 15 | 12 | 12 | 28 | 23 | 10 | 12 | 12 | 15 | 10 | 11 | 13 | 25 | 19 | 35 | 15 | 19 | 9 | 22 | 35 | 12 | 12 | 12 | 15 | 16 | 19 | 16 | 14 | 21 | 19 | 11 | 8 | 13 | 1 | 2 | 2 |
| S043 | Han | 36,38 | 13,13 | 14,18 | 24,25 | 14 | 10 | 12 | 28 | 24 | 10 | 15 | 12 | 15 | 10 | 12 | 12 | 23 | 20 | 29 | 15 | 18 | 9 | 23 | 35 | 11 | 10 | 12 | 14 | 18 | 20 | 16 | 14 | 19 | 21 | 11 | 8 | 12 | 1 | 1 | 1 |
| S044 | Han | 36,38 | 13,13 | 13,19 | 20,24 | 14 | 10 | 12 | 28 | 23 | 10 | 14 | 12 | 14 | 11 | 12 | 12 | 23 | 20 | 34 | 15 | 17 | 9 | 23 | 36 | 10 | 11 | 13 | 14 | 20 | 18 | 16 | 14 | 19 | 20 | 11 | 8 | 11 | 1 | 1 | 1 |
| S045 | Han | 35,39 | 14,14 | 12,18 | 20,22 | 15 | 12 | 12 | 28 | 22 | 10 | 12 | 12 | 15 | 10 | 13 | 13 | 24 | 19 | 33 | 15 | 18 | 9 | 22 | 36 | 12 | 11 | 14 | 15 | 17 | 19 | 16 | 14 | 21 | 20 | 11 | 8 | 12 | 1 | 2 | 2 |
| S046 | Han | 37,38 | 14,15 | 12,20 | 23,23 | 17 | 12 | 12 | 29 | 25 | 10 | 13 | 12 | 14 | 10 | 12 | 11 | 24 | 19 | 33 | 14 | 19 | 11 | 25 | 39 | 12 | 11 | 12 | 16 | 18 | 18 | 17 | 15 | 22 | 21 | 10 | 8 | 12 | 1 | 2 | 2 |
| S047 | Han | 37,39 | 16,16 | 12,18 | 19,21 | 16 | 12 | 14 | 30 | 23 | 10 | 11 | 14 | 14 | 10 | 12 | 13 | 26 | 21 | 30 | 16 | 17 | 10 | 24 | 35 | 11 | 12 | 12 | 18 | 17 | 17 | 17 | 15 | 24 | 21 | 9 | 8 | 11 | 2 | 2 | 2 |
| S048 | Han | 34,37 | 12,15 | 16,16 | 23,24 | 13 | 12 | 13 | 30 | 24 | 10 | 14 | 13 | 13 | 11 | 13 | 12 | 29 | 19 | 30 | 15 | 16 | 11 | 26 | 37 | 12 | 12 | 12 | 17 | 18 | 19 | 15 | 16 | 22 | 23 | 11 | 8 | 11 | 2 | 2 | 2 |
| S049 | Han | 36,38 | 15,18 | 12,19 | 23,24 | 15 | 12 | 12 | 28 | 24 | 10 | 13 | 12 | 14 | 10 | 12 | 13 | 24 | 19 | 31 | 14 | 16 | 11 | 25 | 43 | 12 | 11 | 12 | 16 | 18 | 19 | 16 | 15 | 21 | 22 | 10 | 8 | 11 | 1 | 2 | 2 |
| S050 | Han | 37,38 | 14,17 | 14,18 | 21,23 | 17 | 12 | 13 | 28 | 25 | 10 | 14 | 12 | 14 | 10 | 11 | 12 | 24 | 20 | 27 | 13 | 21 | 10 | 26 | 40 | 13 | 11 | 11 | 16 | 16 | 20 | 16 | 15 | 21 | 22 | 10 | 8 | 12 | 1 | 2 | 2 |
| S051 | Han | 37,37 | 13,13 | 13,21 | 21,22 | 15 | 12 | 12 | 29 | 25 | 11 | 13 | 12 | 15 | 10 | 11 | 13 | 23 | 20 | 30 | 14 | 16 | 10 | 24 | 41 | 12 | 11 | 12 | 14 | 19 | 16 | 17 | 15 | 22 | 21 | 11 | 8 | 13 | 1 | 2 | 2 |
| S052 | Han | 34.2,39 | 16,16 | 12,12 | 21,24 | 15 | 12 | 12 | 28 | 23 | 10 | 12 | 13 | 15 | 11 | 12 | 14 | 23 | 20 | 34 | 15 | 19 | 10 | 22 | 39 | 12 | 11 | 12 | 15 | 19 | 18 | 16 | 14 | 24 | 21 | 11 | 8 | 13 | 1 | 2 | 2 |
| S053 | Han | 37,37 | 16,16 | 12,13 | 22,22 | 14 | 12 | 14 | 30 | 23 | 10 | 15 | 13 | 14 | 10 | 11 | 12 | 26 | 19 | 29 | 15 | 16 | 11 | 23 | 36 | 12 | 12 | 13 | 14 | 19 | 19 | 15 | 14 | 21 | 21 | 12 | 8 | 12 | 2 | 2 | 2 |
| S054 | Han | 35,37 | 14,14 | 13,19 | 20,23 | 14 | 10 | 12 | 27 | 24 | 10 | 14 | 12 | 15 | 11 | 12 | 13 | 23 | 20 | 32 | 15 | 18 | 9 | 23 | 36 | 11 | 12 | 12 | 14 | 18 | 18 | 16 | 14 | 17 | 20 | 11 | 8 | 11 | 1 | 1 | 1 |
| S055 | Han | 38,38 | 14,14 | 12,13 | 23,23 | 14 | 12 | 13 | 29 | 23 | 10 | 15 | 13 | 14 | 10 | 14 | 12 | 29 | 19 | 27 | 15 | 16 | 11 | 22 | 35 | 12 | 12 | 12 | 14 | 17 | 18 | 15 | 14 | 21 | 21 | 12 | 8 | 12 | 2 | 2 | 2 |
| S056 | Han | 35,38 | 13,13 | 13,18 | 20,22 | 14 | 10 | 13 | 29 | 24 | 10 | 14 | 12 | 15 | 11 | 11 | 13 | 23 | 20 | 35 | 15 | 18 | 9 | 23 | 37 | 10 | 11 | 12 | 14 | 19 | 18 | 18 | 14 | 18 | 20 | 13 | 8 | 12 | 1 | 1 | 1 |
| S057 | Han | 39,39 | 15,15 | 15,22 | 22,22 | 13 | 12 | 14 | 30 | 24 | 9 | 14 | 14 | 14 | 12 | 10 | 13 | 27 | 19 | 30 | 15 | 17 | 10 | 26 | 38.2 | 10 | 12 | 13 | 20 | 19 | 18 | 15 | 16 | 22 | 22 | 10 | 8 | 10 | 2 | 2 | 2 |
| S058 | Han | 37,37 | 13,13 | 15,16 | 23,23 | 15 | 12 | 13 | 29 | 24 | 10 | 10 | 12 | 14 | 10 | 12 | 12 | 27 | 20 | 30 | 15 | 21 | 11 | 26 | 34 | 11 | 12 | 11 | 14 | 21 | 17 | 15 | 16 | 23 | 20 | 9 | 8 | 11 | 2 | 2 | 2 |
| S059 | Han | 36,38 | 15,15 | 11,19 | 19,21 | 17 | 13 | 13 | 29 | 23 | 11 | 11 | 14 | 14 | 10 | 11 | 12 | 27 | 21 | 29 | 15 | 17 | 12 | 25 | 36 | 11 | 12 | 13 | 17 | 15 | 17 | 17 | 15 | 18 | 21 | 9 | 8 | 11 | 2 | 2 | 2 |
| S060 | Han | 36,37 | 14,15 | 12,15 | 20,21 | 17 | 12 | 12 | 28 | 23 | 10 | 14 | 12 | 14 | 10 | 12 | 10 | 24 | 19 | 32 | 15 | 16 | 10 | 24 | 36 | 9 | 12 | 13 | 14 | 18 | 18 | 15 | 15 | 17.2 | 20 | 11 | 8 | 13 | 1 | 2 | 2 |
| S061 | Han | 36,38 | 13,16 | 14,18 | 23,23 | 16 | 12 | 12 | 28 | 25 | 10 | 13 | 12 | 14 | 10 | 11 | 12 | 24 | 20 | 27 | 15 | 17 | 11 | 24 | 40 | 12 | 11 | 14 | 17 | 20 | 16 | 16 | 15 | 20 | 23 | 10 | 8 | 13 | 1 | 2 | 2 |
| S062 | Han | 35,39 | 13,14 | 15,19 | 20,23 | 14 | 10 | 12 | 28 | 23 | 10 | 14 | 12 | 15 | 11 | 12 | 14 | 23 | 20 | 32 | 15 | 18 | 9 | 24 | 37 | 11 | 11 | 13 | 14 | 17 | 18 | 16 | 14 | 21 | 21 | 11 | 8 | 12 | 1 | 1 | 1 |
| S063 | Han | 37,37 | 15,16 | 13,16 | 19,20 | 16 | 12 | 12 | 27 | 23 | 10 | 11 | 13 | 14 | 10 | 12 | 13 | 26 | 21 | 29 | 15 | 17 | 10 | 24 | 38 | 12 | 11 | 14 | 17 | 17 | 17 | 17 | 15 | 20 | 21 | 9 | 8 | 11 | 2 | 2 | 2 |
| S064 | Han | 35,37 | 13,14 | 12,19 | 22,23 | 14 | 10 | 12 | 28 | 24 | 10 | 14 | 12 | 15 | 11 | 13 | 13 | 23 | 20 | 32 | 15 | 16 | 9 | 22 | 35 | 11 | 11 | 15 | 15 | 19 | 17 | 16 | 14 | 18 | 21 | 11 | 8 | 12 | 1 | 1 | 1 |
| S065 | Han | 36,39 | 12,17 | 14,18 | 23,23 | 16 | 12 | 12 | 29 | 25 | 11 | 13 | 12 | 14 | 10 | 11 | 13 | 24 | 20 | 27 | 14 | 16 | 10 | 24 | 37 | 12 | 10 | 13 | 17 | 16 | 17 | 16 | 15 | 20 | 23 | 10 | 8 | 12 | 1 | 2 | 2 |
| S066 | Han | 38,38 | 14,16 | 11,17 | 19,20 | 16 | 12 | 12 | 27 | 23 | 10 | 11 | 14 | 15 | 10 | 12 | 13 | 25 | 23 | 30 | 16 | 17 | 10 | 24 | 36 | 11 | 12 | 12 | 17 | 17 | 17 | 16 | 15 | 22 | 21 | 9 | 8 | 11 | 2 | 2 | 2 |
| S067 | Han | 42,42 | 16,16 | 12,16 | 22,22 | 15 | 12 | 13 | 29 | 23 | 10 | 12 | 12 | 15 | 10 | 12 | 12 | 24 | 20 | 31 | 15 | 14 | 9 | 25 | 38 | 12 | 12 | 12 | 16 | 17 | 19 | 16 | 14 | 24 | 20 | 12 | 8 | 12 | 1 | 2 | 2 |
| S068 | Han | 36,39 | 13,14 | 12,16 | 20,23 | 15 | 12 | 12 | 29 | 23 | 10 | 12 | 12 | 14 | 10 | 12 | 14 | 25 | 19 | 32 | 15 | 20 | 10 | 21 | 40 | 12 | 12 | 12 | 15 | 16 | 19 | 16 | 14 | 23 | 19 | 11 | 8 | 12 | 1 | 2 | 2 |
| S069 | Han | 36,36 | 13,13 | 11,12 | 23,23 | 14 | 13 | 14 | 32 | 23 | 10 | 14 | 13 | 14 | 10 | 11 | 12 | 27 | 19 | 29 | 15 | 14 | 9 | 22 | 37 | 11 | 11 | 13 | 14 | 18 | 18 | 15 | 14 | 23 | 23 | 10 | 8 | 12 | 2 | 2 | 2 |
| S070 | Han | 36,38 | 14,16 | 14,18 | 22,23 | 16 | 12 | 12 | 28 | 25 | 10 | 13 | 12 | 14 | 10 | 12 | 12 | 24 | 20 | 28 | 14 | 19 | 10 | 24 | 40 | 12 | 11 | 14 | 16 | 18 | 16 | 16 | 15 | 24 | 23 | 10 | 8 | 12 | 1 | 2 | 2 |
| S071 | Han | 36,39 | 12,13 | 13,19 | 23,24 | 13 | 10 | 12 | 30 | 23 | 10 | 15 | 12 | 14 | 11 | 12 | 12 | 24 | 20 | 33 | 14 | 16 | 9 | 23 | 36 | 13 | 11 | 12 | 14 | 18 | 20 | 16 | 14 | 17 | 21 | 11 | 8 | 12 | 1 | 1 | 1 |
| S072 | Han | 35,40 | 14,16 | 12,12 | 21,23 | 15 | 12 | 13 | 29 | 23 | 10 | 12 | 12 | 15 | 11 | 13 | 13 | 23 | 20 | 33 | 14 | 20 | 9 | 21 | 38 | 12 | 11 | 13 | 15 | 19 | 18 | 16 | 14 | 22 | 22 | 11 | 8 | 12 | 1 | 2 | 2 |
| S073 | Han | 37,38 | 16,16 | 14,18 | 23,23 | 14 | 12 | 12 | 28 | 25 | 10 | 13 | 12 | 14 | 10 | 11 | 12 | 25 | 20 | 27 | 15 | 17 | 10 | 24 | 38 | 12 | 11 | 13 | 15 | 17 | 16 | 16 | 15 | 21 | 22 | 10 | 8 | 12 | 1 | 2 | 2 |
| S074 | Han | 35,39 | 12,13 | 13,19 | 20,22 | 14 | 10 | 12 | 27 | 23 | 11 | 14 | 12 | 15 | 11 | 13 | 14 | 23 | 20 | 32 | 15 | 18 | 9 | 23 | 35 | 11 | 11 | 12 | 14 | 19 | 20 | 16 | 14 | 19 | 20 | 11 | 8 | 12 | 1 | 1 | 1 |
| S075 | Han | 35,38 | 16,16 | 13,18 | 23,23 | 16 | 12 | 14 | 29 | 23 | 10 | 12 | 13 | 14 | 10 | 13 | 12 | 21 | 19 | 29 | 15 | 18 | 10 | 25 | 38 | 12 | 12 | 14 | 15 | 18 | 16 | 15 | 15 | 18 | 24 | 11 | 8 | 12 | 1 | 2 | 2 |
| S076 | Han | 36,38 | 13,13 | 11,12 | 20,23 | 14 | 12 | 13 | 29 | 22 | 10 | 15 | 14 | 14 | 10 | 12 | 12 | 26 | 19 | 29 | 15 | 17 | 11 | 24 | 38 | 11 | 12 | 13 | 14 | 19 | 18 | 15 | 14 | 22 | 21 | 11 | 7 | 13 | 2 | 2 | 2 |
| S077 | Han | 35,39 | 15,16 | 12,13 | 19,25 | 15 | 12 | 10 | 27 | 23 | 11 | 12 | 12 | 15 | 10 | 12 | 13 | 24 | 19 | 33 | 15 | 18 | 9 | 22 | 38 | 14 | 11 | 13 | 15 | 17 | 18 | 16 | 14 | 19 | 20 | 11 | 8 | 12 | 1 | 2 | 2 |
| S078 | Han | 38,38 | 13,13 | 14,15 | 24,24 | 15 | 12 | 12 | 28 | 24 | 10 | 10 | 12 | 14 | 10 | 11 | 13 | 27 | 19 | 30 | 15 | 20 | 11 | 28 | 36 | 11 | 12 | 11 | 14 | 20 | 18 | 15 | 16 | 23 | 20 | 9 | 8 | 11 | 2 | 2 | 2 |
| S079 | Han | 39,40 | 11,14 | 14,17 | 20,23 | 15 | 12 | 12 | 27 | 24 | 10 | 13 | 13 | 15 | 10 | 12 | 12 | 24 | 19 | 30 | 16 | 17 | 10 | 24 | 35 | 12 | 12 | 12 | 15 | 15 | 19 | 16 | 16 | 23 | 21 | 12 | 8 | 12 | 1 | 2 | 2 |
| S080 | Han | 38,38 | 16,16 | 12,20 | 22,23 | 16 | 12 | 12 | 29 | 25 | 10 | 13 | 12 | 14 | 10 | 13 | 12 | 25 | 19 | 30 | 14 | 17 | 10 | 24 | 36 | 12 | 10 | 13 | 16 | 17 | 18 | 16 | 15 | 19 | 21 | 11 | 8 | 11 | 1 | 2 | 2 |
| S081 | Han | 37,37 | 13,13 | 11,11 | 23,23 | 14 | 13 | 13 | 28 | 23 | 10 | 14 | 13 | 14 | 11 | 12 | 12 | 25 | 19 | 26 | 16 | 17 | 11 | 22 | 37 | 12 | 12 | 12 | 13 | 18 | 17 | 15 | 14 | 20 | 21 | 10 | 8 | 12 | 2 | 2 | 2 |
| S082 | Han | 34,37 | 14,15 | 12,19 | 22,23 | 16 | 12 | 12 | 27 | 24 | 10 | 13 | 12 | 14 | 10 | 12 | 12 | 25 | 19 | 30 | 14 | 17 | 10 | 24 | 39 | 12 | 11 | 14 | 16 | 18 | 18 | 17 | 15 | 20 | 21 | 10 | 8 | 12 | 1 | 2 | 2 |
| S083 | Han | 36,38 | 15,16 | 12,21 | 23,23 | 16 | 12 | 12 | 27 | 25 | 11 | 13 | 12 | 14 | 10 | 12 | 12 | 24 | 19 | 30 | 16 | 19 | 10 | 24 | 37 | 12 | 11 | 12 | 16 | 17 | 18 | 17 | 15 | 20 | 22 | 10 | 8 | 12 | 1 | 2 | 2 |
| S084 | Han | 35,39 | 13,13 | 13,20 | 20,23 | 14 | 10 | 12 | 28 | 23 | 10 | 14 | 12 | 14 | 11 | 11 | 12 | 23 | 20 | 34 | 14 | 15 | 9 | 23 | 35 | 12 | 11 | 13 | 14 | 19 | 18 | 16 | 14 | 19 | 20 | 11 | 8 | 12 | 1 | 1 | 1 |
| S085 | Han | 35,39 | 15,15 | 12,17,19 | 21,22 | 16 | 12 | 13 | 29 | 25 | 10 | 13 | 12 | 15 | 11 | 11 | 14 | 25 | 19 | 31 | 15 | 19 | 11 | 24 | 36 | 12 | 11 | 11 | 14 | 19 | 15 | 16 | 15 | 24 | 20 | 10 | 8 | 13 | 1 | 2 | 2 |
| S086 | Han | 38,40 | 14,17 | 11,17 | 20,21 | 16 | 12 | 12 | 24 | 24 | 10 | 13 | 12 | 15 | 10 | 12 | 13 | 23 | 19 | 31 | 15 | 17 | 9 | 24 | 35 | 13 | 12 | 11 | 15 | 14 | 19 | 16 | 17 | 22 | 22 | 11 | 8 | 11 | 1 | 2 | 2 |
| S087 | Han | 36,39 | 15,17 | 19,19 | 16,22 | 14 | 12 | 13 | 30 | 24 | 10 | 11 | 13 | 14 | 10 | 11 | 12 | 26 | 20 | 34 | 16 | 17 | 11 | 22 | 42 | 13 | 12 | 14 | 18 | 18 | 17 | 16 | 14 | 22 | 20 | 13 | 8 | 11 | 2 | 2 | 2 |
| S088 | Han | 34,40 | 13,14 | 13,18 | 21,23 | 15 | 10 | 12 | 29 | 24 | 10 | 13 | 12 | 15 | 11 | 10 | 12 | 23 | 20 | 32 | 15 | 16 | 9 | 26 | 37 | 12 | 12 | 12 | 14 | 17 | 15 | 16 | 14 | 22 | 21 | 11 | 9 | 12 | 1 | 1 | 2 |
| S089 | Han | 35,38 | 14,16 | 13,19 | 18,24 | 17 | 12 | 13 | 28 | 25 | 10 | 13 | 12 | 14 | 10 | 13 | 13 | 22 | 19 | 29 | 14 | 18 | 11 | 24 | 37 | 12 | 11 | 11 | 11 | 18 | 18 | 16 | 15 | 21 | 24 | 11 | 8 | 13 | 1 | 2 | 2 |
| S090 | Han | 37,40 | 14,15 | 13,13 | 22,22 | 15 | 12 | 12 | 29 | 23 | 11 | 14 | 13 | 14 | 10 | 11 | 11 | 25 | 18 | 31 | 16 | 15 | 10 | 25 | 39 | 10 | 11 | 12 | 16 | 20 | 18 | 15 | 15 | 24 | 19 | 10 | 8 | 13 | 1 | 2 | 2 |
| S091 | Han | 37,38 | 14,15 | 11,17 | 20,21 | 15 | 13 | 13 | 31 | 24 | 10 | 11 | 14 | 14 | 10 | 11 | 13 | 26 | 21 | 29 | 15 | 16 | 11 | 24 | 37 | 12 | 12 | 12 | 17 | 17 | 18 | 17 | 15 | 23 | 23 | 9 | 8 | 11 | 2 | 2 | 2 |
| S092 | Han | 34,36 | 16,17 | 11,12 | 23,25 | 14 | 12 | 13 | 29 | 24 | 11 | 15 | 13 | 14 | 10 | 11 | 13 | 25 | 20 | 31 | 15 | 16 | 11 | 24 | 40 | 13 | 13 | 12 | 15 | 19 | 16 | 15 | 14 | 23 | 21 | 11 | 8 | 12 | 2 | 2 | 2 |
| S093 | Han | 39,39 | 14,14 | 15,22 | 21,21 | 13 | 12 | 13 | 29 | 24 | 9 | 14 | 14 | 14 | 12 | 11 | 13 | 27 | 19 | 32 | 16 | 17 | 10 | 24 | 38.2 | 10 | 12 | 12 | 19 | 20 | 18 | 15 | 16 | 21 | 22 | 10 | 8 | 10 | 2 | 2 | 2 |
| S094 | Han | 36,38 | 14,15 | 11,17 | 20,21 | 17 | 13 | 13 | 33 | 24 | 10 | 11 | 14 | 14 | 10 | 12 | 13 | 25 | 21 | 30 | 15 | 16 | 11 | 24 | 38 | 12 | 12 | 12 | 18 | 17 | 15 | 17 | 15 | 21 | 21 | 9 | 8 | 11 | 2 | 2 | 2 |
| S095 | Han | 40,40 | 13,13 | 12,17 | 21,21 | 15 | 12 | 14 | 31 | 24 | 10 | 13 | 14 | 14 | 10 | 11 | 13 | 27 | 18 | 29 | 15 | 16 | 11 | 23 | 38 | 11 | 11 | 12 | 17 | 20 | 17 | 15 | 15 | 21 | 21 | 11 | 8 | 11 | 1 | 2 | 2 |
| S096 | Han | 35,38 | 13,13 | 13,17,19 | 20,24 | 14 | 10 | 12 | 28 | 24 | 10 | 14 | 12 | 15 | 11 | 12 | 13 | 23 | 19 | 32 | 15 | 18 | 9 | 24 | 35 | 11 | 11 | 12 | 14 | 18 | 17 | 16 | 14 | 18 | 20 | 11 | 8 | 12 | 1 | 1 | 1 |
| S097 | Han | 36,39 | 14,15 | 13,18 | 23,23 | 16 | 12 | 12 | 28 | 24 | 10 | 13 | 12 | 14 | 10 | 12 | 12 | 24 | 19 | 31 | 14 | 19 | 11 | 23 | 40 | 13 | 11 | 12 | 17 | 19 | 16 | 16 | 15 | 20 | 23 | 10 | 8 | 11 | 1 | 2 | 2 |
| S098 | Han | 36,39 | 14,16 | 13,20 | 22,24 | 17 | 12 | 13 | 28 | 25 | 10 | 13 | 12 | 14 | 10 | 12 | 13 | 24 | 19 | 30 | 14 | 19 | 10 | 24 | 38 | 12 | 11 | 12 | 17 | 18 | 18 | 16 | 15 | 21 | 24 | 10 | 8 | 13 | 1 | 2 | 2 |
| S099 | Han | 36,39 | 13.2,14 | 12,18 | 21,22 | 15 | 12 | 13 | 29 | 25 | 11 | 13 | 12 | 15 | 10 | 11 | 14 | 24 | 19 | 30 | 13 | 18 | 9 | 24 | 38 | 12 | 11 | 12 | 15 | 19 | 17 | 16 | 15 | 21 | 22 | 9 | 8 | 13 | 1 | 2 | 2 |
| S100 | Han | 36,40 | 14,15 | 13,18 | 21,23 | 14 | 12 | 14 | 30 | 25 | 11 | 13 | 12 | 15 | 11 | 12 | 13 | 24 | 20 | 33 | 17 | 17 | 10 | 23 | 34 | 11 | 10 | 12 | 14 | 19 | 20 | 15 | 15 | 20 | 21 | 12 | 8 | 11 | 1 | 2 | 2 |
| S101 | Han | 38,39 | 14,15 | 12,13 | 21,23 | 14 | 12 | 12 | 28 | 23 | 11 | 14 | 13 | 14 | 10 | 11 | 11 | 24 | 18 | 31 | 18 | 16 | 10 | 25 | 39 | 10 | 11 | 12 | 15 | 18 | 18 | 15 | 15 | 23 | 19 | 11 | 8 | 12 | 1 | 2 | 2 |
| S102 | Han | 35,37 | 13.2,14 | 12,19 | 21,23 | 15 | 12 | 14 | 29 | 23 | 10 | 12 | 13 | 14 | 10 | 12 | 13 | 24 | 19 | 30 | 15 | 17 | 10 | 22 | 36 | 11 | 11 | 12 | 15 | 18 | 18 | 15 | 15 | 20 | 22 | 11 | 8 | 12 | 1 | 2 | 2 |
| S103 | Han | 35,39 | 13,14 | 13,19 | 20,24 | 14 | 10 | 12 | 29 | 23 | 10 | 14 | 12 | 15 | 11 | 12 | 14 | 23 | 19 | 33 | 14 | 20 | 9 | 23 | 35 | 11 | 12 | 15 | 14 | 18 | 19 | 16 | 14 | 19 | 20 | 12 | 8 | 12 | 1 | 1 | 1 |
| S104 | Han | 38,40 | 13,14 | 12,18 | 21,23 | 15 | 12 | 14 | 30 | 24 | 10 | 13 | 13 | 14 | 10 | 11 | 16 | 26 | 18 | 29 | 15 | 15 | 12 | 23 | 40 | 11 | 11 | 13 | 17 | 19 | 18 | 15 | 15 | 20 | 22 | 11 | 8 | 14 | 1 | 2 | 2 |
| S105 | Han | 38,38 | 15,15 | 15,23 | 21,21 | 13 | 12 | 14 | 30 | 24 | 9 | 15 | 14 | 14 | 12 | 11 | 12 | 27 | 18 | 29 | 17 | 17 | 10 | 24 | 39.2 | 10 | 12 | 11 | 18 | 19 | 19 | 15 | 16 | 20 | 22 | 10 | 8 | 10 | 2 | 2 | 2 |
| S106 | Han | 35,39 | 14,14 | 12,18 | 20,23 | 15 | 12 | 14 | 29 | 23 | 10 | 12 | 12 | 15 | 10 | 12 | 13 | 23 | 19 | 32 | 15 | 18 | 9 | 24 | 36 | 12 | 12 | 13 | 16 | 17 | 18 | 16 | 14 | 21 | 19 | 11 | 8 | 12 | 1 | 2 | 2 |
| S107 | Han | 35,38 | 12,13 | 13,17 | 21,23 | 14 | 10 | 12 | 28 | 24 | 11 | 14 | 12 | 15 | 11 | 11 | 12 | 23 | 20 | 34 | 16 | 17 | 9 | 23 | 34 | 11 | 11 | 12 | 13 | 18 | 18 | 16 | 14 | 18 | 20 | 11 | 8 | 13 | 1 | 1 | 1 |
| S108 | Han | 34,38 | 13,13 | 14,23 | 19,22 | 15 | 12 | 14 | 30 | 24 | 11 | 13 | 12 | 15 | 10 | 12 | 14 | 22 | 21 | 34 | 13 | 18 | 9 | 23 | 39 | 12 | 11 | 12 | 15 | 18 | 19 | 16 | 15 | 22 | 21 | 11 | 9 | 12 | 1 | 2 | 2 |
| S109 | Han | 37,38 | 12,13 | 13,21 | 21,21 | 16 | 12 | 12 | 29 | 25 | 10 | 13 | 12 | 15 | 10 | 13 | 13 | 23 | 20 | 32 | 14 | 16 | 10 | 24 | 39 | 12 | 11 | 13 | 14 | 18 | 17 | 17 | 15 | 20 | 21 | 11 | 8 | 12 | 1 | 2 | 2 |
| S110 | Han | 34,38 | 13,13 | 13,19 | 20,23 | 14 | 10 | 12 | 28 | 24 | 10 | 15 | 12 | 15 | 11 | 12 | 13 | 24 | 20 | 32 | 15 | 16 | 10 | 23 | 37 | 11 | 11 | 12 | 14 | 18 | 18 | 16 | 14 | 18 | 20 | 11 | 8 | 12 | 1 | 1 | 1 |
| S111 | Han | 39,40 | 13,13 | 15,17 | 25,26 | 15 | 12 | 12 | 29 | 25 | 10 | 10 | 12 | 14 | 10 | 11 | 13 | 26 | 19 | 29 | 16 | 22 | 11 | 27 | 36 | 12 | 12 | 11 | 15 | 20 | 20 | 15 | 16 | 22 | 20 | 9 | 8 | 11 | 2 | 2 | 2 |
| S112 | Han | 36,37 | 14,14 | 12,19 | 22,23 | 17 | 12 | 12 | 28 | 24 | 10 | 13 | 12 | 14 | 11 | 12 | 11 | 24 | 20 | 33 | 16 | 18 | 10 | 23 | 37 | 11 | 12 | 12 | 16 | 18 | 19 | 16 | 15 | 21 | 24 | 11 | 8 | 11 | 1 | 2 | 2 |
| S113 | Han | 35,40 | 13,14 | 12,18 | 22,24 | 15 | 12 | 12 | 30 | 23 | 10 | 13 | 12 | 14 | 10 | 12 | 12 | 23 | 19 | 33 | 15 | 19 | 9 | 21 | 37 | 14 | 11 | 13 | 15 | 16 | 18 | 16 | 14 | 19 | 19 | 12 | 8 | 12 | 1 | 2 | 2 |
| S114 | Han | 39,39 | 15,15 | 12,12 | 20,20 | 16 | 13 | 13 | 30 | 24 | 9 | 11 | 10 | 14 | 10 | 11 | 13 | 26 | 19 | 31 | 16 | 20 | 11 | 27 | 44 | 11 | 11 | 12 | 15 | 18 | 20 | 16 | 16 | 22 | 24 | 10 | 9 | 9 | 2 | 2 | 2 |
| S115 | Han | 36,38 | 14,15 | 11,17 | 19,21 | 16 | 13 | 13 | 30 | 23 | 10 | 11 | 15 | 14 | 10 | 11 | 12 | 25 | 21 | 31 | 15 | 18 | 11 | 24 | 35 | 14 | 13 | 13 | 15 | 16 | 17 | 17 | 15 | 22 | 22 | 9 | 8 | 11 | 2 | 2 | 2 |
| S116 | Han | 35,38 | 15,17 | 12,17 | 22,22 | 13 | 12 | 12 | 28 | 25 | 11 | 13 | 12 | 14 | 10 | 11 | 12 | 32 | 19 | 32 | 14 | 18 | 10 | 23 | 38 | 13 | 12 | 13 | 19 | 16 | 18 | 16 | 15 | 19 | 22 | 10 | 8 | 12 | 1 | 2 | 2 |
| S117 | Han | 37,38 | 12.2,16 | 13,17 | 22,24 | 15 | 14 | 14 | 31 | 23 | 10 | 13 | 14 | 14 | 10 | 11 | 13 | 27 | 18 | 27 | 14 | 20 | 10 | 23 | 41 | 12 | 10 | 12 | 14 | 18 | 18 | 15 | 15 | 21 | 21 | 12 | 8 | 10 | 1 | 2 | 2 |
| S118 | Han | 35,38 | 13,14 | 13,19 | 20,24 | 14 | 10 | 12 | 28 | 23 | 10 | 14 | 12 | 15 | 11 | 11 | 13 | 23 | 21 | 32 | 15 | 17 | 9 | 23 | 36 | 13 | 11 | 12 | 15 | 19 | 20 | 16 | 14 | 17.2 | 20 | 12 | 8 | 14 | 1 | 1 | 1 |
| S119 | Han | 35,40 | 13,14 | 11,21 | 20,21 | 14 | 10 | 12 | 28 | 24 | 10 | 14 | 12 | 15 | 11 | 11 | 13 | 24 | 20 | 30 | 15 | 18 | 9 | 21 | 37 | 9 | 11 | 11 | 14 | 18 | 19 | 16 | 13 | 19 | 20 | 10 | 8 | 12 | 1 | 1 | 1 |
| S120 | Han | 37,37 | 14,15 | 11,18 | 19,20 | 15 | 12 | 14 | 29 | 23 | 11 | 11 | 14 | 14 | 10 | 12 | 13 | 26 | 21 | 27 | 15 | 19 | 10 | 24 | 36 | 13 | 12 | 12 | 17 | 16 | 18 | 17 | 15 | 23 | 21 | 9 | 8 | 10 | 2 | 2 | 2 |
| S121 | Han | 36,38 | 15,17 | 12,19 | 22,24 | 15 | 12 | 12 | 28 | 24 | 10 | 13 | 12 | 14 | 10 | 11 | 12 | 24 | 19 | 32 | 15 | 20 | 10 | 23 | 39 | 13 | 11 | 12 | 15 | 15 | 18 | 16 | 15 | 19 | 22 | 10 | 8 | 11 | 1 | 2 | 2 |
| S122 | Han | 36,39 | 15,16 | 11,18 | 21,22 | 14 | 12 | 14 | 30 | 24 | 11 | 13 | 12 | 15 | 10 | 13 | 12 | 26 | 20 | 31 | 15 | 17 | 10 | 23 | 33 | 11 | 10 | 14 | 15 | 18 | 19 | 15 | 15 | 23 | 21 | 12 | 8 | 11 | 1 | 2 | 2 |
| S123 | Han | 35,38 | 13,15 | 11,17 | 19,20 | 16 | 12 | 14 | 29 | 23 | 10 | 10 | 14 | 14 | 10 | 12 | 13 | 27 | 21 | 29 | 15 | 17 | 10 | 24 | 37 | 11 | 11 | 12 | 17 | 16 | 17 | 17 | 15 | 23 | 22 | 9 | 8 | 10 | 2 | 2 | 2 |
| S124 | Han | 39,40 | 12,13 | 11,18 | 21,23 | 17 | 12 | 12 | 26 | 24 | 9 | 13 | 13 | 15 | 10 | 10 | 12 | 23 | 18 | 32 | 15 | 15 | 9 | 25 | 38 | 12 | 12 | 11 | 14 | 14 | 17 | 16 | 16 | 22 | 22 | 10 | 8 | 11 | 1 | 2 | 2 |
| S125 | Han | 36,36 | 13,16 | 13,16,20 | 22,23 | 15 | 12 | 14 | 30 | 23 | 11 | 14 | 13 | 14 | 10 | 12 | 14 | 27 | 18 | 25 | 14 | 19 | 10 | 23 | 42 | 13 | 10 | 12 | 14 | 18 | 19 | 15 | 15 | 21 | 22 | 10 | 8 | 10 | 1 | 2 | 2 |
| S126 | Han | 35,38 | 14,15 | 12,20 | 22,23 | 17 | 12 | 12 | 28 | 26 | 10 | 13 | 12 | 14 | 10 | 12 | 12 | 24 | 19 | 31 | 14 | 17 | 10 | 27 | 39 | 12 | 11 | 13 | 17 | 17 | 19 | 17 | 15 | 19 | 20 | 10 | 8 | 12 | 1 | 2 | 2 |
| S127 | Han | 36,39 | 14,15 | 14,20 | 22,23 | 15 | 12 | 15 | 31 | 23 | 10 | 14 | 13 | 14 | 10 | 12 | 13 | 27 | 18 | 31 | 15 | 18 | 10 | 23 | 40 | 12 | 10 | 12 | 14 | 16 | 19 | 15 | 15 | 23 | 24 | 12 | 8 | 10 | 1 | 2 | 2 |
| S128 | Han | 35,37 | 13,15 | 12,16 | 21,26 | 15 | 12 | 12 | 27 | 23 | 10 | 12 | 14 | 15 | 10 | 12 | 13 | 24 | 19 | 34 | 15 | 17 | 9 | 23 | 37 | 12 | 12 | 13 | 14 | 17 | 19 | 16 | 14 | 22 | 20 | 11 | 8 | 11 | 1 | 2 | 2 |
| S129 | Han | 38,40 | 13,14 | 12,17 | 20,22 | 15 | 12 | 14 | 30 | 24 | 10 | 13 | 14 | 14 | 10 | 11 | 14 | 27 | 18 | 29 | 15 | 17 | 11 | 23 | 39 | 11 | 11 | 12 | 17 | 21 | 19 | 15 | 15 | 20 | 20 | 11 | 8 | 13 | 1 | 2 | 2 |
| S130 | Han | 37,38 | 14,18 | 14,17 | 21,23 | 17 | 12 | 12 | 27 | 25 | 10 | 13 | 12 | 15 | 10 | 11 | 12 | 24 | 20 | 27 | 13 | 22 | 10 | 25 | 40 | 12 | 11 | 12 | 17 | 20 | 19 | 16 | 15 | 22 | 22 | 10 | 8 | 13 | 1 | 2 | 2 |
| S131 | Han | 37,39 | 14,15 | 12,13 | 22,22 | 15 | 12 | 13 | 30 | 23 | 10 | 12 | 12 | 15 | 11 | 15 | 14 | 23 | 20 | 35 | 15 | 18 | 10 | 20 | 38 | 11 | 11 | 13 | 16 | 19 | 17 | 16 | 14 | 19 | 24 | 11 | 8 | 12 | 1 | 2 | 2 |
| S132 | Han | 37,37 | 14,15 | 11,14 | 21,23 | 16 | 12 | 12 | 29 | 24 | 11 | 11 | 13 | 14 | 11 | 10 | 15 | 25 | 19 | 32 | 16 | 15 | 11 | 23 | 39 | 11 | 11 | 12 | 15 | 19 | 16 | 15 | 16 | 17 | 23 | 10 | 8 | 12 | 2 | 2 | 2 |
| S133 | Han | 38,39 | 13,14 | 11,12 | 20,21 | 15 | 14 | 13 | 29 | 21 | 11 | 11 | 13 | 14 | 10 | 11 | 12 | 28 | 20 | 27 | 18 | 17 | 12 | 27 | 39 | 12 | 12 | 10 | 15 | 19 | 17 | 15 | 16 | 22 | 21 | 10 | 8 | 13 | 2 | 2 | 2 |
| S134 | Han | 38,40 | 13,14 | 12,17 | 18,22 | 15 | 12 | 12 | 28 | 22 | 10 | 13 | 12 | 15 | 10 | 11 | 13 | 24 | 18 | 33 | 15 | 15 | 9 | 25 | 35 | 12 | 12 | 11 | 14 | 16 | 20 | 16 | 18 | 23 | 21 | 13 | 8 | 12 | 1 | 2 | 2 |
| S135 | Han | 37,41 | 14,15 | 13,17 | 21,23 | 15 | 12 | 14 | 31 | 24 | 10 | 13 | 14 | 14 | 10 | 11 | 14 | 27 | 18 | 28 | 15 | 15 | 11 | 26 | 38 | 11 | 11 | 12 | 17 | 20 | 18 | 15 | 15 | 20 | 21 | 12 | 8 | 12 | 1 | 2 | 2 |
| S136 | Han | 35,39 | 13,14 | 14,17 | 20,22 | 14 | 10 | 13 | 29 | 24 | 10 | 14 | 12 | 15 | 11 | 13 | 13 | 23 | 20 | 34 | 15 | 18 | 9 | 23 | 35 | 11 | 12 | 12 | 14 | 18 | 18 | 16 | 14 | 22 | 21 | 11 | 8 | 12 | 1 | 1 | 2 |
| S137 | Han | 36,38 | 14,15 | 12,14 | 23,24 | 16 | 12 | 12 | 27 | 25 | 11 | 13 | 12 | 14 | 10 | 12 | 12 | 24 | 19 | 32 | 14 | 18 | 11 | 25 | 42 | 12 | 11 | 13 | 16 | 18 | 20 | 16 | 15 | 20 | 22 | 10 | 8 | 11 | 1 | 2 | 2 |
| S138 | Han | 37,40 | 14,14 | 12,18 | 20,23 | 15 | 12 | 12 | 30 | 23 | 10 | 12 | 12 | 14 | 10 | 12 | 14 | 25 | 19 | 34 | 16 | 18 | 10 | 22 | 39 | 12 | 12 | 12 | 14 | 16 | 17 | 16 | 14 | 22 | 19 | 11 | 8 | 11 | 1 | 2 | 2 |
| S139 | Han | 36,38 | 12,14 | 10,17 | 19,23 | 16 | 13 | 13 | 30 | 22 | 10 | 11 | 14 | 14 | 10 | 11 | 12 | 27 | 21 | 30 | 15 | 15 | 11 | 24 | 40 | 11 | 11 | 13 | 17 | 15 | 16 | 17 | 15 | 21 | 22 | 9 | 8 | 11 | 2 | 2 | 2 |
| S140 | Han | 37,37 | 14,15 | 13,13 | 22,23 | 15 | 12 | 12 | 27 | 23 | 9 | 12 | 12 | 14 | 10 | 12 | 14 | 24 | 19 | 33 | 15 | 20 | 9 | 21 | 41 | 12 | 11 | 11 | 16 | 16 | 18 | 16 | 14 | 22 | 19 | 11 | 8 | 11 | 1 | 2 | 2 |
| S141 | Han | 37,38 | 15,17 | 11,20 | 19,22 | 16 | 12 | 14 | 29 | 23 | 11 | 11 | 14 | 14 | 10 | 14 | 12 | 27 | 22 | 28 | 15 | 18 | 11 | 25 | 36 | 11 | 12 | 12 | 17 | 16 | 16 | 17 | 15 | 21 | 21 | 9 | 8 | 10 | 2 | 2 | 2 |
| S142 | Han | 37,37 | 14,16 | 11,16 | 20,21 | 15 | 12 | 12 | 27 | 24 | 10 | 11 | 14 | 14 | 10 | 12 | 13 | 25 | 21 | 28 | 15 | 16 | 10 | 24 | 37 | 11 | 12 | 11 | 17 | 17 | 17 | 17 | 15 | 21 | 21 | 10 | 8 | 11 | 2 | 2 | 2 |
| S143 | Han | 35,38 | 13,13 | 14,19 | 21,22 | 14 | 10 | 12 | 29 | 24 | 10 | 14 | 13 | 15 | 11 | 14 | 12 | 25 | 19 | 36 | 15 | 18 | 9 | 23 | 37 | 11 | 11 | 13 | 14 | 21 | 19 | 16 | 14 | 17 | 21 | 11 | 8 | 12 | 1 | 1 | 1 |
| S144 | Han | 39,40 | 14,15 | 12,12 | 19,20 | 17 | 15 | 13 | 29 | 25 | 10 | 11 | 13 | 14 | 10 | 10 | 15 | 26 | 22 | 28 | 15 | 18 | 10 | 25 | 39 | 11 | 12 | 13 | 15 | 16 | 18 | 16 | 16 | 21 | 21 | 10 | 8 | 11 | 2 | 2 | 2 |
| S145 | Han | 35,40 | 13,15 | 17,17 | 21,24 | 15 | 10 | 12 | 28 | 24 | 11 | 13 | 12 | 15 | 11 | 12 | 13 | 23 | 20 | 32 | 16 | 15 | 10 | 26 | 39 | 12 | 11 | 13 | 15 | 17 | 18 | 16 | 14 | 20 | 20 | 12 | 8 | 12 | 1 | 1 | 2 |
| S146 | Han | 37,38 | 16,17 | 12,17 | 20,26 | 14 | 13 | 13 | 29 | 24 | 10 | 10 | 14 | 16 | 11 | 10 | 12 | 27 | 19 | 32 | 15 | 17 | 10 | 25 | 39 | 11 | 11 | 13 | 14 | 17 | 14 | 15 | 16 | 19 | 24 | 10 | 8 | 12 | 2 | 2 | 2 |
| S147 | Han | 36,39 | 15,15 | 12,20 | 22,24 | 16 | 12 | 12 | 28 | 24 | 9 | 13 | 12 | 14 | 10 | 10 | 12 | 24 | 19 | 31 | 15 | 17 | 10 | 26 | 39 | 12 | 11 | 13 | 17 | 17 | 19 | 17 | 15 | 21 | 21 | 10 | 8 | 11 | 1 | 2 | 2 |
| S148 | Han | 35,39 | 13,13 | 13,16 | 20,24 | 15 | 10 | 12 | 28 | 24 | 10 | 14 | 12 | 14 | 11 | 12 | 13 | 23 | 20 | 32 | 14 | 19 | 10 | 25 | 35 | 11 | 11 | 12 | 14 | 18 | 17 | 16 | 14 | 18 | 20 | 12 | 8 | 11 | 1 | 1 | 1 |
| S149 | Han | 35,40 | 14,17 | 11,12 | 20,23 | 15 | 12 | 13 | 28 | 24 | 11 | 15 | 13 | 14 | 10 | 12 | 13 | 25 | 19 | 32 | 15 | 17 | 11 | 24 | 39 | 12 | 12 | 12 | 15 | 20 | 17 | 15 | 14 | 23 | 22 | 11 | 8 | 12 | 2 | 2 | 2 |
| S150 | Han | 34,37 | 13,15 | 12,17 | 23,25 | 16 | 12 | 12 | 28 | 25 | 10 | 13 | 14 | 14 | 10 | 12 | 13 | 24 | 19 | 29 | 15 | 19 | 10 | 26 | 41 | 13 | 11 | 14 | 17 | 20 | 16 | 16 | 15 | 21 | 22 | 10 | 8 | 12 | 1 | 2 | 2 |
| S151 | Han | 36,38 | 15,15 | 12,19 | 20,24 | 16 | 12 | 12 | 27 | 25 | 10 | 13 | 12 | 14 | 10 | 12 | 12 | 24 | 19 | 35 | 14 | 19 | 10 | 25 | 38 | 11 | 11 | 12 | 15 | 17 | 17 | 16 | 15 | 19 | 24 | 10 | 8 | 11 | 1 | 2 | 2 |
| S152 | Han | 37,40 | 14,16 | 13,13 | 21,22 | 15 | 12 | 13 | 30 | 25 | 10 | 13 | 13 | 15 | 10 | 11 | 14 | 24 | 20 | 33 | 15 | 16 | 9 | 24 | 39 | 11 | 11 | 12 | 15 | 18 | 19 | 16 | 15 | 22 | 20 | 12 | 8 | 11 | 1 | 2 | 2 |
| S153 | Han | 36,38 | 13,16 | 12,20 | 23,24 | 15 | 12 | 12 | 28 | 25 | 11 | 13 | 12 | 14 | 10 | 11 | 12 | 23 | 19 | 31 | 14 | 18 | 10 | 25 | 38 | 12 | 11 | 12 | 15 | 17 | 16 | 16 | 15 | 21 | 22 | 10 | 8 | 11 | 1 | 2 | 2 |
| S154 | Han | 36,38 | 14,15 | 12,15 | 23,23 | 16 | 12 | 12 | 27 | 25 | 11 | 13 | 12 | 14 | 10 | 12 | 12 | 24 | 19 | 32 | 14 | 17 | 11 | 23 | 41 | 12 | 11 | 13 | 16 | 18 | 20 | 16 | 15 | 20 | 23 | 10 | 8 | 11 | 1 | 2 | 2 |
| S155 | Han | 38,38 | 15,15 | 11,18 | 20,20 | 16 | 13 | 13 | 30 | 24 | 10 | 11 | 15 | 14 | 10 | 11 | 15 | 25 | 21 | 28 | 15 | 16 | 10 | 25 | 38 | 12 | 12 | 12 | 20 | 17 | 17 | 17 | 14 | 19 | 22 | 8 | 8 | 12 | 2 | 2 | 2 |
| S156 | Han | 36,41 | 13,16 | 12,17 | 20,24 | 15 | 12 | 12 | 29 | 23 | 9 | 12 | 12 | 14 | 10 | 11 | 14 | 26 | 19 | 34 | 17 | 18 | 11 | 21 | 38 | 12 | 11 | 12 | 14 | 16 | 21 | 16 | 14 | 21 | 19 | 11 | 8 | 13 | 1 | 2 | 2 |
| S157 | Han | 35,39 | 13,13 | 13,19 | 20,23 | 14 | 10 | 12 | 27 | 23 | 10 | 14 | 12 | 14 | 11 | 12 | 13 | 23 | 20 | 34 | 14 | 17 | 9 | 22 | 37 | 11 | 11 | 12 | 13 | 19 | 20 | 16 | 14 | 19 | 20 | 11 | 8 | 12 | 1 | 1 | 1 |
| S158 | Han | 36,38 | 14,16 | 11,19 | 19,20 | 15 | 12 | 13 | 28 | 23 | 10 | 11 | 14 | 14 | 10 | 13 | 12 | 26 | 21 | 30 | 15 | 17 | 11 | 24 | 37 | 11 | 12 | 12 | 17 | 16 | 17 | 17 | 15 | 24 | 22 | 9 | 8 | 11 | 2 | 2 | 2 |
| S159 | Han | 35,35 | 14,14 | 11,13 | 20.2,20.2 | 14 | 12 | 14 | 30 | 23 | 11 | 14 | 13 | 14 | 10 | 10 | 13 | 24.3 | 18 | 29 | 14 | 17 | 10 | 21 | 38 | 12 | 13 | 12 | 14 | 19 | 19 | 14.3 | 13.5 | 20 | 20 | 11 | 7.2 | 11 | 2 | 2 | 2 |
| S160 | Han | 37,37 | 11,14 | 13,13 | 21,22 | 15 | 12 | 12 | 30 | 24 | 10 | 13 | 12 | 15 | 11 | 12 | 14 | 23 | 21 | 33 | 14 | 17 | 11 | 24 | 39 | 12 | 12 | 13 | 14 | 18 | 18 | 17 | 14 | 19 | 23 | 11 | 8 | 12 | 1 | 2 | 2 |
| S161 | Han | 34,40 | 13,13 | 12,17 | 21,24 | 14 | 10 | 12 | 28 | 24 | 10 | 13 | 12 | 15 | 11 | 13 | 12 | 23 | 20 | 30 | 16 | 16 | 10 | 25 | 40 | 12 | 11 | 12 | 15 | 17 | 17 | 16 | 14 | 21 | 21 | 11 | 8 | 12 | 1 | 1 | 2 |
| S162 | Han | 36,41 | 13,14 | 11,11 | 21,21 | 16 | 12 | 13 | 28 | 22 | 10 | 14 | 13 | 15 | 10 | 11 | 12 | 26 | 19 | 32 | 15 | 18 | 9 | 22 | 38 | 12 | 11 | 13 | 15 | 18 | 17 | 16 | 15 | 21 | 20 | 10 | 8 | 12 | 1 | 2 | 2 |
| S163 | Han | 36,38 | 13,14 | 13,19 | 20,22 | 14 | 10 | 12 | 29 | 24 | 10 | 14 | 12 | 15 | 11 | 11 | 14 | 25 | 20 | 34 | 13 | 17 | 9 | 22 | 35 | 11 | 11 | 13 | 14 | 19 | 16 | 16 | 14 | 17 | 20 | 11 | 8 | 12 | 1 | 1 | 1 |
| S164 | Han | 37,39 | 11,13 | 12,16 | 22,22 | 15 | 12 | 12 | 29 | 23 | 10 | 12 | 12 | 15 | 10 | 11 | 14 | 25 | 19 | 32 | 15 | 18 | 10 | 22 | 38 | 12 | 11 | 12 | 15 | 16 | 22 | 16 | 14 | 22 | 19 | 11 | 8 | 13 | 1 | 2 | 2 |
| S165 | Han | 35,38 | 13,14 | 13,21 | 20,25 | 14 | 10 | 12 | 28 | 24 | 10 | 14 | 12 | 14 | 11 | 12 | 13 | 23 | 20 | 33 | 14 | 17 | 9 | 23 | 36 | 11 | 11 | 12 | 14 | 18 | 18 | 16 | 14 | 18 | 20 | 11 | 8 | 12 | 1 | 1 | 1 |
| S166 | Han | 35,39 | 13,15 | 14,21 | 20,23 | 15 | 10 | 12 | 28 | 24 | 10 | 14 | 12 | 14 | 11 | 12 | 11 | 23 | 20 | 32 | 15 | 17 | 9 | 23 | 35 | 11 | 11 | 12 | 15 | 18 | 18 | 16 | 14 | 17 | 21 | 11 | 8 | 12 | 1 | 1 | 1 |
| S167 | Han | 35,38 | 14,15 | 13,20 | 21,24 | 14 | 10 | 12 | 28 | 24 | 9 | 14 | 12 | 15 | 11 | 13 | 12 | 23 | 19 | 32 | 15 | 17 | 9 | 23 | 33 | 12 | 10 | 12 | 13 | 18 | 18 | 16 | 14 | 18 | 20 | 11 | 8 | 13 | 1 | 1 | 1 |
| S168 | Han | 35,38 | 13,14 | 13,19 | 20,23 | 14 | 10 | 12 | 28 | 23 | 10 | 14 | 12 | 14 | 11 | 11 | 13 | 24 | 20 | 35 | 16 | 17 | 9 | 21 | 36 | 11 | 11 | 13 | 14 | 18 | 18 | 16 | 14 | 18 | 20 | 11 | 8 | 12 | 1 | 1 | 1 |
| S169 | Han | 35,39 | 14,16 | 12,13 | 20,22 | 15 | 12 | 12 | 28 | 23 | 10 | 12 | 12 | 16 | 10 | 12 | 12 | 24 | 18 | 35 | 16 | 18 | 10 | 22 | 36 | 12 | 11 | 12 | 15 | 16 | 18 | 16 | 14 | 22.1 | 20 | 10 | 8 | 12 | 1 | 2 | 2 |
| S170 | Han | 38,39 | 14,15 | 12,19 | 22,22 | 15 | 12 | 12 | 28 | 25 | 10 | 13 | 12 | 14 | 10 | 13 | 12 | 24 | 19 | 32 | 14 | 16 | 10 | 25 | 37 | 11 | 10 | 12 | 16 | 18 | 18 | 16 | 15 | 20 | 23 | 8 | 8 | 12 | 1 | 2 | 2 |
| S171 | Han | 36,40 | 13.2,14 | 12,18 | 21,23 | 15 | 12 | 13 | 31 | 24 | 10 | 13 | 12 | 15 | 10 | 11 | 12 | 24 | 20 | 30 | 14 | 17 | 10 | 24 | 38 | 12 | 11 | 12 | 15 | 20 | 17 | 16 | 15 | 20 | 24 | 10 | 8 | 13 | 1 | 2 | 2 |
| S172 | Han | 35,35 | 16,16 | 11,18 | 19,19 | 15 | 13 | 14 | 30 | 23 | 11 | 11 | 16 | 14 | 10 | 11 | 15 | 25 | 21 | 29 | 14 | 16 | 10 | 26 | 39 | 11 | 12 | 12 | 19 | 15 | 18 | 17 | 15 | 20 | 22 | 10 | 8 | 11 | 2 | 2 | 2 |
| S173 | Han | 38,38 | 14,16 | 13,20 | 22,23 | 17 | 12 | 13 | 28 | 23 | 11 | 13 | 12 | 14 | 10 | 12 | 12 | 24 | 19 | 33 | 14 | 18 | 10 | 25 | 41 | 12 | 11 | 13 | 16 | 18 | 17 | 17 | 15 | 19 | 21 | 10 | 8 | 12 | 1 | 2 | 2 |
| S174 | Han | 38,39 | 14,15,16 | 14,17 | 21,22 | 13 | 16 | 13 | 29 | 23 | 10 | 11 | 12 | 15 | 9 | 12 | 11 | 23 | 21 | 32 | 15 | 16 | 10 | 21 | 38 | 11 | 11 | 12 | 13 | 19 | 15 | 15 | 15 | 21 | 22 | 10 | 8 | 12 | 2 | 2 | 2 |
| S175 | Han | 38,39 | 13,13 | 12,16 | 20.2,22.2 | 15 | 12 | 14 | 30 | 24 | 10 | 13 | 14 | 14 | 10 | 11 | 13 | 26.3 | 18 | 30 | 14 | 15 | 11 | 23 | 39 | 11 | 10 | 13 | 18 | 20 | 18 | 14.3 | 14.5 | 22 | 22 | 11 | 7.2 | 12 | 1 | 2 | 2 |
| S176 | Han | 36,37 | 14,15 | 12,19 | 22,23 | 15 | 12 | 12 | 28 | 25 | 10 | 13 | 12 | 14 | 10 | 12 | 12 | 24 | 19 | 32 | 14 | 17 | 10 | 26 | 39 | 12 | 11 | 13 | 16 | 19 | 21 | 17 | 15 | 20 | 20 | 10 | 8 | 12 | 1 | 2 | 2 |
| S177 | Han | 37,37 | 14,14 | 13,19 | 21,23 | 15 | 10 | 12 | 28 | 24 | 10 | 14 | 12 | 14 | 11 | 13 | 12 | 23 | 20 | 37 | 15 | 16 | 11 | 23 | 35 | 11 | 11 | 13 | 13 | 17 | 18 | 16 | 14 | 18 | 21 | 11 | 8 | 13 | 1 | 1 | 1 |
| S178 | Han | 38.3,42.2 | 11,14 | 12,20 | 22,25 | 15 | 12 | 12 | 28 | 24 | 9 | 13 | 12 | 15 | 9 | 10 | 11.3 | 23 | 19 | 28 | 16 | 15 | 9 | 24 | 35 | 14 | 12 | 13 | 15 | 14 | 16.3 | 16 | 16 | 22 | 21 | 12 | 9 | 13 | 1 | 2 | 2 |
| S179 | Han | 36,36 | 14,14 | 11,12 | 22,22 | 14 | 13 | 14 | 31 | 25 | 10 | 14 | 13 | 14 | 10 | 11 | 13 | 25 | 19 | 30 | 17 | 17 | 11 | 22 | 37 | 12 | 12 | 12 | 14 | 19 | 17 | 15 | 14 | 21 | 23 | 10 | 8 | 12 | 2 | 2 | 2 |
| S180 | Han | 37,38 | 15,18 | 14,18 | 22,23 | 12 | 12 | 12 | 28 | 25 | 11 | 13 | 12 | 14 | 10 | 12 | 12 | 24 | 19 | 27 | 14 | 18 | 10 | 25 | 40 | 12 | 10 | 13 | 16 | 19.3 | 17 | 16 | 15 | 22 | 23 | 10 | 8 | 12 | 1 | 2 | 2 |
| S181 | Han | 38,38 | 13,13 | 15,15 | 23.2,23.2 | 15 | 12 | 12 | 28 | 24 | 10 | 10 | 12 | 14 | 10 | 11 | 13 | 26.3 | 19 | 30 | 15 | 20 | 11 | 29 | 36 | 11 | 13 | 11 | 14 | 20 | 18 | 14.3 | 16 | 23 | 20 | 9 | 7.2 | 11 | 2 | 2 | 2 |
| S182 | Han | 36,39 | 14,15 | 11,11 | 21,22 | 16 | 14 | 14 | 29 | 21 | 10 | 14 | 12 | 15 | 10 | 11 | 11 | 28 | 20 | 32 | 14 | 17 | 9 | 23 | 40 | 13 | 11 | 13 | 16 | 18 | 18 | 16 | 15 | 22 | 22 | 10 | 8 | 11 | 1 | 2 | 2 |
| S183 | Han | 36,37 | 13,13 | 12,17 | 19,24 | 16 | 12 | 12 | 28 | 23 | 10 | 12 | 12 | 15 | 10 | 13 | 13 | 24 | 19 | 32 | 15 | 18 | 9 | 23 | 38 | 13 | 12 | 12 | 15 | 19 | 19 | 16 | 14 | 25 | 19 | 11 | 8 | 11 | 1 | 2 | 2 |
| S184 | Han | 34,36 | 17,17 | 11,12 | 23,25 | 14 | 12 | 13 | 29 | 24 | 11 | 15 | 13 | 14 | 10 | 11 | 13 | 25 | 20 | 31 | 15 | 16 | 11 | 24 | 40 | 13 | 13 | 12 | 15 | 19 | 16 | 15 | 14 | 23 | 21 | 11 | 8 | 12 | 2 | 2 | 2 |
| S185 | Han | 38,38 | 13,16 | 12,21 | 22,22 | 16 | 12 | 12 | 28 | 24 | 11 | 13 | 12 | 15 | 11 | 11 | 12 | 23 | 19 | 32 | 15 | 17 | 10 | 23 | 42 | 11 | 11 | 12 | 17 | 17 | 16 | 16 | 15 | 19 | 21 | 10 | 8 | 12 | 1 | 2 | 2 |
| S186 | Han | 36,37 | 14,15 | 11,18 | 19,20 | 15 | 12 | 14 | 29 | 23 | 11 | 11 | 14 | 14 | 10 | 12 | 13 | 26 | 21 | 28 | 15 | 18 | 11 | 24 | 35 | 13 | 12 | 12 | 17 | 16 | 17 | 17 | 15 | 20 | 21 | 9 | 8 | 11 | 2 | 2 | 2 |
| S187 | Han | 38,38 | 13,13 | 15,15 | 23,23 | 15 | 12 | 13 | 29 | 24 | 10 | 10 | 12 | 14 | 10 | 11 | 12 | 27 | 19 | 31 | 15 | 21 | 11 | 28 | 38 | 11 | 12 | 11 | 14 | 20 | 16 | 15 | 16 | 25 | 20 | 9 | 8 | 12 | 2 | 2 | 2 |
| S188 | Han | 35,39 | 13,13 | 15,17 | 22,24 | 14 | 10 | 12 | 28 | 24 | 10 | 14 | 12 | 14 | 11 | 12 | 9 | 23 | 19 | 37 | 15 | 18 | 9 | 23 | 37 | 11 | 11 | 12 | 14 | 18 | 18 | 16 | 14 | 20 | 20 | 11 | 8 | 12 | 1 | 1 | 1 |
| S189 | Han | 38,39 | 13,13 | 12,19 | 19,23 | 15 | 12 | 13 | 29 | 25 | 10 | 13 | 12 | 15 | 10 | 12 | 13 | 23 | 20 | 34 | 15 | 19 | 9 | 24 | 37 | 12 | 12 | 12 | 13 | 15 | 19 | 16 | 17 | 20 | 22 | 11 | 8 | 12 | 1 | 2 | 2 |
| S190 | Han | 37,38 | 16,16 | 14,18 | 23,23 | 14 | 12 | 12 | 28 | 25 | 10 | 13 | 12 | 14 | 10 | 11 | 12 | 25 | 20 | 27 | 15 | 17 | 10 | 24 | 38 | 12 | 11 | 14 | 15 | 18 | 16 | 16 | 15 | 21 | 22 | 10 | 8 | 12 | 1 | 2 | 2 |
| S191 | Han | 36,41 | 12,13 | 12,18 | 21,22 | 14 | 10 | 13 | 29 | 24 | 10 | 13 | 12 | 15 | 11 | 13 | 14 | 24 | 20 | 32 | 15 | 15 | 9 | 24 | 37 | 12 | 11 | 12 | 14 | 17 | 19 | 16 | 14 | 20 | 22 | 11 | 8 | 11 | 1 | 1 | 2 |
| S192 | Han | 37,37 | 14,16 | 11,16 | 20,21 | 15 | 12 | 13 | 28 | 24 | 10 | 11 | 14 | 14 | 10 | 12 | 13 | 25 | 21 | 28 | 15 | 16 | 10 | 24 | 37 | 11 | 12 | 11 | 18 | 17 | 17 | 17 | 15 | 22 | 21 | 10 | 8 | 11 | 2 | 2 | 2 |
| S193 | Han | 34,36 | 13,13 | 13,18 | 20,23 | 13 | 10 | 13 | 29 | 25 | 10 | 16 | 12 | 15 | 11 | 12 | 13 | 23 | 20 | 32 | 18 | 18 | 11 | 23 | 35 | 11 | 11 | 12 | 14 | 19 | 21 | 16 | 14 | 18 | 20 | 11 | 8 | 12 | 1 | 1 | 1 |
| S194 | Han | 35,36 | 14,16 | 11,18 | 19,21 | 15 | 13 | 13 | 29 | 23 | 11 | 11 | 16 | 14 | 10 | 12 | 12 | 26 | 21 | 28 | 15 | 17 | 11 | 27 | 38 | 11 | 12 | 13 | 17 | 16 | 18 | 17 | 15 | 24 | 22 | 9 | 8 | 12 | 2 | 2 | 2 |
| S195 | Han | 38,38 | 14,16 | 11,14 | 21,23 | 16 | 12 | 13 | 31 | 22 | 11 | 11 | 13 | 15 | 11 | 10 | 14 | 24 | 20 | 32 | 16 | 16 | 11 | 23 | 41 | 10 | 12 | 12 | 15 | 19 | 18 | 15 | 16 | 18 | 23 | 10 | 8 | 13 | 2 | 2 | 2 |
| S196 | Han | 37,37 | 14,15 | 11,18 | 19,20 | 15 | 12 | 14 | 29 | 23 | 10 | 11 | 14 | 14 | 10 | 12 | 12 | 26 | 21 | 29 | 15 | 19 | 10 | 24 | 36 | 12 | 12 | 12 | 17 | 16 | 17 | 17 | 15 | 23 | 21 | 9 | 8 | 11 | 2 | 2 | 2 |
| S197 | Han | 36,42.3 | 15,15 | 12,17 | 21,21 | 15 | 12 | 13 | 28 | 23 | 10 | 12 | 12 | 15 | 10 | 12 | 12 | 24 | 20 | 33.1 | 15 | 17 | 9 | 23 | 37 | 11 | 11 | 12 | 16 | 19 | 19.2 | 16 | 14 | 22 | 20 | 12 | 8 | 12 | 1 | 2 | 2 |
| S198 | Han | 37,39 | 14,16 | 11,17 | 18,20 | 15 | 13 | 14 | 30 | 23 | 10 | 13 | 14 | 14 | 10 | 11 | 13 | 25 | 21 | 30 | 15 | 15 | 11 | 24 | 39 | 11 | 12 | 12 | 19 | 16 | 19 | 17 | 15 | 21 | 23 | 8 | 8 | 11 | 2 | 2 | 2 |
| S199 | Han | 35,39 | 13,14 | 15,19 | 20,23 | 14 | 10 | 12 | 28 | 23 | 10 | 14 | 12 | 15 | 11 | 11 | 14 | 23 | 20 | 32 | 15 | 18 | 9 | 24 | 37 | 11 | 11 | 13 | 14 | 17 | 18 | 16 | 14 | 21 | 21 | 11 | 8 | 12 | 1 | 1 | 1 |
| S200 | Han | 37,37 | 14,15 | 13,13 | 22,23 | 15 | 12 | 12 | 27 | 23 | 9 | 12 | 12 | 14 | 10 | 13 | 14 | 25 | 19 | 33 | 15 | 20 | 9 | 21 | 39 | 12 | 11 | 11 | 16 | 16 | 19 | 16 | 14 | 22 | 19 | 11 | 8 | 11 | 1 | 2 | 2 |
| S201 | Han | 37,38 | 13,14 | 13,22 | 21,21 | 16 | 12 | 12 | 30 | 24 | 10 | 13 | 12 | 15 | 10 | 12 | 14 | 23 | 20 | 32 | 14 | 15 | 10 | 24 | 39 | 12 | 11 | 13 | 14 | 18 | 18 | 17 | 15 | 20 | 21 | 11 | 8 | 13 | 1 | 2 | 2 |
| S202 | Han | 38,38 | 14,14 | 11,13 | 22,22 | 14 | 13 | 13 | 28 | 23 | 11 | 14 | 13 | 14 | 11 | 11 | 12 | 25 | 19 | 28 | 16 | 16 | 11 | 23 | 36 | 12 | 12 | 12 | 14 | 16 | 19 | 15 | 14 | 21 | 21 | 10 | 8 | 12 | 2 | 2 | 2 |
| S203 | Han | 36,37 | 13,13 | 16,16 | 17.3,24.3 | 15 | 12 | 12 | 29 | 25 | 10 | 10 | 12 | 15 | 10 | 11 | 13 | 26.3 | 19 | 29 | 15 | 21 | 11 | 28 | 36 | 11 | 12 | 12 | 15 | 19 | 17 | 14.3 | 16 | 21 | 22 | 9 | 7.3 | 11 | 2 | 2 | 2 |
| S204 | Han | 37,37 | 15,15 | 14,14 | 20.2,20.2 | 16 | 12 | 14 | 30 | 22 | 10 | 14 | 13 | 14 | 10 | 11 | 12 | 25.3 | 19 | 31 | 15 | 18 | 10 | 23 | 36 | 12 | 12 | 12 | 14 | 20 | 20 | 14.3 | 13.5 | 22 | 21 | 10 | 7.3 | 13 | 2 | 2 | 2 |
| S205 | Han | 38,40 | 14,15 | 13,14 | 21,24 | 15 | 12 | 12 | 29 | 23 | 10 | 14 | 13 | 14 | 10 | 11 | 11 | 25 | 18 | 31 | 16 | 15 | 10 | 25 | 41 | 10 | 11 | 11 | 15 | 17 | 18 | 15 | 15 | 23 | 19 | 11 | 8 | 12 | 1 | 2 | 2 |
| S206 | Han | 36,36 | 13,16 | 11,12 | 19.2,20.2 | 14 | 12 | 14 | 30 | 23 | 11 | 14 | 12 | 14 | 10 | 10 | 14 | 25.3 | 18 | 31 | 14 | 18 | 10 | 21 | 37 | 11 | 12 | 11 | 14 | 19 | 16 | 14.3 | 14.5 | 23 | 20 | 12 | 7.3 | 11 | 2 | 2 | 2 |
| S207 | Han | 35,39 | 12,14 | 14,19 | 20,24 | 15 | 10 | 12 | 28 | 25 | 10 | 14 | 12 | 15 | 11 | 11 | 13 | 23 | 19 | 32 | 15 | 18 | 9 | 23 | 36 | 10 | 12 | 12 | 14 | 19 | 18 | 16 | 14 | 18 | 20 | 11 | 8 | 12 | 1 | 1 | 1 |
| S208 | Han | 36,37 | 14,17 | 11,17 | 19,21 | 15 | 12 | 14 | 29 | 23 | 10 | 11 | 14 | 14 | 10 | 12 | 14 | 26 | 21 | 31 | 15 | 17 | 10 | 24 | 37 | 11 | 12 | 11 | 16 | 16 | 17 | 17 | 15 | 21 | 20 | 9 | 8 | 11 | 2 | 2 | 2 |
| S209 | Han | 35,39 | 14,15,16 | 11,12 | 19.2,22.2 | 14 | 12 | 13 | 28 | 23 | 11 | 15 | 13 | 14 | 10 | 10 | 12 | 24.3 | 19 | 31 | 15 | 17 | 11 | 24 | 41 | 12 | 12 | 12 | 15 | 19 | 16 | 14.3 | 13.5 | 20 | 22 | 11 | 7.3 | 13 | 1 | 2 | 2 |
| S210 | Han | 35,37 | 12,15 | 14,18 | 19,25 | 14 | 10 | 12 | 28 | 24 | 10 | 14 | 12 | 15 | 11 | 12 | 13 | 23 | 20 | 30 | 16 | 19 | 10 | 23 | 37 | 11 | 11 | 12 | 14 | 18 | 18 | 17 | 14 | 22 | 20 | 11 | 8 | 13 | 1 | 1 | 1 |
| S211 | Han | 37,37 | 13.2,13.2 | 12,20 | 22,22 | 17 | 12 | 12 | 27 | 24 | 10 | 13 | 12 | 14 | 10 | 12 | 12.2 | 25 | 19 | 30 | 14 | 20 | 10 | 25 | 39 | 13.3 | 10 | 12 | 16.2 | 17 | 18 | 16 | 15 | 20 | 21 | 10 | 8 | 11 | 1 | 2 | 2 |
| S212 | Han | 35,39 | 16,16 | 14,18 | 23,24 | 17 | 12 | 12 | 28 | 24 | 10 | 13 | 14 | 14 | 10 | 11 | 12 | 24 | 20 | 27 | 13 | 19 | 10 | 25 | 38 | 12 | 11 | 13 | 16 | 19 | 16 | 16 | 15 | 22 | 24 | 10 | 8 | 12 | 1 | 2 | 2 |
| S213 | Han | 36,41 | 12,13 | 12,18 | 21,21 | 16 | 10 | 13 | 28 | 24 | 10 | 13 | 12 | 15 | 11 | 13 | 14 | 24 | 20 | 32 | 15 | 15 | 10 | 24 | 37 | 12 | 11 | 12 | 14 | 17 | 18 | 16 | 14 | 20 | 22 | 11 | 8 | 11 | 1 | 1 | 2 |
| S214 | Han | 36,36 | 15,15 | 11,12 | 20,20 | 14 | 12 | 14 | 31 | 23 | 11 | 14 | 12 | 14 | 10 | 10 | 14 | 26 | 18 | 30 | 14 | 18 | 10 | 22 | 37 | 11 | 12 | 12 | 14 | 19 | 17 | 15 | 15 | 22 | 20 | 12 | 8 | 11 | 1 | 2 | 2 |
| S215 | Han | 36,38 | 16,16 | 11,14 | 21,25 | 16 | 12 | 14 | 30 | 25 | 10 | 11 | 13 | 14 | 11 | 11 | 13 | 24 | 20 | 33 | 16 | 15 | 11 | 24 | 40 | 11 | 13 | 12 | 16 | 20 | 18 | 16 | 15 | 19 | 23 | 10 | 8 | 13 | 1 | 2 | 2 |
| S216 | Han | 38,38 | 16,16 | 11,17 | 21,21 | 16 | 14 | 13 | 29 | 23 | 11 | 11 | 15 | 14 | 10 | 11 | 13 | 25 | 21 | 30 | 14 | 17 | 11 | 24 | 37 | 11 | 12 | 12 | 17 | 18 | 18 | 17 | 15 | 23 | 21 | 10 | 8 | 11 | 1 | 2 | 2 |
| S217 | Han | 39,40 | 15,16 | 12,12 | 19,20 | 15 | 14 | 13 | 29 | 24 | 10 | 11 | 14 | 14 | 10 | 10 | 13 | 26 | 22 | 28 | 15 | 18 | 10 | 25 | 40 | 11 | 12 | 12 | 15 | 16 | 17 | 16 | 16 | 23 | 22 | 10 | 8 | 11 | 2 | 2 | 2 |
| S218 | Han | 36,37 | 14,16 | 11,19 | 19,20 | 15 | 12 | 14 | 29 | 23 | 11 | 11 | 14 | 14 | 10 | 12 | 13 | 26 | 21 | 29 | 15 | 17 | 10 | 24 | 35 | 12 | 12 | 12 | 17 | 16 | 17 | 17 | 15 | 24 | 21 | 9 | 8 | 11 | 2 | 2 | 2 |
| S219 | Han | 36,38 | 15,16 | 11,19 | 20,21 | 16 | 13 | 14 | 30 | 24 | 10 | 11 | 14 | 14 | 10 | 12 | 15 | 26 | 22 | 29 | 15 | 15 | 11 | 28 | 36 | 11 | 14 | 13 | 20 | 17 | 18 | 17 | 15 | 19 | 21 | 8 | 8 | 11 | 2 | 2 | 2 |
| S220 | Han | 38,40 | 15,16 | 13,15 | 23,24 | 15 | 14 | 13 | 30 | 25 | 10 | 11 | 12 | 14 | 9 | 11 | 13 | 17 | 19 | 29 | 16 | 19 | 10 | 26 | 39 | 11 | 12 | 12 | 14 | 17 | 17 | 15 | 15 | 23 | 21 | 8 | 8 | 10 | 2 | 2 | 2 |
| S221 | Han | 37,38 | 14,16 | 11,16 | 19,21 | 16 | 13 | 12 | 30 | 23 | 10 | 11 | 14 | 14 | 10 | 11 | 12 | 27 | 20 | 30 | 15 | 17 | 10 | 24 | 36 | 11 | 12 | 12 | 17 | 16 | 14 | 17 | 15 | 22 | 22 | 9 | 8 | 11 | 2 | 2 | 2 |
| S222 | Han | 37,38 | 15,18 | 14,18 | 22,23 | 12 | 12 | 12 | 28 | 25 | 11 | 13 | 12 | 14 | 10 | 12 | 12 | 24 | 19 | 27 | 14 | 19 | 10 | 25 | 40 | 12 | 10 | 13 | 16 | 19.3 | 17 | 16 | 15 | 22 | 23 | 10 | 8 | 12 | 1 | 2 | 2 |
| S223 | Han | 36,37 | 14,14 | 12,19 | 22,23 | 16 | 12 | 12 | 29 | 24 | 10 | 13 | 12 | 15 | 9 | 12 | 11 | 24 | 20 | 32 | 15 | 17 | 10 | 26 | 38 | 11 | 11 | 13 | 17 | 19 | 17 | 16 | 15 | 18 | 22 | 10 | 8 | 11 | 1 | 2 | 2 |
| S224 | Han | 37,37 | 13,17 | 13,17 | 23,23 | 15 | 10 | 12 | 28 | 24 | 10 | 14 | 12 | 15 | 11 | 13 | 12 | 23 | 20 | 32 | 14 | 16 | 9 | 24 | 36 | 13 | 12 | 12 | 14 | 16 | 21 | 16 | 14 | 18 | 20 | 11 | 8 | 12 | 1 | 1 | 1 |
| S225 | Han | 36,38 | 15,17 | 14,17 | 22,22 | 17 | 12 | 12 | 29 | 25 | 10 | 13 | 12 | 14 | 10 | 11 | 12 | 24 | 18 | 28 | 14 | 19 | 10 | 24 | 38 | 10 | 11 | 13 | 16 | 19 | 16 | 17 | 15 | 20 | 21 | 10 | 8 | 13 | 1 | 2 | 2 |
| S226 | Han | 35,36 | 15,15 | 11,12 | 23,23 | 14 | 12 | 13 | 29 | 24 | 11 | 16 | 13 | 14 | 10 | 11 | 13 | 25 | 19 | 30 | 15 | 16 | 11 | 24 | 40 | 12 | 12 | 12 | 15 | 19 | 17 | 16 | 14 | 18 | 21 | 10 | 8 | 13 | 1 | 2 | 2 |
| S227 | Han | 35,40 | 13,13 | 12,19 | 20,23 | 14 | 10 | 12 | 28 | 23 | 10 | 14 | 13 | 14 | 11 | 11 | 12 | 23 | 20 | 32 | 15 | 19 | 9 | 23 | 37 | 11 | 11 | 13 | 14 | 20 | 18 | 16 | 14 | 18 | 20 | 11 | 8 | 12 | 1 | 1 | 1 |
| S228 | Han | 34,40 | 13,15 | 12,18 | 21,22 | 15 | 10 | 12 | 28 | 24 | 10 | 13 | 12 | 15 | 11 | 12 | 12 | 23 | 20 | 31 | 15 | 16 | 9 | 25 | 38 | 13 | 12 | 12 | 14 | 17 | 16 | 16 | 14 | 22 | 21 | 11 | 9 | 12 | 1 | 1 | 2 |
| S229 | Han | 37,39 | 14,16 | 13,20 | 22,23 | 17 | 12 | 13 | 28 | 23 | 11 | 13 | 12 | 14 | 10 | 12 | 12 | 24 | 19 | 33 | 14 | 18 | 10 | 25 | 41 | 12 | 11 | 13 | 16 | 18 | 17 | 17 | 15 | 19 | 21 | 10 | 8 | 12 | 1 | 2 | 2 |
| S230 | Han | 36,39 | 16,16 | 11,14 | 20,21 | 15 | 13 | 14 | 31 | 23 | 10 | 11 | 15 | 14 | 10 | 11 | 14 | 25 | 21 | 31 | 15 | 16 | 11 | 27 | 39 | 12 | 12 | 12 | 20 | 17 | 18 | 17 | 15 | 20 | 21 | 8 | 6 | 11 | 2 | 2 | 2 |
| S231 | Han | 35,37 | 13,14 | 13,19 | 20,22 | 14 | 10 | 13 | 28 | 24 | 10 | 14 | 12 | 15 | 11 | 13 | 14 | 23 | 21 | 34 | 14 | 17 | 9 | 23 | 36 | 11 | 11 | 13 | 14 | 18 | 20 | 16 | 14 | 18 | 22 | 12 | 8 | 12 | 1 | 1 | 1 |
| S232 | Han | 35,39 | 13,14 | 13,21 | 20,21 | 15 | 10 | 13 | 29 | 25 | 10 | 14 | 12 | 14 | 11 | 11 | 12 | 23 | 20 | 33 | 14 | 19 | 9 | 23 | 36 | 12 | 11 | 12 | 14 | 18 | 18 | 16 | 14 | 18 | 21 | 11 | 8 | 12 | 1 | 1 | 1 |
| S233 | Han | 35,39 | 13,13 | 15,17 | 22,24 | 14 | 10 | 12 | 28 | 24 | 10 | 14 | 12 | 15 | 11 | 12 | 9 | 23 | 19 | 37 | 15 | 18 | 9 | 23 | 37 | 11 | 11 | 12 | 14 | 18 | 18 | 16 | 14 | 20 | 20 | 11 | 8 | 12 | 1 | 1 | 1 |
| S234 | Korean | 37,38 | 13,13 | 11,16 | 23,24 | 13 | 12 | 13 | 29 | 23 | 10 | 12 | 12 | 15 | 10 | 13 | 14 | 24 | 19 | 34 | 15 | 18 | 10 | 24 | 35 | 12 | 11 | 12 | 15 | 16 | 20 | 16 | 14 | 23 | 20 | 11 | 9 | 12 | 1 | 2 | 2 |
| S235 | Korean | 36,36 | 11,11 | 13,16 | 20,20 | 17 | 12 | 14 | 31 | 25 | 10 | 11 | 13 | 14 | 10 | 12 | 12 | 25 | 17 | 27 | 15 | 16 | 10 | 27 | 37 | 12 | 10 | 12 | 16 | 19 | 18 | 15 | 16 | 20 | 21 | 14 | 8 | 11 | 2 | 2 | 2 |
| S236 | Korean | 35,37 | 13,13 | 13,18 | 21,23 | 14 | 10 | 12 | 28 | 24 | 10 | 14 | 12 | 15 | 11 | 12 | 12 | 23 | 20 | 35 | 15 | 18 | 10 | 23 | 36 | 11 | 11 | 11 | 13 | 18 | 20 | 16 | 14 | 18 | 20 | 10 | 8 | 12 | 1 | 1 | 1 |
| S237 | Korean | 36,40 | 14,14 | 12,21 | 21,22 | 16 | 12 | 12 | 28 | 23 | 10 | 13 | 12 | 15 | 10 | 13 | 12 | 24 | 21 | 31 | 15 | 17 | 9 | 24 | 43 | 12 | 11 | 12 | 14 | 21 | 18 | 17 | 15 | 19 | 23 | 14 | 8 | 13 | 1 | 2 | 2 |
| S238 | Korean | 36,38 | 14,15 | 14,17 | 23,25 | 15 | 10 | 12 | 28 | 24 | 10 | 14 | 12 | 15 | 11 | 12 | 13 | 23 | 21 | 33 | 14 | 16 | 9 | 24 | 36 | 13 | 11 | 12 | 14 | 16 | 18 | 16 | 14 | 20 | 20 | 11 | 8 | 12 | 1 | 1 | 1 |
| S239 | Korean | 37,38 | 15,16 | 14,18 | 23,24 | 17 | 12 | 12 | 27 | 25 | 10 | 13 | 12 | 14 | 10 | 12 | 13 | 24 | 19 | 27 | 14 | 18 | 10 | 25 | 38 | 12 | 10 | 13 | 15 | 19.3 | 18 | 16 | 15 | 21 | 22 | 10 | 8 | 13 | 1 | 2 | 2 |
| S240 | Korean | 36,38 | 14,16 | 14,19 | 22,22 | 17 | 12 | 12 | 32 | 25 | 10 | 13 | 12 | 14 | 10 | 11 | 12 | 24 | 20 | 28 | 13 | 20 | 10 | 25 | 38 | 11 | 11 | 13 | 16 | 18 | 17 | 17 | 15 | 21 | 22 | 10 | 8 | 12 | 1 | 2 | 2 |
| S241 | Korean | 37,39 | 12,13 | 15,21 | 20,20 | 17 | 13 | 12 | 29 | 24 | 10 | 13 | 12 | 15 | 10 | 11 | 12 | 24 | 20 | 31 | 15 | 18 | 12 | 23 | 40 | 12 | 11 | 13 | 14 | 21 | 17 | 17 | 15 | 21 | 22 | 11 | 8 | 12 | 1 | 2 | 2 |
| S242 | Korean | 35,39 | 16,17 | 10,17 | 20,20 | 16 | 12 | 14 | 29 | 23 | 10 | 13 | 13 | 14 | 13 | 12 | 14 | 25 | 18 | 31 | 15 | 18 | 11 | 23 | 41 | 11 | 11 | 13 | 15 | 17 | 18 | 16 | 15 | 23 | 22 | 13 | 8 | 11 | 1 | 2 | 2 |
| S243 | Korean | 37,37 | 11,11 | 14,17 | 20,20 | 15 | 12 | 13 | 30 | 25 | 10 | 11 | 13 | 14 | 10 | 12 | 12 | 25 | 17 | 31 | 16 | 16 | 12 | 26 | 39 | 12 | 13 | 11 | 18 | 19 | 19 | 15 | 16 | 23 | 21 | 15 | 8 | 12 | 2 | 2 | 2 |
| S244 | Korean | 35,40 | 12,14 | 12,16 | 20,23 | 15 | 12 | 12 | 28 | 23 | 10 | 12 | 12 | 15 | 10 | 11 | 12 | 24 | 19 | 35 | 15 | 19 | 9 | 22 | 35 | 12 | 11 | 13 | 15 | 16 | 18 | 16 | 14 | 21 | 20 | 10 | 9 | 13 | 1 | 2 | 2 |
| S245 | Korean | 35,41 | 15,16 | 10,18 | 22,23 | 15 | 12 | 13 | 27 | 23 | 10 | 13 | 13 | 14 | 13 | 13 | 12 | 25 | 18 | 28 | 16 | 17 | 10 | 21 | 37 | 12 | 11 | 12 | 14 | 18 | 17 | 16 | 15 | 20 | 20 | 13 | 8 | 12 | 1 | 2 | 2 |
| S246 | Korean | 38,40 | 14,16 | 14,14 | 21,22 | 15 | 12 | 12 | 28 | 23 | 10 | 13 | 13 | 14 | 10 | 12 | 11 | 25 | 18 | 30 | 15 | 18 | 11 | 23 | 36 | 10 | 11 | 12 | 14 | 17 | 18 | 15 | 15 | 20 | 20 | 11 | 8 | 12 | 1 | 2 | 2 |
| S247 | Korean | 36,38 | 15,16 | 12,18 | 22,25 | 15 | 12 | 12 | 28 | 25 | 10 | 13 | 12 | 13 | 10 | 12 | 12 | 24 | 19 | 30 | 15 | 17 | 10 | 27 | 40 | 12 | 11 | 12 | 16 | 17 | 16 | 17 | 15 | 21 | 21 | 10 | 8 | 12 | 1 | 2 | 2 |
| S248 | Korean | 35,35 | 11,11 | 13,19 | 19,19 | 17 | 12 | 13 | 30 | 26 | 10 | 11 | 14 | 14 | 10 | 11 | 13 | 25 | 18 | 30 | 16 | 17 | 10 | 27 | 40 | 12 | 12 | 12 | 17 | 19 | 20 | 15 | 16 | 22 | 23 | 12 | 8 | 12 | 2 | 2 | 2 |
| S249 | Korean | 35,39 | 13,16 | 10,20 | 21,22 | 15 | 12 | 14 | 30 | 22 | 10 | 13 | 13 | 14 | 13 | 12 | 12 | 25 | 18 | 29 | 15 | 19 | 11 | 21 | 38 | 11 | 11 | 11 | 14 | 18 | 16 | 16 | 15 | 21 | 21 | 12 | 8 | 12 | 1 | 2 | 2 |
| S250 | Korean | 36,38 | 14,14 | 12,17 | 21,23 | 15 | 12 | 12 | 29 | 23 | 10 | 12 | 13 | 14 | 10 | 12 | 14 | 25 | 19 | 34 | 17 | 17 | 11 | 21 | 42 | 12 | 11 | 12 | 14 | 16 | 19 | 16 | 14 | 21 | 20 | 11 | 8 | 12 | 1 | 2 | 2 |
| S251 | Korean | 36,39 | 14,14 | 11,19 | 22,24 | 15 | 12 | 12 | 28 | 22 | 10 | 13 | 12 | 14 | 10 | 11 | 12 | 25 | 19 | 31 | 17 | 21 | 9 | 24 | 38 | 12 | 13 | 12 | 14 | 17 | 19 | 16 | 15 | 22 | 21 | 8 | 8 | 12 | 1 | 2 | 2 |
| S252 | Korean | 36,36 | 13,14 | 12,19 | 22,24 | 16 | 12 | 12 | 28 | 25 | 10 | 13 | 12 | 14 | 10 | 12 | 12 | 24 | 19 | 34 | 14 | 17 | 11 | 25 | 41 | 12 | 11 | 12 | 16 | 20 | 20 | 17 | 15 | 20 | 22 | 10 | 8 | 10 | 1 | 2 | 2 |
| S253 | Korean | 37,39 | 13,14 | 13,18 | 20,24 | 14 | 10 | 14 | 30 | 24 | 10 | 14 | 12 | 15 | 11 | 13 | 13 | 23 | 20 | 33 | 15 | 18 | 9 | 23 | 37 | 11 | 11 | 12 | 14 | 18 | 17 | 16 | 14 | 19 | 20 | 11 | 8 | 12 | 1 | 1 | 1 |
| S254 | Korean | 36,38 | 15,15 | 13,20 | 24,24 | 16 | 12 | 12 | 28 | 24 | 10 | 13 | 12 | 15 | 10 | 13 | 13 | 24 | 20 | 32 | 14 | 18 | 10 | 27 | 38 | 12 | 11 | 13 | 14 | 19 | 18 | 18 | 15 | 19 | 21 | 11 | 8 | 11 | 1 | 2 | 2 |
| S255 | Korean | 35,38 | 14,16 | 9,19 | 21,22 | 15 | 12 | 14 | 30 | 22 | 10 | 13 | 13 | 14 | 13 | 12 | 12 | 25 | 17 | 32 | 15 | 19 | 11 | 19 | 38 | 11 | 11 | 12 | 14 | 22 | 19 | 16 | 15 | 20 | 20 | 12 | 8 | 12 | 1 | 2 | 2 |
| S256 | Korean | 36,39 | 14,15 | 11,11 | 21,22 | 16 | 12 | 13 | 28 | 22 | 10 | 14 | 12 | 14 | 10 | 11 | 12 | 26 | 19 | 31 | 16 | 17 | 9 | 23 | 38 | 12 | 11 | 13 | 15 | 18 | 19 | 16 | 15 | 22 | 20 | 10 | 8 | 12 | 1 | 2 | 2 |
| S257 | Korean | 35,40 | 15,16 | 10,18 | 20,21 | 16 | 12 | 14 | 29 | 23 | 10 | 13 | 13 | 14 | 13 | 13 | 13 | 25 | 18 | 30 | 15 | 17 | 11 | 23 | 40 | 11 | 11 | 12 | 14 | 18 | 17 | 16 | 15 | 20 | 21 | 12 | 8 | 11 | 1 | 2 | 2 |
| S258 | Korean | 35,35 | 14,14 | 11,12 | 22,23 | 15 | 12 | 14 | 30 | 23 | 10 | 14 | 13 | 14 | 10 | 10 | 13 | 27 | 18 | 30 | 14 | 15 | 11 | 22 | 39 | 13 | 11 | 12 | 14 | 18 | 17 | 15 | 14 | 19 | 20 | 11 | 8 | 11 | 2 | 2 | 2 |
| S259 | Korean | 38,38 | 14,14 | 10,20 | 19,20 | 15 | 12 | 14 | 30 | 24 | 10 | 11 | 15 | 14 | 10 | 12 | 13 | 27 | 21 | 31 | 15 | 15 | 10 | 25 | 40 | 11 | 11 | 12 | 17 | 15 | 18 | 17 | 15 | 22 | 23 | 9 | 8 | 10 | 2 | 2 | 2 |
| S260 | Korean | 36,36 | 13,13 | 12,18 | 22,23 | 17 | 13 | 12 | 28 | 24 | 10 | 12 | 12 | 14 | 10 | 14 | 12 | 24 | 19 | 30 | 14 | 19 | 11 | 22 | 38 | 13 | 13 | 11 | 16 | 19 | 18 | 16 | 15 | 20 | 24 | 9 | 8 | 12 | 1 | 2 | 2 |
| S261 | Korean | 36,39 | 13,14 | 12,16 | 22,23 | 15 | 12 | 13 | 30 | 23 | 10 | 12 | 12 | 15 | 10 | 12 | 14 | 25 | 19 | 35 | 15 | 18 | 9 | 22 | 36 | 12 | 11 | 14 | 14 | 15 | 19 | 16 | 14 | 21 | 19 | 12 | 8 | 12 | 1 | 2 | 2 |
| S262 | Korean | 35,39 | 14,16 | 10,21 | 21,21 | 15 | 12 | 14 | 30 | 22 | 10 | 13 | 13 | 14 | 13 | 12 | 12 | 25 | 18 | 30 | 15 | 17 | 11 | 21 | 39 | 11 | 11 | 12 | 14 | 18 | 18 | 16 | 15 | 21 | 20 | 12 | 8 | 12 | 1 | 2 | 2 |
| S263 | Korean | 37,38 | 15,15 | 12,18 | 22,24 | 16 | 12 | 13 | 29 | 24 | 10 | 13 | 12 | 14 | 10 | 13 | 12 | 24 | 20 | 33 | 15 | 17 | 11 | 26 | 35 | 13 | 12 | 13 | 17 | 19 | 18 | 16 | 15 | 22 | 21 | 11 | 8 | 12 | 1 | 2 | 2 |
| S264 | Korean | 35,37 | 9,17 | 12,18 | 19,20 | 15 | 13 | 13 | 29 | 23 | 10 | 11 | 14 | 14 | 10 | 13 | 12 | 28 | 22 | 30 | 15 | 18 | 11 | 24 | 35 | 11 | 12 | 14 | 17 | 16 | 18 | 17 | 14 | 23 | 21 | 9 | 8 | 11 | 2 | 2 | 2 |
| S265 | Korean | 37,37 | 16,16 | 12,18 | 19,19 | 15 | 13 | 13 | 29 | 23 | 10 | 11 | 14 | 14 | 10 | 12 | 12 | 29 | 22 | 30 | 15 | 19 | 11 | 24 | 36 | 11 | 12 | 14 | 16 | 16 | 19 | 17 | 14 | 22 | 21 | 9 | 8 | 11 | 2 | 2 | 2 |
| S266 | Korean | 35,36 | 12,15 | 11,19 | 22,22 | 16 | 12 | 12 | 28 | 26 | 11 | 13 | 12 | 14 | 10 | 13 | 14 | 24 | 19 | 32 | 14 | 20 | 10 | 23 | 41 | 14 | 11 | 12 | 16 | 18 | 19 | 16 | 15 | 20 | 23 | 10 | 8 | 11 | 1 | 2 | 2 |
| S267 | Korean | 35,39 | 13,14 | 11,17 | 20,24 | 15 | 12 | 13 | 30 | 23 | 10 | 12 | 12 | 14 | 10 | 12 | 14 | 25 | 19 | 33 | 14 | 18 | 9 | 22 | 37 | 12 | 11 | 12 | 16 | 16 | 18 | 16 | 14 | 23 | 19 | 12 | 8 | 12 | 1 | 2 | 2 |
| S268 | Korean | 35,40 | 15,16 | 10,17 | 20,21 | 16 | 12 | 14 | 29 | 23 | 10 | 13 | 13 | 14 | 13 | 12 | 13 | 26 | 18 | 30 | 16 | 17 | 11 | 24 | 40 | 11 | 11 | 12 | 14 | 19 | 17 | 16 | 15 | 20 | 22 | 13 | 8 | 11 | 1 | 2 | 2 |
| S269 | Korean | 36,41 | 14,16 | 10,10 | 22,22 | 15 | 13 | 14 | 29 | 23 | 10 | 14 | 13 | 14 | 12 | 12 | 13 | 24 | 19 | 31.2 | 15 | 17 | 11 | 21 | 39 | 12 | 10 | 12 | 14 | 18 | 19 | 16 | 15 | 15 | 20 | 12 | 8 | 12 | 1 | 2 | 2 |
| S270 | Korean | 37,40 | 13,14 | 12,16 | 20,23 | 15 | 12 | 12 | 29 | 23 | 11 | 12 | 12 | 14 | 10 | 13 | 14 | 25 | 19 | 34 | 15 | 18 | 10 | 21 | 39 | 12 | 11 | 12 | 14 | 17 | 20 | 16 | 14 | 22 | 19 | 11 | 8 | 13 | 1 | 2 | 2 |
| S271 | Korean | 35,38 | 9,16 | 12,18 | 19,20 | 16 | 13 | 13 | 29 | 23 | 10 | 11 | 14 | 14 | 10 | 12 | 12 | 28 | 22 | 31 | 15 | 18 | 11 | 24 | 36 | 12 | 12 | 13 | 17 | 16 | 17 | 17 | 15 | 23 | 22 | 9 | 8 | 11 | 2 | 2 | 2 |
| S272 | Korean | 35,40 | 15,16 | 10,18 | 20,21 | 16 | 12 | 13 | 28 | 23 | 10 | 13 | 13 | 14 | 13 | 12 | 13 | 25 | 18 | 30 | 15 | 18 | 11 | 23 | 40 | 11 | 11 | 12 | 14 | 18 | 17 | 16 | 15 | 20 | 21 | 13 | 8 | 11 | 1 | 2 | 2 |
| S273 | Korean | 36,36 | 13,13 | 13,14,18 | 22,22 | 15 | 12 | 14 | 30 | 24 | 11 | 13 | 14 | 13 | 10 | 12 | 12 | 27 | 18 | 26 | 14 | 19 | 10 | 23 | 41 | 12 | 10 | 11 | 14 | 18 | 21 | 15 | 15 | 22 | 22 | 10 | 8 | 10 | 1 | 2 | 2 |
| S274 | Korean | 37,37 | 14,17 | 11,19 | 19,20 | 15 | 12 | 14 | 28 | 23 | 11 | 11 | 14 | 14 | 10 | 12 | 13 | 28 | 21 | 29 | 15 | 16 | 11 | 24 | 35 | 11 | 12 | 12 | 17 | 17 | 17 | 17 | 15 | 22 | 21 | 9 | 8 | 10 | 1 | 2 | 2 |
| S275 | Korean | 35,39 | 15,18 | 10,20 | 21,22 | 16 | 12 | 14 | 30 | 23 | 10 | 14 | 12 | 14 | 13 | 13 | 14 | 24 | 19 | 32 | 15 | 18 | 11 | 23 | 40 | 11 | 10 | 12 | 14 | 21 | 18 | 16 | 15 | 17 | 20 | 11 | 8 | 12 | 1 | 2 | 2 |
| S276 | Korean | 37,38 | 12,13 | 15,21 | 20,21 | 17 | 13 | 12 | 29 | 24 | 10 | 13 | 12 | 15 | 10 | 11 | 13 | 24 | 20 | 31 | 15 | 17 | 11 | 23 | 40 | 13 | 11 | 14 | 14 | 19 | 18 | 17 | 15 | 20 | 21 | 11 | 8 | 12 | 1 | 2 | 2 |
| S277 | Korean | 36,36 | 12,12 | 15,20 | 20,20 | 17 | 13 | 12 | 30 | 24 | 10 | 13 | 12 | 15 | 10 | 11 | 13 | 24 | 20 | 29 | 15 | 18 | 12 | 23 | 39 | 12 | 11 | 13 | 14 | 18 | 19 | 17 | 15 | 21 | 22 | 11 | 8 | 13 | 1 | 2 | 2 |
| S278 | Korean | 36,38 | 14,16 | 12,18 | 23,23 | 16 | 12 | 12 | 27 | 24 | 11 | 13 | 13 | 14 | 10 | 12 | 12 | 24 | 20 | 27 | 14 | 18 | 10 | 26 | 38 | 12 | 11 | 13 | 16 | 18 | 17 | 16 | 15 | 19 | 22 | 9 | 8 | 11 | 1 | 2 | 2 |
| S279 | Korean | 37,38 | 12,13 | 15,20 | 20,20 | 17 | 13 | 12 | 30 | 24 | 10 | 13 | 12 | 15 | 10 | 12 | 12 | 24 | 20 | 31 | 15 | 17 | 12 | 23 | 39 | 12 | 12 | 12 | 14 | 21 | 17 | 17 | 15 | 20 | 22 | 11 | 8 | 11 | 1 | 2 | 2 |
| S280 | Korean | 36,36 | 15,15 | 11,11 | 23,23 | 14 | 12 | 13 | 30 | 21 | 10 | 15 | 13 | 14 | 10 | 11 | 13 | 26 | 19 | 29 | 16 | 17 | 13 | 23 | 38 | 13 | 11 | 13 | 14 | 21 | 20 | 15 | 14 | 23 | 21 | 10 | 8 | 12 | 1 | 2 | 2 |
| S281 | Korean | 36,39 | 14,15 | 13,19 | 21,22 | 17 | 13 | 12 | 28 | 24 | 9 | 13 | 12 | 15 | 10 | 11 | 14 | 23 | 21 | 31 | 16 | 17 | 9 | 23 | 40 | 11 | 11 | 12 | 15 | 16 | 17 | 16 | 15 | 19 | 22 | 11 | 8 | 13 | 1 | 2 | 2 |
| S282 | Korean | 37,39 | 14,15 | 12,21 | 22,23 | 16 | 12 | 12 | 28 | 25 | 10 | 13 | 13 | 14 | 10 | 12 | 12 | 25 | 19 | 29 | 14 | 19 | 10 | 24 | 36 | 12 | 10 | 13 | 16 | 16 | 15 | 16 | 15 | 20 | 21 | 11 | 8 | 11 | 1 | 2 | 2 |
| S283 | Korean | 35,39 | 14,17 | 10,20 | 21,21 | 15 | 12 | 14 | 29 | 22 | 10 | 13 | 13 | 14 | 13 | 12 | 12 | 25 | 18 | 30 | 15 | 18 | 11 | 21 | 38 | 11 | 12 | 13 | 14 | 18 | 22 | 16 | 15 | 17 | 20 | 12 | 8 | 12 | 1 | 2 | 2 |
| S284 | Korean | 35,36,37 | 13,14,15 | 11,18 | 19,20 | 16 | 13 | 13 | 29 | 23 | 10 | 11 | 15 | 14 | 10 | 10 | 12 | 27 | 21 | 29 | 15 | 16 | 11 | 23 | 36 | 11 | 12 | 12 | 19.1 | 16 | 19 | 17 | 15 | 20 | 20 | 10 | 8 | 12 | 2 | 2 | 2 |
| S285 | Korean | 35,39 | 16,17 | 10,16 | 22,24 | 15 | 12 | 12 | 27 | 23 | 10 | 13 | 13 | 14 | 13 | 12 | 13 | 25 | 18 | 28 | 15 | 17 | 10 | 21 | 36 | 9 | 11 | 12 | 14 | 19 | 18 | 16 | 15 | 20 | 20 | 12 | 8 | 12 | 1 | 2 | 2 |
| S286 | Korean | 38,39 | 14,15 | 21,22 | 21,25 | 13 | 12 | 13 | 29 | 25 | 10 | 15 | 13 | 15 | 11 | 12 | 13 | 28 | 19 | 31 | 15 | 19 | 10 | 24 | 40.2 | 10 | 12 | 12 | 17 | 18 | 18 | 15 | 16 | 21 | 22 | 10 | 8 | 10 | 1 | 2 | 2 |
| S287 | Korean | 34,40 | 15,16 | 10,18 | 22,22 | 15 | 12 | 13 | 29 | 22 | 10 | 13 | 13 | 14 | 13 | 12 | 12 | 25 | 18 | 29 | 15 | 18 | 11 | 21 | 38 | 11 | 11 | 12 | 14 | 18 | 18 | 16 | 15 | 21 | 20 | 12 | 8 | 11 | 1 | 2 | 2 |
| S288 | Korean | 36,39 | 12,13 | 14,22 | 20,21 | 15 | 13 | 12 | 28 | 24 | 10 | 13 | 12 | 14 | 10 | 11 | 15 | 24 | 20 | 33 | 16 | 16 | 11 | 22 | 39 | 12 | 11 | 13 | 14 | 19 | 20 | 17 | 15 | 20 | 22 | 11 | 8 | 12 | 1 | 2 | 2 |
| S289 | Korean | 37,39 | 14,15 | 11,17 | 18,21 | 15 | 12 | 15 | 30 | 23 | 10 | 11 | 15 | 14 | 10 | 13 | 13 | 26 | 21 | 29 | 15 | 16 | 10 | 25 | 38 | 11 | 12 | 12 | 16 | 17 | 18 | 17 | 15 | 21 | 21 | 9 | 8 | 11 | 2 | 2 | 2 |
| S290 | Korean | 36,38 | 13,14 | 12,19 | 22,23 | 16 | 12 | 12 | 29 | 27 | 10 | 13 | 12 | 14 | 10 | 12 | 13 | 24 | 20 | 32 | 14 | 17 | 10 | 23 | 38 | 13 | 12 | 12 | 16 | 18 | 16 | 16 | 15 | 20 | 21 | 11 | 8 | 12 | 1 | 2 | 2 |
| S291 | Korean | 37,37 | 14,14 | 11,17 | 20,21 | 16 | 13 | 13 | 32 | 24 | 10 | 11 | 14 | 14 | 10 | 12 | 13 | 24 | 21 | 31 | 15 | 15 | 11 | 23 | 38 | 12 | 12 | 12 | 17 | 17 | 16 | 17 | 15 | 22 | 21 | 9 | 8 | 11 | 2 | 2 | 2 |
| S292 | Korean | 36,39 | 12,16 | 12,17 | 21,22 | 17 | 12 | 12 | 28 | 24 | 10 | 13 | 12 | 14 | 10 | 13 | 13 | 24 | 19 | 32 | 15 | 18 | 10 | 24 | 40 | 12 | 11 | 13 | 16 | 17 | 17 | 15 | 15 | 20 | 21 | 10 | 8 | 12 | 1 | 2 | 2 |
| S293 | Korean | 37,38 | 12,13 | 16,19 | 20,21 | 17 | 13 | 12 | 30 | 24 | 10 | 13 | 12 | 15 | 10 | 12 | 13 | 24 | 20 | 29 | 15 | 16 | 11 | 23 | 37 | 13 | 12 | 13 | 14 | 19 | 17 | 17 | 15 | 20 | 21 | 11 | 8 | 12 | 1 | 2 | 2 |
| S294 | Korean | 35,40 | 15,16 | 10,19 | 21,22 | 16 | 12 | 14 | 29 | 23 | 10 | 13 | 13 | 13 | 13 | 12 | 14 | 25 | 18 | 31 | 16 | 17 | 11 | 23 | 41 | 11 | 11 | 12 | 14 | 18 | 17 | 16 | 15 | 21 | 21 | 13 | 8 | 11 | 1 | 2 | 2 |
| S295 | Korean | 38,39 | 13,14 | 11,11 | 22,24 | 15 | 12 | 12 | 29 | 23 | 10 | 12 | 12 | 15 | 10 | 10 | 12 | 24 | 19 | 32 | 15 | 18 | 11 | 20 | 37 | 12 | 11 | 14 | 15 | 18 | 18 | 16 | 14 | 24 | 21 | 11 | 8 | 11 | 1 | 2 | 2 |
| S296 | Korean | 36,38 | 14,17 | 13,19 | 22,22 | 17 | 12 | 13 | 28 | 24 | 10 | 13 | 12 | 14 | 10 | 12 | 12 | 24 | 19 | 33 | 15 | 20 | 10 | 25 | 38 | 12 | 11 | 12 | 18 | 20 | 18 | 16 | 15 | 23 | 24 | 10 | 8 | 13 | 1 | 2 | 2 |
| S297 | Korean | 35,39 | 14,16 | 10,21 | 21,21 | 15 | 12 | 13 | 29 | 22 | 10 | 13 | 13 | 14 | 13 | 11 | 10 | 25 | 18 | 28 | 15 | 18 | 11 | 21 | 39 | 11 | 11 | 12 | 14 | 20 | 20 | 16 | 15 | 19 | 20 | 11 | 8 | 12 | 1 | 2 | 2 |
| S298 | Korean | 36,38 | 15,16 | 12,20 | 23,23 | 16 | 12 | 12 | 28 | 25 | 10 | 13 | 12 | 14 | 10 | 12 | 13 | 24 | 19 | 30 | 16 | 17 | 10 | 27 | 38 | 12 | 11 | 12 | 16 | 16 | 18 | 16 | 15 | 21 | 21 | 10 | 8 | 11 | 1 | 2 | 2 |
| S299 | Korean | 35,39 | 15,15 | 10,18 | 19,22 | 17 | 12 | 14 | 30 | 23 | 11 | 13 | 13 | 14 | 12 | 12 | 14 | 24 | 18 | 28 | 14 | 16 | 11 | 23 | 39 | 11 | 11 | 12 | 14 | 21 | 20 | 16 | 15 | 18 | 21 | 12 | 8 | 12 | 1 | 2 | 2 |
| S300 | Korean | 36,40 | 14,17 | 10,18 | 20,21 | 15 | 12 | 13 | 30 | 22 | 10 | 13 | 13 | 14 | 13 | 11 | 12 | 25 | 18 | 30 | 15 | 19 | 11 | 21 | 39 | 11 | 11 | 12 | 14 | 19 | 20 | 16 | 15 | 21 | 20 | 12 | 8 | 11 | 1 | 2 | 2 |
| S301 | Korean | 39,41 | 14,17 | 12,17 | 20,24 | 15 | 12 | 12 | 29 | 23 | 11 | 13 | 13 | 15 | 10 | 12 | 13 | 23 | 19 | 30 | 13 | 17 | 9 | 26 | 39 | 11 | 12 | 11 | 15 | 15 | 17 | 16 | 16 | 21 | 22 | 12 | 8 | 11 | 1 | 2 | 2 |
| S302 | Korean | 39,39 | 15,15 | 16,21 | 21,21 | 14 | 12 | 13 | 30 | 24 | 9 | 14 | 14 | 14 | 12 | 11 | 14 | 26 | 19 | 29 | 15 | 17 | 11 | 24 | 39.2 | 10 | 12 | 13 | 19 | 19 | 19 | 15 | 16 | 25 | 22 | 10 | 8 | 10 | 2 | 2 | 2 |
| S303 | Korean | 34,36 | 11,11 | 9,16 | 20,22 | 16 | 12 | 14 | 30 | 25 | 10 | 11 | 13 | 14 | 10 | 12 | 13 | 25 | 17 | 33 | 18 | 17 | 11 | 27 | 40 | 12 | 12 | 12 | 17 | 17 | 18 | 15 | 16 | 22 | 25 | 11 | 8 | 11 | 2 | 2 | 2 |
| S304 | Korean | 35,37 | 17,17 | 12,18 | 19,20 | 16 | 13 | 13 | 29 | 23 | 10 | 11 | 14 | 14 | 10 | 12 | 13 | 28 | 22 | 30 | 15 | 18 | 11 | 24 | 36 | 11 | 12 | 14 | 17 | 16 | 20 | 17 | 15 | 22 | 21 | 10 | 8 | 11 | 2 | 2 | 2 |
| S305 | Korean | 34,39 | 14,17 | 10,19 | 20,21 | 15 | 12 | 13 | 29 | 22 | 10 | 13 | 13 | 14 | 13 | 12 | 13 | 25 | 18 | 31 | 15 | 18 | 11 | 21 | 38 | 11 | 11 | 12 | 15 | 20 | 20 | 16 | 15 | 20 | 20 | 12 | 8 | 12 | 1 | 2 | 2 |
| S306 | Korean | 35,35 | 11,11 | 13,18 | 20,20 | 17 | 12 | 13 | 30 | 26 | 10 | 11 | 13 | 14 | 10 | 11 | 11.2 | 25 | 18 | 30 | 15 | 16 | 10 | 28 | 35 | 13.3 | 12 | 12 | 18 | 17 | 18 | 15 | 16 | 23 | 22 | 12 | 8 | 12 | 2 | 2 | 2 |
| S307 | Korean | 36,37 | 13,14 | 12,18 | 20,23 | 14 | 10 | 12 | 28 | 24 | 10 | 13 | 12 | 14 | 11 | 12 | 12 | 24 | 22 | 32 | 15 | 16 | 9 | 24 | 35 | 11 | 11 | 12 | 14 | 20 | 18 | 16 | 14 | 18 | 21 | 11 | 8 | 11 | 1 | 1 | 1 |
| S308 | Korean | 35,39 | 15,16 | 17,20 | 21,23 | 15 | 12 | 13 | 30 | 23 | 10 | 13 | 13 | 14 | 13 | 11 | 14 | 24 | 17 | 25 | 14 | 17 | 11 | 22 | 37 | 11 | 11 | 12 | 13 | 18 | 20 | 16 | 15 | 22 | 20 | 13 | 8 | 12 | 1 | 2 | 2 |
| S309 | Korean | 36,38 | 14,15 | 14,17 | 23,25 | 15 | 10 | 12 | 28 | 24 | 10 | 14 | 12 | 15 | 11 | 11 | 12 | 23 | 21 | 32 | 14 | 16 | 9 | 24 | 36 | 13 | 11 | 12 | 14 | 16 | 19 | 16 | 14 | 20 | 21 | 11 | 8 | 12 | 1 | 1 | 1 |
| S310 | Korean | 36,40 | 13,14 | 12,12 | 21,22 | 13 | 12 | 12 | 27 | 25 | 10 | 13 | 13 | 15 | 10 | 12 | 12 | 24 | 19 | 32 | 15 | 18 | 9 | 22 | 38 | 12 | 11 | 13 | 16 | 18 | 18 | 16 | 15 | 19 | 22 | 9 | 8 | 12 | 1 | 2 | 2 |
| S311 | Korean | 35,38 | 12,13 | 13,18 | 20,24 | 14 | 10 | 12 | 27 | 24 | 10 | 14 | 12 | 15 | 11 | 11 | 14 | 23 | 20 | 34 | 15 | 17 | 10 | 24 | 35 | 11 | 11 | 13 | 14 | 18 | 17 | 16 | 14 | 16 | 21 | 10 | 8 | 12 | 1 | 1 | 1 |
| S312 | Korean | 39,39 | 15,15 | 17,17 | 22,22 | 14 | 12 | 14 | 30 | 24 | 9 | 14 | 14 | 14 | 12 | 12 | 13 | 27 | 19 | 30 | 15 | 15 | 10 | 23 | 37.2 | 10 | 12 | 12 | 20 | 19 | 17 | 15 | 16 | 23 | 22 | 10 | 8 | 10 | 2 | 2 | 2 |
| S313 | Korean | 38,38 | 12,13 | 15,21 | 20,21 | 16 | 13 | 12 | 30 | 24 | 10 | 13 | 12 | 15 | 10 | 11 | 12 | 24 | 20 | 32 | 15 | 16 | 11 | 24 | 41 | 12 | 11 | 13 | 14 | 19 | 22 | 17 | 15 | 21 | 21 | 11 | 8 | 12 | 1 | 2 | 2 |
| S314 | Korean | 36,38 | 14,14 | 14,17 | 22,23 | 16 | 12 | 12 | 28 | 23 | 10 | 13 | 12 | 14 | 10 | 11 | 12 | 24 | 20 | 31 | 14 | 16 | 10 | 23 | 40 | 12 | 11 | 13 | 15 | 19 | 16 | 16 | 15 | 22 | 21 | 10 | 8 | 12 | 1 | 2 | 2 |
| S315 | Korean | 35,35 | 14,15 | 10,12 | 22,24 | 14 | 12 | 14 | 31 | 24 | 11 | 14 | 13 | 14 | 10 | 10 | 14 | 24 | 18 | 29 | 14 | 17 | 11 | 22 | 37 | 12 | 12 | 12 | 15 | 18 | 18 | 15 | 14 | 21 | 20 | 11 | 8 | 12 | 2 | 2 | 2 |
| S316 | Korean | 37,39 | 12,13 | 12,16 | 21,24 | 14 | 13 | 12 | 29 | 23 | 11 | 12 | 12 | 15 | 10 | 14 | 13 | 24 | 19 | 34 | 17 | 19 | 10 | 22 | 36 | 12 | 11 | 13 | 16 | 18 | 19 | 16 | 14 | 21 | 20 | 11 | 8 | 12 | 1 | 2 | 2 |
| S317 | Korean | 38,39 | 12,15 | 12,17 | 21,23 | 15 | 12 | 12 | 30 | 23 | 11 | 12 | 12 | 15 | 10 | 12 | 13 | 23 | 19 | 34 | 17 | 17 | 10 | 22 | 35 | 12 | 11 | 12 | 15 | 16 | 19 | 16 | 14 | 22 | 19 | 10 | 8 | 12 | 1 | 2 | 2 |
| S318 | Korean | 34,38 | 13,14 | 13,19 | 20,24 | 14 | 10 | 12 | 28 | 24 | 10 | 14 | 12 | 15 | 11 | 12 | 11 | 23 | 20 | 32 | 15 | 19 | 9 | 23 | 36 | 11 | 11 | 12 | 14 | 18 | 18 | 16 | 14 | 22 | 22 | 11 | 8 | 13 | 1 | 1 | 1 |
| S319 | Korean | 37,37 | 13,13 | 13,21 | 21,21 | 15 | 12 | 12 | 30 | 25 | 10 | 13 | 12 | 15 | 10 | 11 | 14 | 23 | 20 | 31 | 14 | 16 | 10 | 24 | 39 | 12 | 11 | 13 | 14 | 18 | 17 | 17 | 15 | 20 | 21 | 10 | 8 | 13 | 1 | 2 | 2 |
| S320 | Korean | 36,36 | 14,14 | 11,13 | 23,23 | 16 | 13 | 14 | 30 | 23 | 10 | 14 | 13 | 14 | 10 | 12 | 12 | 26 | 19 | 29 | 15 | 15 | 11 | 22 | 38 | 11 | 12 | 13 | 14 | 18 | 17 | 15 | 14 | 20 | 23 | 10 | 8 | 12 | 2 | 2 | 2 |
| S321 | Korean | 36,40 | 15,16 | 10,20 | 20,21 | 16 | 12 | 13 | 28 | 23 | 10 | 14 | 13 | 14 | 13 | 12 | 13 | 25 | 18 | 29 | 15 | 18 | 11 | 23 | 41 | 11 | 11 | 13 | 15 | 18 | 17 | 16 | 15 | 21 | 21 | 14 | 8 | 11 | 1 | 2 | 2 |
| S322 | Korean | 37,39 | 12,14 | 14,20 | 20,21 | 16 | 13 | 12 | 29 | 25 | 10 | 13 | 12 | 14 | 11 | 11 | 13 | 25 | 20 | 31 | 15 | 18 | 11 | 24 | 39 | 12 | 11 | 14 | 14 | 18 | 19 | 17 | 15 | 20 | 21 | 11 | 8 | 13 | 1 | 2 | 2 |
| S323 | Korean | 36,39 | 13,15 | 13,17 | 23,24 | 17 | 12 | 13 | 28 | 24 | 10 | 12 | 15 | 14 | 10 | 11 | 11 | 26 | 18 | 27 | 15 | 18 | 10 | 23 | 42 | 12 | 10 | 12 | 14 | 16 | 20 | 15 | 16 | 19 | 24 | 12 | 8 | 10 | 1 | 2 | 2 |
| S324 | Korean | 35,39 | 14,17 | 10,19 | 21,21 | 15 | 12 | 14 | 30 | 22 | 10 | 13 | 13 | 14 | 13 | 12 | 12 | 25 | 18 | 30 | 15 | 17 | 11 | 22 | 38 | 11 | 11 | 12 | 14 | 19 | 21 | 16 | 15 | 20 | 20 | 12 | 8 | 12 | 1 | 2 | 2 |
| S325 | Korean | 35,39 | 14,16 | 10,19 | 21,22 | 15 | 12 | 13 | 28 | 22 | 10 | 13 | 14 | 15 | 13 | 12 | 11 | 25 | 18 | 29 | 15 | 19 | 10 | 21 | 39 | 11 | 11 | 12 | 14 | 20 | 18 | 16 | 15 | 21 | 20 | 12 | 9 | 11 | 1 | 2 | 2 |
| S326 | Korean | 36,39 | 13,16 | 12,19 | 22,24 | 16 | 12 | 12 | 29 | 24 | 10 | 13 | 12 | 14 | 10 | 13 | 13 | 24 | 19 | 32 | 14 | 16 | 10 | 24 | 40 | 11 | 12 | 13 | 16 | 17 | 16 | 16 | 15 | 19 | 23 | 10 | 8 | 12 | 1 | 2 | 2 |
| S327 | Korean | 36,39 | 13,14 | 13,16 | 21,24 | 15 | 12 | 12 | 29 | 23 | 11 | 12 | 12 | 15 | 10 | 12 | 13 | 25 | 19 | 34 | 15 | 19 | 9 | 23 | 36 | 12 | 11 | 12 | 12 | 16 | 20 | 16 | 14 | 23 | 19 | 11 | 8 | 12 | 1 | 2 | 2 |
| S328 | Korean | 35,39 | 16,16 | 10,18 | 20,21 | 16 | 12 | 14 | 29 | 23 | 10 | 13 | 13 | 14 | 13 | 12 | 14 | 25 | 18 | 31 | 15 | 18 | 11 | 23 | 40 | 13 | 11 | 12 | 14 | 18 | 17 | 16 | 15 | 22 | 22 | 12 | 8 | 11 | 1 | 2 | 2 |
| S329 | Korean | 35,37 | 9,17 | 12,18 | 19,20 | 16 | 13 | 13 | 29 | 23 | 10 | 11 | 14 | 14 | 10 | 13 | 12 | 28 | 22 | 30 | 15 | 18 | 11 | 24 | 35 | 11 | 12 | 14 | 17 | 16 | 18 | 17 | 14 | 23 | 21 | 9 | 8 | 11 | 2 | 2 | 2 |
| S330 | Korean | 35,39 | 15,15 | 10,18 | 20,21 | 16 | 12 | 14 | 29 | 23 | 10 | 13 | 13 | 14 | 13 | 12 | 14 | 26 | 18 | 31 | 15 | 17 | 11 | 22 | 40 | 11 | 11 | 12 | 14 | 18 | 19 | 16 | 15 | 20 | 21 | 13 | 8 | 12 | 1 | 2 | 2 |
| S331 | Korean | 36,40 | 14,16 | 10,18 | 20,21 | 16 | 12 | 13 | 30 | 22 | 10 | 13 | 13 | 14 | 13 | 12 | 12 | 25 | 18 | 30 | 15 | 19 | 11 | 21 | 39 | 11 | 11 | 12 | 14 | 19 | 19 | 16 | 15 | 21 | 20 | 12 | 8 | 11 | 1 | 2 | 2 |
| S332 | Korean | 35,37 | 13,13 | 14,19 | 20,27 | 14 | 10 | 11 | 27 | 23 | 9 | 14 | 12 | 15 | 11 | 12 | 13 | 23 | 19 | 33 | 15 | 18 | 9 | 24 | 35 | 11 | 11 | 14 | 14 | 19 | 17 | 16 | 14 | 20 | 20 | 12 | 9 | 12 | 1 | 1 | 1 |
| S333 | Korean | 37,39 | 11,14 | 14,21 | 20,21 | 16 | 13 | 12 | 28 | 27 | 10 | 13 | 13 | 14 | 11 | 11 | 13 | 24 | 20 | 32 | 15 | 17 | 11 | 24 | 40 | 12 | 10 | 13 | 14 | 19 | 19 | 17 | 15 | 20 | 21 | 11 | 8 | 12 | 1 | 2 | 2 |
| S334 | Korean | 40,40 | 15,15 | 15,23 | 21,21 | 14 | 12 | 14 | 30 | 24 | 9 | 15 | 14 | 14 | 12 | 11 | 14 | 27 | 19 | 29 | 15 | 17 | 10 | 23 | 38.2 | 10 | 12 | 13 | 19 | 20 | 18 | 15 | 16 | 22 | 22 | 10 | 8 | 10 | 2 | 2 | 2 |
| S335 | Korean | 36,36 | 15,16 | 11,11 | 19,20 | 15 | 13 | 13 | 30 | 23 | 10 | 11 | 15 | 14 | 10 | 11 | 14 | 25 | 21 | 30 | 15 | 15 | 11 | 27 | 38 | 11 | 11 | 12 | 19 | 16 | 20 | 17 | 15 | 24 | 22 | 8 | 8 | 11 | 2 | 2 | 2 |
| S336 | Korean | 35,38 | 14,15 | 10,17 | 21,24 | 15 | 12 | 13 | 29 | 23 | 10 | 13 | 14 | 14 | 10 | 14 | 13 | 29 | 17 | 30 | 15 | 16 | 13 | 23 | 37 | 12 | 11 | 11 | 15 | 17 | 19 | 15 | 15 | 18 | 20 | 11 | 8 | 11 | 1 | 2 | 2 |
| S337 | Korean | 38,40 | 13,14 | 11,19 | 22,24 | 15 | 12 | 12 | 29 | 23 | 10 | 12 | 12 | 15 | 10 | 11 | 12 | 24 | 19 | 32 | 16 | 18 | 11 | 20 | 37 | 13 | 11 | 14 | 15 | 18 | 19 | 16 | 14 | 23 | 21 | 11 | 8 | 11 | 1 | 2 | 2 |
| S338 | Korean | 37,37 | 17,17 | 11,18 | 21,21 | 15 | 13 | 14 | 31 | 23 | 10 | 11 | 13 | 14 | 10 | 11 | 14 | 26 | 22 | 31 | 15 | 17 | 11 | 26 | 37 | 11 | 12 | 12 | 18 | 20 | 18 | 17 | 15 | 23 | 21 | 9 | 8 | 11 | 2 | 2 | 2 |
| S339 | Korean | 36,41 | 14,15 | 11,11 | 20,22 | 16 | 12 | 13 | 28 | 22 | 10 | 14 | 12 | 15 | 10 | 12 | 12 | 26 | 18 | 32 | 16 | 17 | 9 | 23 | 39 | 12 | 11 | 14 | 15 | 18 | 18 | 16 | 15 | 21 | 20 | 10 | 8 | 12 | 1 | 2 | 2 |
| S340 | Korean | 37,39 | 12,13 | 12,16 | 21,24 | 14 | 12 | 12 | 28 | 23 | 10 | 12 | 11 | 15 | 10 | 12 | 12 | 24 | 19 | 32 | 15 | 19 | 9 | 22 | 36 | 12 | 11 | 13 | 16 | 16 | 19 | 16 | 14 | 23 | 20 | 10 | 9 | 13 | 1 | 2 | 2 |
| S341 | Korean | 36,39 | 13,13 | 13,21 | 22,22 | 17 | 12 | 12 | 29 | 26 | 11 | 13 | 12 | 14 | 10 | 11 | 13 | 24 | 18 | 30 | 14 | 19 | 10 | 24 | 36 | 12 | 11 | 11 | 16 | 20 | 18 | 16 | 15 | 20 | 22 | 11 | 8 | 12 | 1 | 2 | 2 |
| S342 | Korean | 38,41 | 13,13 | 12,17 | 21,23 | 15 | 12 | 13 | 30 | 24 | 10 | 13 | 14 | 14 | 10 | 13 | 15 | 27 | 18 | 29 | 15 | 15 | 12 | 23 | 38 | 11 | 11 | 12 | 17 | 19 | 19 | 15 | 15 | 20 | 21 | 12 | 8 | 12 | 1 | 2 | 2 |
| S343 | Korean | 35,37 | 13,15 | 13,19 | 21,24 | 14 | 10 | 13 | 28 | 25 | 10 | 14 | 12 | 15 | 11 | 13 | 12 | 23 | 19 | 32 | 16 | 16 | 9 | 22 | 33 | 11 | 11 | 12 | 14 | 19 | 18 | 17 | 14 | 18 | 20 | 11 | 8 | 12 | 1 | 1 | 1 |
| S344 | Korean | 36,38 | 13,15 | 14,17 | 22,22 | 15 | 12 | 12 | 29 | 24 | 10 | 13 | 12 | 14 | 10 | 11 | 14 | 25 | 20 | 28 | 15 | 16 | 10 | 23 | 41 | 13 | 11 | 13 | 17 | 20 | 19 | 16 | 15 | 22 | 22 | 10 | 8 | 12 | 1 | 2 | 2 |
| S345 | Korean | 36,39 | 13,17 | 9,21 | 21,22 | 16 | 14 | 14 | 30 | 24 | 11 | 13 | 13 | 14 | 12 | 12 | 14 | 24 | 18 | 30 | 15 | 17 | 11 | 23 | 38 | 11 | 11 | 12 | 14 | 22 | 18 | 16 | 15 | 21 | 20 | 12 | 8 | 12 | 1 | 2 | 2 |
| S346 | Korean | 36,39 | 13,16 | 10,20 | 20,21 | 15 | 12 | 14 | 30 | 22 | 10 | 13 | 13 | 14 | 13 | 12 | 12 | 25 | 18 | 31 | 15 | 18 | 11 | 21 | 38 | 11 | 11 | 12 | 14 | 19 | 17 | 16 | 15 | 20 | 20 | 12 | 8 | 12 | 1 | 2 | 2 |
| S347 | Korean | 35,41 | 15,16 | 10,17 | 22,22 | 15 | 12 | 13 | 27 | 23 | 10 | 13 | 13 | 14 | 13 | 12 | 12 | 25 | 18 | 28 | 15 | 18 | 10 | 22 | 37 | 12 | 11 | 13 | 14 | 18 | 19 | 16 | 15 | 21 | 20 | 12 | 8 | 12 | 1 | 2 | 2 |
| S348 | Korean | 37,37 | 9,17 | 12,18 | 19,20 | 16 | 13 | 14 | 30 | 23 | 10 | 11 | 14 | 14 | 10 | 12 | 12 | 26 | 22 | 30 | 15 | 18 | 11 | 24 | 36 | 11 | 12 | 13 | 17 | 15 | 18 | 17 | 15 | 24 | 21 | 9 | 8 | 11 | 2 | 2 | 2 |
| S349 | Korean | 36,40 | 13,13 | 12,17 | 20,24 | 15 | 12 | 12 | 28 | 22 | 11 | 12 | 12 | 15 | 11 | 12 | 13 | 24 | 19 | 34 | 15 | 18 | 11 | 22 | 37 | 12 | 11 | 13 | 15 | 17 | 19 | 16 | 14 | 21 | 21 | 11 | 8 | 11 | 1 | 2 | 2 |
| S350 | Korean | 37,38 | 13,15 | 11,17 | 19,21 | 17 | 13 | 13 | 31 | 24 | 11 | 11 | 14 | 14 | 10 | 11 | 13 | 26 | 21 | 28 | 15 | 16 | 11 | 23 | 38 | 12 | 12 | 12 | 17 | 16 | 17 | 17 | 15 | 23 | 23 | 9 | 8 | 11 | 2 | 2 | 2 |
| S351 | Korean | 36,39 | 13,15 | 12,16 | 21,24 | 15 | 12 | 12 | 29 | 23 | 11 | 12 | 12 | 15 | 10 | 12 | 14 | 25 | 19 | 35 | 15 | 19 | 9 | 23 | 36 | 12 | 11 | 12 | 12 | 16 | 19 | 16 | 14 | 23 | 20 | 11 | 8 | 12 | 1 | 2 | 2 |
| S352 | Korean | 36,40 | 14,14 | 13,18 | 21,21 | 14 | 12 | 13 | 30 | 25 | 11 | 13 | 12 | 14 | 11 | 12 | 15 | 25 | 20 | 32 | 16 | 17 | 10 | 26 | 34 | 11 | 11 | 12 | 14 | 20 | 20 | 16 | 15 | 22 | 21 | 12 | 8 | 10 | 1 | 2 | 2 |
| S353 | Korean | 34,38 | 15,16 | 14,21 | 19,20 | 16 | 15 | 14 | 30 | 23 | 10 | 11 | 15 | 14 | 10 | 10 | 13 | 25 | 21 | 28 | 15 | 17 | 11 | 26 | 40 | 11 | 12 | 13 | 20 | 16 | 17 | 17 | 15 | 21 | 21 | 8 | 8 | 11 | 2 | 2 | 2 |
| S354 | Korean | 35,39 | 14,15 | 12,15 | 20,21 | 15 | 12 | 12 | 30 | 23 | 10 | 10 | 12 | 14 | 10 | 14 | 12 | 24 | 19 | 32 | 15 | 18 | 9 | 22 | 37 | 12 | 11 | 12 | 15 | 16 | 19 | 16 | 14 | 22 | 20 | 12 | 8 | 12 | 1 | 2 | 2 |
| S355 | Korean | 38,39 | 14,14 | 14,19 | 20,23 | 15 | 12 | 12 | 28 | 23 | 10 | 14 | 13 | 15 | 10 | 11 | 14 | 24 | 19 | 30 | 15 | 16 | 10 | 23 | 36 | 12 | 12 | 11 | 14 | 18 | 18 | 16 | 16 | 21 | 21 | 11 | 8 | 11 | 1 | 2 | 2 |
| S356 | Korean | 34,40 | 15,15 | 10,17 | 20,20 | 15 | 12 | 14 | 29 | 23 | 10 | 13 | 13 | 14 | 13 | 12 | 14 | 25 | 18 | 31 | 15 | 16 | 11 | 24 | 38 | 12 | 11 | 12 | 14 | 18 | 19 | 16 | 15 | 20 | 24 | 13 | 8 | 11 | 1 | 2 | 2 |
| S357 | Korean | 36,39 | 13,14 | 13,20 | 22,23 | 15 | 12 | 13 | 30 | 23 | 10 | 13 | 12 | 14 | 10 | 11 | 12 | 22 | 18 | 30 | 16 | 17 | 9 | 23 | 39 | 12 | 12 | 13 | 14 | 16 | 17 | 16 | 15 | 21 | 21 | 11 | 9 | 11 | 1 | 2 | 2 |
| S358 | Korean | 40,40 | 15,15 | 12,19 | 22,22 | 17 | 12 | 12 | 28 | 25 | 10 | 13 | 12 | 16 | 10 | 12 | 13 | 24 | 20 | 32 | 14 | 17 | 9 | 23 | 44 | 12 | 11 | 12 | 14 | 18 | 19 | 17 | 15 | 21 | 21 | 13 | 8 | 12 | 1 | 2 | 2 |
| S359 | Korean | 36,40 | 13,15 | 12,16 | 21,23 | 13 | 12 | 12 | 28 | 23 | 11 | 12 | 12 | 15 | 10 | 12 | 13 | 26 | 19 | 35 | 15 | 18 | 9 | 22 | 36 | 12 | 11 | 12 | 14 | 19 | 19 | 16 | 14 | 22 | 20 | 11 | 8 | 12 | 1 | 2 | 2 |
| S360 | Korean | 37,38 | 16,17 | 11,18 | 19,19 | 15 | 12 | 14 | 31 | 23 | 10 | 11 | 14 | 14 | 10 | 11 | 14 | 23 | 21 | 29 | 15 | 16 | 11 | 29 | 38 | 11 | 12 | 12 | 19 | 19 | 19 | 17 | 15 | 19 | 21 | 8 | 8 | 11 | 2 | 2 | 2 |
| S361 | Korean | 35,40 | 15,16 | 10,19 | 21,22 | 16 | 12 | 14 | 29 | 23 | 10 | 13 | 13 | 14 | 13 | 12 | 14 | 25 | 18 | 31 | 15 | 17 | 11 | 24 | 44 | 11 | 11 | 12 | 14 | 18 | 17 | 16 | 15 | 21 | 21 | 13 | 8 | 11 | 1 | 2 | 2 |
| S362 | Korean | 37,38 | 15,15 | 14,18 | 23,23 | 16 | 12 | 12 | 27 | 25 | 11 | 13 | 12 | 14 | 10 | 12 | 12 | 24 | 19 | 27 | 14 | 17 | 10 | 25 | 40 | 12 | 11 | 13 | 15 | 19.3 | 17 | 16 | 15 | 22 | 22 | 10 | 8 | 12 | 1 | 2 | 2 |
| S363 | Korean | 37,37 | 14,14 | 11,12 | 22,22 | 14 | 13 | 14 | 31 | 23 | 10 | 14 | 13 | 14 | 10 | 11 | 12 | 26 | 20 | 27 | 18 | 16 | 11 | 22 | 37 | 13 | 12 | 12 | 15 | 18 | 16 | 15 | 14 | 21 | 24 | 10 | 8 | 12 | 2 | 2 | 2 |
| S364 | Korean | 37,37 | 13,13 | 12,12 | 24,24 | 14 | 13 | 14 | 31 | 23 | 10 | 14 | 12 | 14 | 11 | 11 | 12 | 24 | 19 | 28 | 16 | 17 | 11 | 22 | 36 | 12 | 12 | 12 | 14 | 20 | 19 | 15 | 14 | 21 | 23 | 10 | 8 | 12 | 2 | 2 | 2 |
| S365 | Korean | 37,40 | 15,15 | 13,13 | 21,23 | 15 | 12 | 12 | 28 | 23 | 11 | 14 | 13 | 14 | 10 | 11 | 11 | 25 | 18 | 31 | 17 | 15 | 10 | 25 | 37 | 10 | 11 | 13 | 16 | 17 | 18 | 15 | 15 | 24 | 19 | 11 | 8 | 13 | 1 | 2 | 2 |
| S366 | Korean | 38,38 | 14,14 | 13,19 | 23,23 | 14 | 10 | 12 | 29 | 24 | 10 | 14 | 12 | 14 | 11 | 12 | 13 | 23 | 20 | 34 | 14 | 17 | 9 | 24 | 35 | 11 | 12 | 12 | 14 | 18 | 18 | 16 | 14 | 20 | 20 | 10 | 8 | 12 | 1 | 1 | 1 |
| S367 | Korean | 37,38 | 14,14 | 13,17 | 21,21 | 15 | 12 | 14 | 30 | 23 | 10 | 13 | 14 | 14 | 10 | 12 | 13 | 27 | 20 | 29 | 17 | 16 | 10 | 24 | 37 | 11 | 12 | 11 | 16 | 17 | 18 | 15 | 16 | 21 | 22 | 11 | 8 | 12 | 1 | 2 | 2 |
| S368 | Korean | 40,41 | 13,14,15 | 12,19 | 22,23,24 | 15 | 12 | 12 | 28 | 24 | 10 | 13 | 12 | 15 | 9 | 11 | 14 | 23 | 19 | 31 | 15 | 15 | 9 | 26 | 38 | 12 | 12 | 15 | 15 | 14 | 17 | 16 | 16 | 19 | 21 | 11 | 9 | 13 | 1 | 2 | 2 |
| S369 | Korean | 37,37 | 15.2,15.2 | 12,13 | 23,23 | 14 | 12 | 13 | 29 | 23 | 10 | 15 | 14 | 14 | 10 | 11 | 12.2 | 30 | 19 | 27 | 15 | 16 | 11 | 22 | 36 | 13.3 | 11 | 12 | 14.1 | 18 | 17 | 15 | 14 | 21 | 21 | 12 | 8 | 12 | 2 | 2 | 2 |
| S370 | Korean | 37,37 | 15.2,16.3 | 11,13 | 19.2,20.2 | 17 | 12 | 13 | 30 | 22 | 10 | 14 | 13 | 14 | 10 | 11 | 12.2 | 25.3 | 19 | 29 | 15 | 20 | 12 | 23 | 36 | 13.3 | 12 | 11 | 14.1 | 17 | 19 | 14.3 | 13.5 | 21 | 22 | 10 | 7.3 | 13 | 2 | 2 | 2 |
| S371 | Korean | 35,37 | 14,15 | 11,17 | 19,22 | 15 | 12 | 14 | 29 | 23 | 10 | 11 | 14 | 14 | 10 | 12 | 13 | 25 | 21 | 29 | 15 | 18 | 11 | 24 | 37 | 11 | 12 | 12 | 17 | 16 | 17 | 17 | 15 | 21 | 23 | 9 | 8 | 11 | 2 | 2 | 2 |
| S372 | Korean | 38,38 | 14,14 | 12,15 | 21,21 | 16 | 13 | 13 | 29 | 23 | 10 | 11 | 14 | 14 | 10 | 11 | 13 | 26 | 21 | 30 | 15 | 16 | 11 | 23 | 37 | 11 | 12 | 13 | 18 | 16 | 21 | 17 | 15 | 21 | 20 | 9 | 8 | 11 | 2 | 2 | 2 |
| S373 | Korean | 37,38 | 15,15 | 14,18 | 23,23 | 17 | 12 | 12 | 27 | 25 | 11 | 13 | 12 | 14 | 10 | 12 | 13 | 24 | 19 | 27 | 14 | 18 | 10 | 25 | 39 | 12 | 10 | 13 | 16 | 19.3 | 17 | 16 | 15 | 22 | 22 | 10 | 8 | 12 | 1 | 2 | 2 |
| S374 | Korean | 37,39 | 14,15 | 11,14 | 21,25 | 17 | 12 | 14 | 31 | 23 | 10 | 11 | 13 | 14 | 11 | 10 | 14 | 24 | 21 | 31 | 15 | 16 | 13 | 22 | 42 | 11 | 12 | 12 | 15 | 20 | 19 | 15 | 16 | 17 | 23 | 10 | 8 | 12 | 2 | 2 | 2 |
| S375 | Korean | 39,39 | 13,13 | 15,19 | 20,20 | 16 | 13 | 12 | 29 | 23 | 10 | 13 | 12 | 15 | 10 | 11 | 14 | 23 | 21 | 32 | 14 | 17 | 11 | 23 | 38 | 12 | 11 | 13 | 14 | 19 | 19 | 17 | 15 | 20 | 20 | 12 | 8 | 12 | 1 | 2 | 2 |
| S376 | Korean | 39,39 | 13,13 | 21,21 | 21,21 | 13 | 12 | 14 | 30 | 24 | 9 | 14 | 14 | 14 | 12 | 11 | 13 | 27 | 19 | 32 | 16 | 17 | 10 | 24 | 37.2 | 10 | 12 | 13 | 19 | 20 | 18 | 15 | 16 | 22 | 22 | 10 | 8 | 10 | 2 | 2 | 2 |
| S377 | Korean | 37,38 | 15,16 | 14,18 | 23,23 | 17 | 12 | 12 | 27 | 25 | 11 | 13 | 12 | 14 | 10 | 11 | 12 | 24 | 19 | 27 | 14 | 19 | 11 | 25 | 39 | 12 | 10 | 12 | 16 | 19.3 | 17 | 16 | 15 | 21 | 22 | 10 | 8 | 12 | 1 | 2 | 2 |
| S378 | Korean | 35,40 | 15,16 | 9,19 | 21,22 | 16 | 12 | 14 | 29 | 23 | 10 | 13 | 13 | 14 | 13 | 12 | 14 | 25 | 18 | 31 | 15 | 17 | 11 | 23 | 41 | 12 | 11 | 12 | 14 | 18 | 17 | 16 | 15 | 21 | 21 | 13 | 8 | 11 | 1 | 2 | 2 |
| S379 | Korean | 37,39 | 14,16 | 13,13 | 22,23 | 15 | 12 | 12 | 28 | 23 | 11 | 15 | 13 | 14 | 10 | 12 | 11 | 26 | 18 | 31.2 | 12 | 17 | 10 | 24 | 38 | 10 | 11 | 12 | 14 | 19 | 20 | 15 | 15 | 21 | 20 | 11 | 8 | 12 | 1 | 2 | 2 |
| S380 | Korean | 35,37 | 13,13 | 13,19 | 21,24 | 14 | 10 | 12 | 27 | 23 | 10 | 14 | 12 | 14 | 11 | 11 | 13 | 23 | 20 | 32 | 15 | 17 | 9 | 20 | 37 | 11 | 11 | 12 | 14 | 19 | 19 | 16 | 14 | 19 | 20 | 11 | 8 | 12 | 1 | 1 | 1 |
| S381 | Korean | 39,41 | 12,14 | 12,15 | 22,22 | 15 | 13 | 12 | 28 | 25 | 10 | 13 | 13 | 15 | 9 | 12 | 12 | 23 | 19 | 30 | 15 | 18 | 10 | 24 | 38 | 12 | 13 | 11 | 15 | 15 | 18 | 16 | 16 | 22 | 22 | 11 | 8 | 12 | 1 | 2 | 2 |
| S382 | Korean | 35,38 | 12,13 | 13,18 | 20,23 | 14 | 10 | 13 | 30 | 23 | 10 | 14 | 12 | 16 | 11 | 13 | 13 | 23 | 20 | 33 | 16 | 17 | 9 | 23 | 35 | 11 | 10 | 12 | 15 | 19 | 20 | 16 | 14 | 19 | 21 | 11 | 8 | 12 | 1 | 1 | 1 |
| S383 | Korean | 35,39 | 14,16 | 9,19 | 21,22 | 16 | 12 | 14 | 30 | 22 | 10 | 13 | 13 | 14 | 13 | 12 | 12 | 25 | 18 | 30 | 15 | 19 | 11 | 19 | 38 | 11 | 11 | 12 | 14 | 19 | 19 | 16 | 15 | 21 | 20 | 13 | 8 | 12 | 1 | 2 | 2 |
| S384 | Korean | 35,40 | 14,17 | 10,18 | 20,22 | 17 | 12 | 14 | 29 | 23 | 10 | 13 | 13 | 14 | 13 | 12 | 14 | 26 | 18 | 32 | 15 | 17 | 10 | 24 | 42 | 11 | 11 | 12 | 13 | 18 | 17 | 16 | 15 | 19 | 22 | 12 | 8 | 11 | 1 | 2 | 2 |
| S385 | Korean | 35,39 | 14,16 | 10,21 | 21,21 | 15 | 12 | 14 | 30 | 22 | 10 | 13 | 13 | 14 | 13 | 10 | 13 | 25 | 18 | 29 | 15 | 18 | 11 | 21 | 39 | 11 | 11 | 12 | 14 | 20 | 20 | 16 | 15 | 20 | 20 | 11 | 8 | 12 | 1 | 2 | 2 |
| S386 | Korean | 40,40 | 13,13 | 12,18 | 20,23 | 15 | 12 | 13 | 29 | 25 | 11 | 13 | 13 | 14 | 10 | 12 | 13 | 24 | 20 | 31 | 16 | 18 | 9 | 24 | 36 | 12 | 12 | 13 | 14 | 14 | 17 | 16 | 17 | 21 | 23 | 11 | 8 | 11 | 1 | 2 | 2 |
| S387 | Korean | 37,38 | 14,15 | 12,18 | 22,23 | 15 | 13 | 12 | 28 | 25 | 10 | 13 | 12 | 14 | 10 | 13 | 12 | 24 | 19 | 31 | 13 | 17 | 10 | 28 | 38 | 12 | 11 | 13 | 16 | 18 | 19 | 17 | 15 | 19 | 21 | 10 | 8 | 12 | 1 | 2 | 2 |
| S388 | Korean | 37,38 | 14,15 | 13,14 | 23,23 | 15 | 12 | 12 | 28 | 23 | 11 | 14 | 13 | 14 | 10 | 12 | 11 | 25 | 18 | 30.2 | 12 | 18 | 11 | 24 | 37 | 10 | 11 | 12 | 14 | 19 | 18 | 15 | 15 | 20 | 20 | 11 | 8 | 11 | 1 | 2 | 2 |
| S389 | Korean | 37,39 | 16,16 | 12,17 | 22,23 | 17 | 14 | 13 | 29 | 24 | 10 | 13 | 12 | 14 | 10 | 12 | 12 | 23 | 19 | 30 | 14 | 22 | 10 | 25 | 38 | 12 | 12 | 12 | 15 | 17 | 19 | 16 | 15 | 23 | 21 | 10 | 8 | 11 | 1 | 2 | 2 |
| S390 | Korean | 35,38 | 13,15 | 11,16 | 20,24 | 15 | 12 | 12 | 28 | 24 | 10 | 12 | 12 | 15 | 10 | 12 | 13 | 25 | 19 | 33 | 15 | 19 | 9 | 22 | 37 | 12 | 9 | 13 | 15 | 17 | 18 | 17 | 14 | 21 | 19 | 11 | 8 | 12 | 1 | 2 | 2 |
| S391 | Korean | 36,39 | 13,14 | 12,16 | 21,24 | 15 | 12 | 12 | 28 | 23 | 11 | 12 | 12 | 15 | 10 | 12 | 13 | 25 | 19 | 34 | 15 | 19 | 9 | 23 | 35 | 12 | 11 | 12 | 12 | 16 | 20 | 16 | 14 | 23 | 20 | 11 | 8 | 12 | 1 | 2 | 2 |
| S392 | Korean | 36,38 | 14,16 | 11,20 | 19,20 | 15 | 12 | 14 | 29 | 23 | 11 | 11 | 14 | 14 | 10 | 12 | 13 | 27 | 21 | 29 | 16 | 18 | 10 | 24 | 35 | 11 | 12 | 12 | 17 | 18 | 18 | 17 | 15 | 23 | 21 | 9 | 8 | 11 | 2 | 2 | 2 |
| S393 | Korean | 37,37 | 15,15 | 12,13 | 22,22 | 14 | 12 | 13 | 29 | 23 | 8 | 15 | 13 | 14 | 10 | 11 | 12 | 29 | 19 | 27 | 15 | 16 | 11 | 22 | 37 | 12 | 12 | 12 | 14 | 17 | 19 | 15 | 14 | 21 | 21 | 12 | 8 | 12 | 2 | 2 | 2 |
| S394 | Korean | 37,38 | 14,16 | 12,21 | 22,23 | 17 | 12 | 12 | 29 | 25 | 10 | 13 | 12 | 14 | 10 | 12 | 12 | 24 | 19 | 29 | 14 | 17 | 10 | 25 | 36 | 12 | 10 | 13 | 16 | 18 | 18 | 16 | 15 | 20 | 21 | 11 | 8 | 11 | 1 | 2 | 2 |
| S395 | Korean | 37,38 | 14,16 | 10,19 | 19,20 | 16 | 12 | 14 | 29 | 23 | 10 | 11 | 14 | 14 | 10 | 11 | 12 | 26 | 21 | 32 | 15 | 18 | 12 | 25 | 37 | 11 | 12 | 13 | 16 | 15 | 18 | 17 | 15 | 23 | 21 | 9 | 7 | 11 | 2 | 2 | 2 |
| S396 | Korean | 38,38 | 13,13 | 15,21 | 20,20 | 16 | 13 | 12 | 29 | 24 | 10 | 13 | 12 | 15 | 10 | 11 | 14 | 23 | 20 | 31 | 15 | 16 | 11 | 25 | 38 | 12 | 12 | 13 | 15 | 18 | 18 | 17 | 15 | 21 | 21 | 11 | 8 | 12 | 1 | 2 | 2 |
| S397 | Korean | 35,41 | 15,15 | 11,18 | 20,21 | 15 | 12 | 13 | 28 | 24 | 10 | 13 | 13 | 14 | 13 | 12 | 14 | 25 | 18 | 32 | 15 | 17 | 10 | 26 | 40 | 11 | 11 | 12 | 14 | 18 | 17 | 16 | 14 | 21 | 21 | 13 | 8 | 11 | 1 | 2 | 2 |
| S398 | Korean | 36,38 | 14,15 | 15,17 | 21,23 | 15 | 12 | 13 | 29 | 23 | 11 | 12 | 12 | 14 | 10 | 11 | 13 | 24 | 19 | 32 | 16 | 18 | 11 | 22 | 41 | 11 | 11 | 13 | 15 | 17 | 16 | 15 | 15 | 20 | 21 | 11 | 8 | 12 | 1 | 2 | 2 |
| S399 | Korean | 37,37 | 14,15 | 12,18 | 22,23 | 15 | 13 | 12 | 28 | 25 | 10 | 13 | 12 | 14 | 10 | 14 | 12 | 24 | 19 | 31 | 13 | 17 | 10 | 28 | 38 | 13 | 11 | 13 | 16 | 18 | 19 | 17 | 15 | 19 | 21 | 10 | 8 | 12 | 1 | 2 | 2 |
| S400 | Korean | 38,38 | 14,14 | 10,20 | 19,20 | 15 | 12 | 14 | 30 | 24 | 10 | 11 | 15 | 14 | 10 | 12 | 13 | 27 | 21 | 31 | 15 | 15 | 10 | 25 | 40 | 11 | 11 | 12 | 17 | 15 | 18 | 17 | 15 | 23 | 22 | 9 | 8 | 10 | 2 | 2 | 2 |
| S401 | Korean | 40,40 | 14,14 | 11,12 | 22,22 | 14 | 12 | 14 | 30 | 22 | 10 | 14 | 13 | 14 | 10 | 10 | 13 | 25 | 19 | 30 | 16 | 17 | 11 | 21 | 35 | 11 | 12 | 12 | 15 | 20 | 16 | 15 | 14 | 21 | 21 | 11 | 8 | 11 | 2 | 2 | 2 |
| S402 | Korean | 36,39 | 15,16 | 10,17 | 20,21 | 16 | 12 | 14 | 30 | 23 | 10 | 13 | 13 | 14 | 13 | 12 | 14 | 25 | 19 | 30 | 15 | 18 | 11 | 23 | 42 | 10 | 11 | 12 | 14 | 17 | 17 | 16 | 15 | 20 | 21 | 12 | 8 | 11 | 1 | 2 | 2 |
| S403 | Korean | 34.1,38 | 14,17 | 10,18 | 19,21 | 15 | 12 | 14 | 30 | 22 | 10 | 13 | 13 | 14 | 13 | 12 | 12 | 24 | 18 | 30 | 15 | 18 | 12 | 21 | 39 | 11 | 11 | 12 | 14 | 21 | 18 | 16 | 15 | 21 | 20 | 12 | 8 | 12 | 1 | 2 | 2 |
| S404 | Korean | 34,40 | 14,14 | 15,19 | 21,21 | 16 | 12 | 13 | 28 | 25 | 10 | 12 | 12 | 15 | 10 | 13 | 13 | 22 | 21 | 33 | 14 | 18 | 9 | 23 | 42 | 12 | 11 | 12 | 14 | 20 | 18 | 16 | 13 | 23 | 21 | 11 | 9 | 12 | 1 | 2 | 2 |
| S405 | Korean | 34,39 | 13,14 | 13,19 | 20,25 | 14 | 10 | 12 | 30 | 24 | 10 | 14 | 12 | 15 | 11 | 12 | 11 | 23 | 20 | 33 | 15 | 19 | 9 | 23 | 36 | 11 | 11 | 12 | 14 | 18 | 19 | 16 | 14 | 22 | 22 | 11 | 8 | 13 | 1 | 1 | 1 |
| S406 | Korean | 35,37 | 13,14 | 13,18 | 21,23 | 15 | 10 | 12 | 28 | 24 | 10 | 14 | 12 | 16 | 11 | 12 | 13 | 23 | 21 | 34 | 15 | 17 | 9 | 23 | 34 | 11 | 11 | 12 | 13 | 17 | 18 | 16 | 14 | 18 | 20 | 11 | 8 | 12 | 1 | 1 | 1 |
| S407 | Korean | 35,39 | 16,16 | 10,21 | 20,20 | 16 | 12 | 14 | 29 | 23 | 10 | 13 | 13 | 14 | 13 | 14 | 14 | 25 | 18 | 30 | 15 | 18 | 11 | 23 | 38 | 11 | 11 | 13 | 14 | 17 | 18 | 16 | 15 | 21 | 21 | 13 | 8 | 11 | 1 | 2 | 2 |
| S408 | Korean | 38,39 | 12,17 | 13,18 | 20,22 | 16 | 12 | 13 | 30 | 25 | 11 | 13 | 14 | 14 | 10 | 13 | 11 | 27 | 18 | 26 | 15 | 19 | 10 | 23 | 43 | 13 | 10 | 11 | 15 | 16 | 19 | 16 | 15 | 19 | 22 | 12 | 8 | 11 | 1 | 2 | 2 |
| S409 | Korean | 34,34 | 14,14 | 11,12 | 22,22 | 15 | 12 | 15 | 30 | 21 | 10 | 14 | 13 | 14 | 10 | 10 | 13 | 25 | 18 | 28 | 14 | 18 | 10 | 20 | 35 | 12 | 14 | 12 | 14 | 18 | 18 | 15 | 14 | 22 | 20 | 12 | 8 | 11 | 2 | 2 | 2 |
| S410 | Korean | 35,39 | 14,17 | 10,20 | 21,21 | 15 | 12 | 13 | 29 | 22 | 10 | 14 | 13 | 14 | 13 | 12 | 12 | 25 | 18 | 30 | 15 | 19 | 11 | 21 | 38 | 11 | 11 | 12 | 13 | 18 | 18 | 16 | 15 | 22 | 20 | 12 | 8 | 12 | 1 | 2 | 2 |
| S411 | Korean | 35,37 | 17,17 | 12,18 | 19,20 | 16 | 13 | 14 | 30 | 23 | 10 | 11 | 14 | 14 | 10 | 12 | 12 | 28 | 22 | 30 | 15 | 18 | 11 | 24 | 36 | 11 | 12 | 14 | 17 | 16 | 19 | 17 | 15 | 23 | 21 | 9 | 8 | 11 | 2 | 2 | 2 |
| S412 | Korean | 37,38 | 14,15 | 13,14 | 22,23 | 15 | 12 | 12 | 28 | 23 | 11 | 14 | 13 | 14 | 10 | 12 | 11 | 25 | 18 | 30.2 | 12 | 18 | 11 | 24 | 37 | 10 | 11 | 12 | 14 | 19 | 18 | 15 | 15 | 20 | 20 | 11 | 8 | 12 | 1 | 2 | 2 |
| S413 | Korean | 34,38 | 15,17 | 11,18 | 20,21 | 15 | 12 | 14 | 30 | 23 | 10 | 13 | 13 | 14 | 13 | 13 | 12 | 25 | 18 | 31 | 14 | 19 | 11 | 21 | 39 | 11 | 11 | 12 | 14 | 19 | 20 | 16 | 15 | 22 | 20 | 12 | 8 | 12 | 1 | 2 | 2 |
| S414 | Korean | 35,40 | 14,14 | 11,12 | 21,22 | 16 | 12 | 13 | 28 | 22 | 10 | 14 | 12 | 15 | 10 | 11 | 13 | 25 | 19 | 35 | 14 | 16 | 9 | 22 | 37 | 12 | 11 | 13 | 15 | 17 | 18 | 16 | 15 | 22 | 20 | 10 | 8 | 12 | 1 | 2 | 2 |
| S415 | Korean | 36,37 | 16,16 | 11,18 | 19,20 | 15 | 13 | 14 | 30 | 23 | 10 | 11 | 14 | 14 | 10 | 11 | 15 | 25 | 21 | 30 | 15 | 19 | 11 | 27 | 42 | 11 | 12 | 12 | 18 | 19 | 18 | 17 | 15 | 19 | 21 | 8 | 8 | 11 | 2 | 2 | 2 |
| S416 | Korean | 37,38 | 14,14 | 11,17 | 20,22 | 15 | 13 | 13 | 32 | 25 | 10 | 11 | 14 | 14 | 10 | 12 | 13 | 25 | 21 | 32 | 15 | 15 | 11 | 24 | 38 | 12 | 12 | 12 | 17 | 17 | 17 | 17 | 15 | 24 | 21 | 9 | 8 | 11 | 2 | 2 | 2 |
| S417 | Korean | 35,40 | 15,15 | 10,19 | 20,21 | 16 | 12 | 13 | 28 | 24 | 10 | 13 | 13 | 14 | 13 | 11 | 13 | 25 | 18 | 30 | 15 | 17 | 10 | 23 | 41 | 11 | 11 | 12 | 15 | 17 | 17 | 16 | 15 | 20 | 21 | 12 | 9 | 11 | 1 | 2 | 2 |
| S418 | Korean | 35,41 | 15,16 | 10,19 | 21,22 | 15 | 12 | 13 | 28 | 23 | 10 | 13 | 13 | 14 | 13 | 11 | 14 | 24 | 18 | 31 | 15 | 17 | 11 | 23 | 41 | 10 | 11 | 12 | 14 | 18 | 17 | 16 | 15 | 20 | 21 | 14 | 8 | 11 | 1 | 2 | 2 |
| S419 | Korean | 38,39 | 15,15 | 12,19 | 22,23 | 16 | 12 | 12 | 28 | 24 | 10 | 13 | 12 | 15 | 10 | 11 | 12 | 24 | 19 | 32 | 15 | 17 | 10 | 24 | 37 | 13 | 11 | 13 | 16 | 19 | 19 | 16 | 15 | 19 | 22 | 10 | 8 | 12 | 1 | 2 | 2 |
| S420 | Korean | 34,39 | 14,16 | 10,20 | 21,23 | 15 | 12 | 15 | 31 | 24 | 11 | 13 | 14 | 14 | 13 | 12 | 14 | 25 | 17 | 28 | 15 | 17 | 11 | 23 | 40 | 12 | 11 | 12 | 15 | 19 | 18 | 16 | 15 | 23 | 20 | 12 | 8 | 12 | 1 | 2 | 2 |
| S421 | Korean | 36,40 | 15,15 | 12,17 | 20,24 | 15 | 12 | 12 | 30 | 23 | 10 | 12 | 11 | 16 | 10 | 12 | 13 | 24 | 19 | 33 | 15 | 18 | 9 | 23 | 37 | 14 | 11 | 14 | 15 | 17 | 19 | 16 | 14 | 20 | 19 | 11 | 8 | 13 | 1 | 2 | 2 |
| S422 | Korean | 35,40 | 15,17 | 10,10 | 20,22 | 16 | 12 | 14 | 29 | 23 | 10 | 13 | 13 | 14 | 13 | 12 | 13 | 25 | 19 | 30 | 15 | 18 | 11 | 23 | 39 | 11 | 11 | 12 | 14 | 18 | 17 | 16 | 15 | 20 | 21 | 12 | 8 | 11 | 1 | 2 | 2 |
| S423 | Korean | 35,40 | 15,15 | 10,19 | 21,22 | 17 | 12 | 14 | 29 | 23 | 10 | 13 | 13 | 14 | 13 | 11 | 14 | 25 | 18 | 31 | 15 | 17 | 11 | 23 | 41 | 11 | 11 | 12 | 14 | 20 | 18 | 16 | 15 | 21 | 21 | 13 | 8 | 11 | 1 | 2 | 2 |
| S424 | Korean | 37,39 | 14,14 | 13,19 | 21,22 | 14 | 10 | 12 | 28 | 24 | 10 | 14 | 12 | 15 | 11 | 11 | 12 | 23 | 20 | 31 | 15 | 17 | 10 | 23 | 37 | 11 | 10 | 12 | 14 | 19 | 19 | 16 | 15 | 19 | 20 | 12 | 8 | 12 | 1 | 1 | 1 |
| S425 | Korean | 35,42 | 15,16 | 10,18 | 22,23 | 15 | 12 | 13 | 27 | 23 | 10 | 13 | 14 | 14 | 13 | 13 | 12 | 25 | 18 | 28 | 16 | 17 | 10 | 21 | 37 | 12 | 11 | 13 | 14 | 18 | 17 | 16 | 15 | 20 | 20 | 13 | 8 | 12 | 1 | 2 | 2 |
| S426 | Korean | 37,40 | 14,15 | 11,17 | 18,21 | 15 | 12 | 14 | 29 | 23 | 10 | 11 | 15 | 14 | 10 | 14 | 12 | 26 | 21 | 29 | 15 | 17 | 10 | 24 | 37 | 11 | 12 | 12 | 18 | 17 | 18 | 17 | 15 | 22 | 21 | 9 | 8 | 11 | 2 | 2 | 2 |
| S427 | Korean | 35,36 | 9,17 | 13,18 | 19,20 | 16 | 13 | 13 | 29 | 23 | 10 | 11 | 14 | 14 | 10 | 13 | 12 | 28 | 23 | 30 | 15 | 18 | 11 | 24 | 36 | 11 | 12 | 14 | 17 | 17 | 18 | 17 | 14 | 24 | 21 | 9 | 8 | 11 | 2 | 2 | 2 |
| S428 | Korean | 35,39 | 16,17 | 10,17 | 22,24 | 15 | 12 | 12 | 27 | 23 | 10 | 13 | 13 | 14 | 13 | 12 | 13 | 24 | 18 | 28 | 15 | 17 | 10 | 21 | 36 | 9 | 11 | 13 | 14 | 19 | 18 | 16 | 15 | 20 | 20 | 12 | 8 | 12 | 1 | 2 | 2 |
| S429 | Korean | 36,39 | 14,14 | 11,11 | 21,22 | 16 | 12 | 13 | 28 | 23 | 10 | 14 | 12 | 14 | 10 | 11 | 12 | 26 | 19 | 31 | 16 | 17 | 9 | 23 | 38 | 12 | 11 | 14 | 15 | 19 | 19 | 16 | 15 | 22 | 20 | 10 | 8 | 13 | 1 | 2 | 2 |
| S430 | Korean | 34,40 | 13,13 | 12,19 | 21,22 | 15 | 10 | 12 | 30 | 25 | 10 | 13 | 12 | 14 | 11 | 11 | 13 | 23 | 20 | 31 | 15 | 16 | 9 | 26 | 36 | 12 | 12 | 12 | 13 | 19 | 16 | 16 | 14 | 20 | 21 | 11 | 9 | 12 | 1 | 1 | 2 |
| S431 | Korean | 36,38 | 14,15 | 14,16 | 23,25 | 15 | 10 | 13 | 29 | 24 | 10 | 14 | 12 | 15 | 11 | 12 | 12 | 23 | 20 | 32 | 14 | 16 | 9 | 24 | 36 | 13 | 11 | 12 | 14 | 16 | 19 | 16 | 14 | 20 | 20 | 11 | 8 | 12 | 1 | 1 | 1 |
| S432 | Korean | 37,38 | 15,15 | 14,18 | 22,23 | 17 | 12 | 12 | 27 | 25 | 10 | 13 | 12 | 14 | 10 | 12 | 12 | 24 | 19 | 27 | 14 | 19 | 10 | 24 | 37 | 13 | 10 | 13 | 16 | 19.3 | 17 | 16 | 15 | 22 | 23 | 10 | 8 | 13 | 1 | 2 | 2 |
| S433 | Korean | 39,39 | 14,15 | 13,13 | 19,20 | 16,17 | 13 | 13 | 30 | 23 | 9 | 11 | 13 | 14 | 10 | 11 | 12 | 26 | 20 | 30 | 15 | 18 | 12 | 26 | 39 | 11 | 11 | 12 | 15 | 18 | 19 | 16 | 16 | 24 | 23 | 10 | 9 | 10 | 2 | 2 | 2 |
| S434 | Korean | 35,39,40 | 15,16 | 13,15 | 20,22 | 13 | 13 | 13 | 29 | 24 | 11 | 11 | 14 | 14 | 10 | 12 | 12 | 24 | 18 | 33 | 16 | 16 | 10 | 26 | 39 | 11 | 11 | 12 | 16 | 20 | 18 | 16 | 17 | 19 | 21 | 11 | 8 | 13 | 2 | 2 | 2 |
| S435 | Korean | 36,39 | 13,15 | 12,17 | 21,24 | 15 | 12 | 12 | 28 | 23 | 11 | 12 | 12 | 14 | 10 | 12 | 13 | 24 | 19 | 32 | 16 | 20 | 10 | 22 | 37 | 12 | 12 | 12 | 15 | 16 | 20 | 16 | 14 | 22 | 19 | 11 | 8 | 11 | 1 | 2 | 2 |
| S436 | Korean | 39,39 | 14,14 | 13,13 | 21,23 | 16 | 12 | 12 | 29 | 23 | 10 | 14 | 13 | 14 | 10 | 11 | 11 | 25 | 19 | 30 | 17 | 14 | 11 | 26 | 38 | 10 | 11 | 12 | 15 | 18 | 18 | 15 | 15 | 24 | 19 | 11 | 8 | 12 | 1 | 2 | 2 |
| S437 | Korean | 36,37 | 14,15 | 12,14 | 20,22 | 16 | 12 | 12 | 28 | 23 | 10 | 14 | 13 | 14 | 10 | 12 | 10 | 25 | 19 | 32 | 16 | 18 | 10 | 24 | 37 | 9 | 11 | 13 | 14 | 18 | 18 | 15 | 15 | 19.2 | 20 | 11 | 8 | 12 | 1 | 2 | 2 |
| S438 | Korean | 37,40 | 13,16 | 12,13 | 21,23 | 14 | 12 | 14 | 29 | 23 | 10 | 15 | 13 | 14 | 11 | 10 | 12 | 23 | 21 | 29 | 16 | 16 | 11 | 22 | 33 | 11 | 12 | 12 | 16 | 17 | 19 | 15 | 14 | 17 | 21 | 11 | 9 | 13 | 2 | 2 | 2 |
| S439 | Korean | 38,41 | 13,14 | 12,17 | 21,23 | 15 | 12 | 14 | 30 | 23 | 11 | 13 | 14 | 14 | 10 | 12 | 14 | 27 | 18 | 29 | 15 | 17 | 12 | 23 | 39 | 11 | 10 | 12 | 17 | 20 | 17 | 15 | 15 | 19 | 22 | 11 | 8 | 13 | 1 | 2 | 2 |
| S440 | Korean | 36,38 | 13,15 | 14,17 | 22,22 | 15 | 12 | 12 | 28 | 24 | 10 | 13 | 12 | 14 | 10 | 11 | 14 | 25 | 20 | 28 | 15 | 16 | 10 | 23 | 41 | 13 | 11 | 13 | 16 | 21 | 18 | 16 | 15 | 21 | 22 | 10 | 8 | 12 | 1 | 2 | 2 |
| S441 | Korean | 35,41 | 15,15 | 10,18 | 22,23 | 15 | 12 | 13 | 27 | 23 | 10 | 13 | 13 | 14 | 13 | 13 | 12 | 26 | 18 | 28 | 15 | 18 | 10 | 21 | 37 | 12 | 11 | 12 | 14 | 19 | 18 | 16 | 15 | 23 | 20 | 12 | 8 | 12 | 1 | 2 | 2 |
| S442 | Korean | 37,37 | 15,15 | 12,13 | 22,22 | 14 | 12 | 13 | 29 | 23 | 10 | 14 | 13 | 14 | 10 | 12 | 12 | 27 | 19 | 26 | 15 | 17 | 11 | 22 | 34 | 12 | 12 | 13 | 14 | 17 | 19 | 15 | 14 | 20 | 21 | 12 | 8 | 11 | 2 | 2 | 2 |
| S443 | Korean | 37,37 | 13,13 | 13,23 | 20,20 | 15 | 12 | 12 | 29 | 25 | 10 | 13 | 12 | 15 | 10 | 11 | 14 | 23 | 20 | 30 | 13 | 17 | 10 | 24 | 38.2 | 12 | 11 | 13 | 17 | 20 | 19 | 17 | 15 | 21 | 21 | 12 | 8 | 13 | 1 | 2 | 2 |
| S444 | Korean | 37,37 | 16,16 | 14,19 | 23,23 | 16 | 13 | 12 | 27 | 25 | 10 | 13 | 12 | 14 | 10 | 12 | 12 | 24 | 19 | 27 | 14 | 17 | 10 | 26 | 41 | 12 | 10 | 13 | 16 | 19.3 | 17 | 16 | 15 | 21 | 23 | 10 | 8 | 12 | 1 | 2 | 2 |
| S445 | Korean | 35,39 | 14,19 | 10,18 | 20,21 | 16 | 12 | 13 | 28 | 24 | 10 | 13 | 13 | 14 | 13 | 12 | 11 | 25 | 18 | 30 | 15 | 16 | 12 | 24 | 40 | 11 | 11 | 12 | 14 | 17 | 19 | 16 | 15 | 20 | 22 | 12 | 8 | 11 | 1 | 2 | 2 |
| S446 | Korean | 36,39 | 14,15 | 12,12 | 22,22 | 17 | 12 | 14 | 29 | 23 | 11 | 12 | 12 | 15 | 10 | 13 | 12 | 25 | 18 | 32 | 15 | 19 | 9 | 22 | 37 | 12 | 11 | 12 | 15 | 16 | 19 | 16 | 14 | 22 | 19 | 11 | 8 | 12 | 1 | 2 | 2 |
| S447 | Korean | 34,35,36 | 13,14 | 11,18 | 19,20,21 | 15 | 13 | 13 | 29 | 23 | 10 | 11 | 15 | 14 | 10 | 11 | 12 | 26 | 21 | 30 | 15 | 17 | 11 | 24 | 37 | 11 | 13 | 13 | 20.1 | 16 | 15 | 17 | 15 | 21 | 21 | 10 | 8 | 12 | 2 | 2 | 2 |
| S448 | Korean | 37,37 | 15,15 | 11,13 | 21,22 | 16 | 12 | 13 | 29 | 22 | 10 | 15 | 13 | 14 | 10 | 12 | 12 | 26 | 19 | 29 | 15 | 20 | 11 | 23 | 37 | 13 | 11 | 11 | 14 | 17 | 19 | 15 | 14 | 21 | 21 | 11 | 8 | 12 | 2 | 2 | 2 |
| S449 | Korean | 37,37 | 15,15 | 11,18 | 19,21 | 16 | 13 | 13 | 29 | 25 | 10 | 11 | 15 | 14 | 10 | 12 | 14 | 25 | 21 | 31 | 15 | 15 | 11 | 26 | 41 | 12 | 12 | 12 | 19 | 16 | 16 | 18 | 15 | 21 | 21 | 8 | 8 | 11 | 2 | 2 | 2 |
| S450 | Korean | 36,37 | 13,14 | 13,19 | 20,24 | 14 | 10 | 12 | 28 | 24 | 10 | 14 | 12 | 15 | 11 | 13 | 12 | 23 | 20 | 32 | 15 | 18 | 9 | 24 | 35 | 10 | 11 | 12 | 14 | 18 | 20 | 16 | 14 | 24 | 20 | 11 | 8 | 12 | 1 | 1 | 1 |
| S451 | Korean | 35,39 | 14,16 | 10,18 | 21,21 | 15 | 12 | 14 | 30 | 22 | 10 | 13 | 13 | 14 | 13 | 12 | 13 | 25 | 18 | 30 | 16 | 19 | 11 | 21 | 37 | 11 | 11 | 12 | 14 | 20 | 17 | 16 | 15 | 24 | 20 | 13 | 8 | 12 | 1 | 2 | 2 |
| S452 | Korean | 38,38 | 16,16 | 11,17 | 21,21 | 16 | 13 | 14 | 30 | 23 | 10 | 11 | 15 | 15 | 10 | 12 | 13 | 26 | 21 | 30 | 15 | 16 | 10 | 24 | 37 | 11 | 13 | 13 | 16 | 18 | 18 | 17 | 15 | 17 | 22 | 9 | 8 | 11 | 2 | 2 | 2 |
| S453 | Korean | 37,39 | 13,13 | 16,20 | 20,20 | 16 | 13 | 12 | 30 | 24 | 10 | 13 | 12 | 15 | 10 | 11 | 14 | 24 | 20 | 32 | 15 | 16 | 12 | 23 | 40 | 12 | 11 | 13 | 14 | 18 | 18 | 17 | 15 | 20 | 21 | 11 | 8 | 12 | 1 | 2 | 2 |
| S454 | Korean | 37,39 | 13,14 | 13,13 | 22,23 | 15 | 12 | 13 | 28 | 23 | 11 | 14 | 13 | 14 | 10 | 12 | 11 | 26 | 18 | 30.2 | 12 | 15 | 10 | 24 | 38 | 10 | 11 | 12 | 13 | 17 | 20 | 15 | 16 | 20 | 20 | 11 | 8 | 12 | 1 | 2 | 2 |
| S455 | Korean | 36,36 | 14,14 | 11,14 | 23,23 | 14 | 12 | 13 | 29 | 21 | 10 | 15 | 13 | 14 | 9 | 11 | 12 | 26 | 19 | 30 | 16 | 16 | 12 | 24 | 38 | 12 | 12 | 13 | 14 | 20 | 20 | 15 | 14 | 23 | 21 | 10 | 8 | 12 | 2 | 2 | 2 |
| S456 | Korean | 37,37 | 14,16 | 11,20 | 19,20 | 16 | 12 | 14 | 29 | 23 | 11 | 12 | 14 | 14 | 10 | 14 | 13 | 26 | 21 | 28 | 15 | 17 | 11 | 25 | 36 | 11 | 12 | 13 | 17 | 16 | 16 | 17 | 15 | 21 | 22 | 9 | 8 | 10 | 2 | 2 | 2 |
| S457 | Korean | 37,39 | 14,14 | 13,13 | 22,23 | 15 | 12 | 12 | 28 | 23 | 11 | 14 | 13 | 14 | 10 | 12 | 11 | 25 | 18 | 31.2 | 12 | 17 | 11 | 24 | 38 | 10 | 11 | 12 | 14 | 18 | 19 | 15 | 15 | 22 | 20 | 11 | 8 | 13 | 1 | 2 | 2 |
| S458 | Korean | 35,41 | 15,15 | 10,19 | 21,24 | 15 | 12 | 14 | 30 | 23 | 10 | 13 | 13 | 14 | 13 | 12 | 14 | 25 | 17 | 31 | 15 | 16 | 11 | 23 | 42 | 11 | 11 | 12 | 14 | 18 | 17 | 16 | 15 | 22 | 21 | 13 | 8 | 11 | 1 | 2 | 2 |
| S459 | Korean | 37,38 | 14,16 | 14,17 | 22,23 | 17 | 12 | 12 | 27 | 25 | 10 | 13 | 12 | 14 | 10 | 12 | 12 | 24 | 19 | 29 | 14 | 17 | 10 | 25 | 39 | 12 | 10 | 12 | 17 | 19.3 | 17 | 16 | 15 | 22 | 22 | 10 | 8 | 12 | 1 | 2 | 2 |
| S460 | Korean | 35,40 | 14,16 | 10,18 | 22,23 | 15 | 12 | 14 | 30 | 22 | 10 | 13 | 13 | 14 | 13 | 13 | 12 | 25 | 18 | 29 | 15 | 18 | 11 | 22 | 38 | 11 | 11 | 12 | 14 | 19 | 18 | 16 | 15 | 24 | 20 | 10 | 8 | 12 | 1 | 2 | 2 |
| S461 | Korean | 35,35 | 12,12 | 15,17 | 23,23 | 14 | 12 | 13 | 29 | 24 | 10 | 11 | 13 | 14 | 10 | 10 | 13 | 27 | 18 | 29 | 14 | 18 | 11 | 27 | 39 | 11 | 11 | 13 | 19 | 18 | 17 | 15 | 16 | 22 | 21 | 10 | 8 | 11 | 2 | 2 | 2 |
| S462 | Korean | 37,39 | 14,14 | 11,18 | 21,21 | 14 | 13 | 14 | 30 | 23 | 10 | 14 | 14 | 14 | 10 | 11 | 13 | 25 | 18 | 29 | 15 | 18 | 11 | 28 | 41 | 12 | 13 | 13 | 15 | 20 | 17 | 15 | 15 | 20 | 20 | 11 | 8 | 12 | 1 | 2 | 2 |
| S463 | Korean | 38,39 | 13,14 | 12,17 | 21,23 | 15 | 12 | 14 | 30 | 24 | 10 | 13 | 14 | 14 | 10 | 11 | 13 | 28 | 18 | 29 | 15 | 15 | 11 | 25 | 36 | 11 | 11 | 12 | 16 | 23 | 18 | 15 | 15 | 20 | 21 | 11 | 8 | 11 | 1 | 2 | 2 |
| S464 | Korean | 37,39 | 13,13 | 15,20 | 20,20 | 16 | 13 | 12 | 30 | 24 | 10 | 13 | 12 | 15 | 10 | 11 | 14 | 24 | 20 | 32 | 15 | 16 | 12 | 23 | 40 | 12 | 11 | 13 | 14 | 18 | 18 | 17 | 15 | 20 | 21 | 11 | 8 | 12 | 1 | 2 | 2 |
| S465 | Korean | 37,37 | 15,15 | 11,12,13 | 30,30 | 14 | 13 | 14 | 31 | 23 | 10 | 14 | 13 | 14 | 10 | 11 | 13 | 27 | 20 | 28 | 17 | 17 | 11 | 22 | 37 | 12 | 12 | 13 | 14 | 19 | 15 | 16 | 14 | 21 | 23 | 10 | 8 | 12 | 2 | 2 | 2 |
| S466 | Korean | 36,37 | 14,15 | 14,17 | 22,22 | 15 | 12 | 13 | 28 | 24 | 10 | 13 | 12 | 14 | 10 | 11 | 12 | 24 | 20 | 29 | 14 | 18 | 10 | 22 | 41 | 11 | 11 | 12 | 17 | 20 | 18 | 16 | 15 | 19 | 26 | 10 | 8 | 12 | 1 | 2 | 2 |
| S467 | Korean | 35,39 | 15,16 | 19,19 | 21,21 | 15 | 12 | 14 | 30 | 22 | 10 | 13 | 13 | 14 | 13 | 11 | 12 | 25 | 18 | 30 | 15 | 17 | 11 | 22 | 39 | 11 | 11 | 12 | 14 | 20 | 20 | 16 | 15 | 22 | 20 | 12 | 8 | 12 | 1 | 2 | 2 |
| S468 | Korean | 35,39,41 | 15,16 | 13,15 | 19,22,23 | 13 | 13 | 13 | 29 | 24 | 10 | 11 | 14 | 14 | 10 | 12 | 13 | 23 | 18 | 33 | 15 | 18 | 11 | 26 | 36 | 11 | 12 | 12 | 17 | 18 | 19 | 16 | 17 | 19 | 24 | 11 | 8 | 12 | 2 | 2 | 2 |
| S469 | Korean | 36,38 | 14,14 | 13,19 | 22,24 | 15 | 12 | 12 | 29 | 27 | 10 | 13 | 12 | 14 | 10 | 11 | 12 | 25 | 19 | 33 | 14 | 20 | 10 | 23 | 40 | 13 | 11 | 11 | 15 | 18 | 17 | 16 | 15 | 20 | 23 | 10 | 8 | 11 | 1 | 2 | 2 |
| S470 | Korean | 37,38 | 12,13 | 16,20 | 20,21 | 17 | 13 | 12 | 30 | 24 | 10 | 13 | 12 | 15 | 10 | 12 | 14 | 24 | 20 | 30 | 16 | 16 | 11 | 23 | 37 | 13 | 12 | 13 | 14 | 18 | 17 | 17 | 15 | 20 | 21 | 11 | 8 | 12 | 1 | 2 | 2 |
| S471 | Korean | 36,37 | 14,16 | 11,20 | 19,20 | 15 | 13 | 13 | 29 | 23 | 10 | 11 | 15 | 14 | 10 | 11 | 12 | 25 | 21 | 28 | 15 | 17 | 11 | 25 | 38 | 11 | 12 | 12 | 17 | 16 | 17 | 18 | 15 | 21 | 22 | 9 | 8 | 12 | 2 | 2 | 2 |
| S472 | Korean | 35,39 | 14,15 | 10,19 | 21,21 | 15 | 12 | 14 | 29 | 22 | 10 | 13 | 13 | 14 | 13 | 12 | 12 | 25 | 18 | 31 | 16 | 20 | 11 | 21 | 37 | 11 | 12 | 12 | 13 | 20 | 18 | 16 | 15 | 20 | 20 | 12 | 8 | 12 | 1 | 2 | 2 |
| S473 | Korean | 36,39 | 15,17 | 10,20 | 22,22 | 15 | 12 | 14 | 30 | 23 | 10 | 13 | 13 | 14 | 13 | 11 | 12 | 25 | 18 | 30 | 16 | 17 | 10 | 21 | 39 | 11 | 12 | 13 | 14 | 21 | 19 | 16 | 15 | 22 | 19 | 12 | 8 | 12 | 1 | 2 | 2 |
| S474 | Korean | 39,39 | 15,15 | 15,21 | 21,21 | 13 | 12 | 14 | 30 | 23 | 9 | 14 | 14 | 14 | 12 | 11 | 13 | 27 | 19 | 30 | 15 | 16 | 10 | 24 | 35.2 | 10 | 12 | 12 | 19 | 19 | 18 | 15 | 16 | 20 | 22 | 10 | 8 | 10 | 2 | 2 | 2 |
| S475 | Korean | 36,37 | 13,16 | 13,17 | 23,24 | 15 | 12 | 13 | 30 | 25 | 10 | 13 | 14 | 14 | 10 | 12 | 13 | 25 | 19 | 24 | 14 | 18 | 10 | 23 | 40 | 13 | 10 | 12 | 16 | 17 | 17 | 15 | 15 | 22 | 23 | 10 | 8 | 10 | 1 | 2 | 2 |
| S476 | Korean | 34,41 | 13,13 | 12,19 | 21,24 | 16 | 10 | 12 | 28 | 25 | 10 | 14 | 12 | 15 | 12 | 11 | 12 | 23 | 20 | 29 | 15 | 13 | 10 | 25 | 37 | 12 | 12 | 12 | 16 | 17 | 18 | 16 | 14 | 23 | 21 | 12 | 8 | 12 | 1 | 1 | 2 |
| S477 | Korean | 35,37 | 17,17 | 12,18 | 19,20 | 16 | 13 | 13 | 29 | 23 | 10 | 11 | 14 | 14 | 10 | 12 | 12 | 28 | 22 | 31 | 15 | 17 | 11 | 24 | 36 | 11 | 12 | 15 | 16 | 15 | 17 | 17 | 15 | 21 | 21 | 9 | 8 | 11 | 2 | 2 | 2 |
| S478 | Korean | 35,37 | 9,17 | 12,18 | 19,20 | 16 | 13 | 13 | 29 | 23 | 10 | 11 | 14 | 14 | 10 | 13 | 12 | 28 | 22 | 30 | 15 | 18 | 11 | 24 | 35 | 11 | 12 | 14 | 17 | 16 | 18 | 17 | 14 | 24 | 21 | 9 | 8 | 11 | 2 | 2 | 2 |
| S479 | Korean | 37,39 | 12,13 | 15,20 | 20,20 | 17 | 13 | 12 | 30 | 24 | 10 | 13 | 12 | 15 | 10 | 11 | 12 | 24 | 20 | 31 | 15 | 17 | 11 | 23 | 39 | 12 | 11 | 13 | 14 | 18 | 18 | 17 | 15 | 22 | 22 | 11 | 8 | 13 | 1 | 2 | 2 |
| S480 | Korean | 36,38 | 12,16 | 12,18 | 22,23 | 16 | 13 | 12 | 28 | 24 | 10 | 13 | 12 | 14 | 10 | 12 | 13 | 24 | 19 | 30 | 15 | 17 | 11 | 27 | 38 | 12 | 11 | 13 | 16 | 18 | 19 | 17 | 15 | 21 | 22 | 11 | 8 | 11 | 1 | 2 | 2 |
| S481 | Korean | 35,40 | 16,16 | 10,17 | 20,21 | 16 | 13 | 14 | 29 | 23 | 10 | 13 | 13 | 14 | 13 | 11 | 14 | 25 | 18 | 30 | 15 | 18 | 11 | 23 | 37 | 11 | 11 | 12 | 14 | 18 | 17 | 16 | 15 | 20 | 21 | 13 | 8 | 11 | 1 | 2 | 2 |
| S482 | Korean | 35,37 | 13,13 | 14,19 | 20,25 | 15 | 10 | 11 | 27 | 23 | 10 | 14 | 12 | 15 | 11 | 14 | 13 | 23 | 20 | 33 | 15 | 18 | 10 | 23 | 36 | 11 | 11 | 13 | 14 | 18 | 17 | 16 | 14 | 20 | 20 | 12 | 8 | 11 | 1 | 1 | 1 |
| S483 | Korean | 35,40 | 15,16 | 10,17 | 20,21 | 16 | 12 | 14 | 29 | 22 | 10 | 13 | 13 | 14 | 13 | 12 | 13 | 25 | 18 | 28 | 15 | 17 | 11 | 23 | 42 | 11 | 11 | 12 | 14 | 17.2 | 18 | 16 | 15 | 20 | 21 | 13 | 8 | 11 | 1 | 2 | 2 |
| S484 | Korean | 36,40 | 13,14 | 12,16 | 21,24 | 15 | 12 | 12 | 30 | 23 | 11 | 12 | 12 | 15 | 10 | 12 | 13 | 25 | 19 | 34 | 15 | 19 | 9 | 23 | 35 | 12 | 11 | 12 | 12 | 16 | 19 | 16 | 14 | 22 | 20 | 11 | 8 | 12 | 1 | 2 | 2 |
| S485 | Korean | 35,36,37 | 14,15 | 13,18 | 20,23,24 | 15 | 10 | 12 | 29 | 23 | 10 | 15 | 11 | 15 | 12 | 13 | 13 | 23 | 20 | 32 | 15 | 17 | 9 | 23 | 35 | 13 | 9 | 12 | 14 | 17 | 19 | 16 | 14 | 19 | 20 | 11 | 8 | 12 | 1 | 1 | 1 |
| S486 | Korean | 40,40 | 13,15 | 12,18 | 22,23 | 15 | 12 | 12 | 29 | 24 | 10 | 13 | 12 | 15 | 9 | 10 | 13 | 23 | 19 | 33 | 16 | 15 | 10 | 26 | 37 | 12 | 12 | 15 | 15 | 14 | 16 | 16 | 16 | 20 | 21 | 11 | 9 | 13 | 1 | 2 | 2 |
| S487 | Korean | 37,37 | 16,17 | 14,19 | 23,23 | 17 | 13 | 12 | 27 | 25 | 10 | 13 | 12 | 14 | 10 | 12 | 12 | 24 | 19 | 27 | 14 | 17 | 10 | 26 | 41 | 12 | 10 | 12 | 16 | 19.3 | 17 | 16 | 15 | 21 | 22 | 10 | 8 | 12 | 1 | 2 | 2 |
| S488 | Korean | 35,39 | 14,14 | 10,19 | 21,22 | 15 | 12 | 13 | 29 | 22 | 10 | 14 | 13 | 14 | 13 | 11 | 12 | 25 | 18 | 30 | 15 | 18 | 11 | 21 | 38 | 11 | 11 | 12 | 14 | 19 | 18 | 16 | 15 | 20 | 21 | 12 | 8 | 12 | 1 | 2 | 2 |
| S489 | Korean | 38,38 | 13,13 | 15,16 | 24,24 | 15 | 12 | 12 | 28 | 24 | 10 | 10 | 12 | 14 | 10 | 12 | 11 | 27 | 19 | 31 | 15 | 21 | 11 | 28 | 34 | 11 | 12 | 11 | 14 | 21 | 17 | 15 | 16 | 24 | 20 | 9 | 8 | 11 | 2 | 2 | 2 |
| S490 | Korean | 35,37 | 12,15 | 13,20 | 21,23 | 14 | 10 | 12 | 28 | 23 | 11 | 14 | 12 | 15 | 11 | 11 | 11 | 23 | 20 | 34 | 15 | 16 | 9 | 23 | 35 | 11 | 11 | 12 | 13 | 17 | 20 | 16 | 14 | 22 | 20 | 11 | 8 | 12 | 1 | 1 | 1 |
| S491 | Korean | 35,37 | 13,14 | 13,19 | 20,24 | 14 | 10 | 12 | 28 | 24 | 10 | 14 | 12 | 15 | 11 | 13 | 12 | 23 | 20 | 32 | 15 | 18 | 9 | 24 | 35 | 10 | 11 | 12 | 14 | 18 | 20 | 16 | 14 | 18 | 20 | 11 | 8 | 12 | 1 | 1 | 1 |
| S492 | Korean | 38,38 | 14,15 | 11,19 | 22,24 | 15 | 12 | 12 | 29 | 23 | 10 | 12 | 12 | 15 | 10 | 11 | 13 | 24 | 19 | 33 | 15 | 18 | 11 | 20 | 38 | 13 | 11 | 14 | 15 | 18 | 20 | 16 | 14 | 24 | 21 | 11 | 8 | 11 | 1 | 2 | 2 |
| S493 | Korean | 35,40 | 15,16 | 10,19 | 20,21 | 16 | 12 | 14 | 29 | 23 | 11 | 13 | 13 | 14 | 13 | 12 | 14 | 25 | 18 | 30 | 15 | 18 | 11 | 23 | 41 | 11 | 11 | 12 | 15 | 17 | 15 | 16 | 15 | 20 | 21 | 12 | 8 | 11 | 1 | 2 | 2 |
| S494 | Korean | 37,38 | 14,14 | 11,11 | 19,20 | 17 | 12 | 12 | 27 | 23 | 11 | 11 | 13 | 14 | 10 | 12 | 14 | 26 | 22 | 29 | 16 | 17 | 10 | 24 | 39 | 11 | 12 | 14 | 17 | 16 | 18 | 17 | 15 | 22 | 21 | 10 | 8 | 11 | 2 | 2 | 2 |
| S495 | Korean | 35,40 | 14,16 | 10,19 | 19,20 | 16 | 12 | 14 | 29 | 22 | 10 | 13 | 13 | 14 | 13 | 12 | 14 | 25 | 18 | 30 | 15 | 17 | 11 | 23 | 40 | 11 | 11 | 13 | 14 | 17 | 19 | 16 | 16 | 20 | 21 | 13 | 8 | 11 | 1 | 2 | 2 |
| S496 | Korean | 36,40 | 14,16 | 10,17 | 19,20 | 15 | 12 | 14 | 29 | 23 | 10 | 13 | 13 | 14 | 13 | 12 | 14 | 25 | 18 | 31 | 15 | 17 | 12 | 23 | 39 | 11 | 11 | 12 | 14 | 18 | 17 | 16 | 15 | 21 | 22 | 13 | 8 | 11 | 1 | 2 | 2 |
| S497 | Korean | 37,39 | 14,17 | 10,18 | 20,20 | 16 | 12 | 14 | 29 | 23 | 10 | 11 | 14 | 14 | 10 | 11 | 13 | 26 | 21 | 32 | 15 | 18 | 11 | 25 | 37 | 11 | 12 | 13 | 16 | 15 | 17 | 17 | 15 | 22 | 21 | 9 | 7 | 11 | 2 | 2 | 2 |
| S498 | Korean | 36,39 | 14,14 | 11,11 | 21,22 | 16 | 12 | 13 | 28 | 22 | 10 | 14 | 12 | 15 | 10 | 11 | 12 | 26 | 18 | 32 | 16 | 16 | 9 | 23 | 39 | 12 | 11 | 14 | 15 | 17 | 19 | 16 | 15 | 22 | 21 | 10 | 8 | 12 | 1 | 2 | 2 |
| S499 | Korean | 36,39 | 12,16 | 12,18 | 21,22 | 17 | 12 | 12 | 28 | 24 | 10 | 13 | 12 | 14 | 10 | 13 | 13 | 24 | 19 | 32 | 15 | 18 | 10 | 24 | 40 | 12 | 11 | 13 | 16 | 17 | 17 | 15 | 15 | 21 | 21 | 10 | 8 | 11 | 1 | 2 | 2 |
| S500 | Korean | 38,38 | 13,13 | 13,18 | 19,24 | 14 | 10 | 12 | 29 | 23 | 10 | 14 | 12 | 15 | 11 | 12 | 12 | 23 | 20 | 32 | 16 | 18 | 9 | 23 | 34 | 10 | 11 | 12 | 14 | 17 | 23 | 16 | 14 | 18 | 20 | 11 | 8 | 12 | 1 | 1 | 1 |
| S501 | Korean | 38,39 | 12,14 | 12,19 | 20,23 | 15 | 12 | 14 | 30 | 24 | 9 | 13 | 14 | 14 | 10 | 12 | 13 | 26 | 18 | 29 | 15 | 15 | 10 | 23 | 37 | 11 | 11 | 12 | 17 | 19 | 18 | 15 | 15 | 21 | 21 | 11 | 8 | 12 | 1 | 2 | 2 |
| S502 | Korean | 35,38 | 13,13 | 13,19 | 20,23 | 15 | 10 | 12 | 27 | 24 | 10 | 14 | 12 | 14 | 11 | 11 | 13 | 23 | 20 | 32 | 15 | 18 | 9 | 23 | 36 | 11 | 11 | 12 | 14 | 20 | 17 | 16 | 14 | 18 | 20 | 11 | 8 | 12 | 1 | 1 | 1 |
| S503 | Korean | 34,40 | 13,13 | 12,19 | 21,24 | 17 | 10 | 12 | 28 | 25 | 10 | 14 | 12 | 15 | 11 | 12 | 13 | 23 | 20 | 29 | 15 | 15 | 10 | 24 | 39 | 12 | 12 | 12 | 15 | 17 | 17 | 16 | 14 | 21 | 20 | 13 | 8 | 12 | 1 | 1 | 2 |
| S504 | Korean | 36,36 | 16,16 | 12,16 | 21,21 | 15 | 12 | 12 | 28 | 23 | 10 | 12 | 12 | 14 | 10 | 12 | 13 | 24 | 19 | 33 | 15 | 16 | 9 | 23 | 36 | 12 | 11 | 12 | 15 | 16 | 19 | 16 | 14 | 25 | 19 | 11 | 8 | 12 | 1 | 2 | 2 |
| S505 | Korean | 36,39 | 14,14 | 12,16 | 21,24 | 15 | 12 | 12 | 29 | 23 | 11 | 12 | 12 | 15 | 10 | 12 | 13 | 25 | 19 | 34 | 15 | 19 | 9 | 23 | 35 | 12 | 11 | 12 | 12 | 16 | 21 | 16 | 14 | 22 | 20 | 11 | 8 | 12 | 1 | 2 | 2 |
| S506 | Korean | 35,39 | 13,13 | 12,18 | 21,23 | 17 | 10 | 12 | 28 | 25 | 10 | 14 | 12 | 15 | 11 | 12 | 13 | 23 | 20 | 29 | 15 | 15 | 10 | 25 | 39 | 12 | 11 | 12 | 16 | 18 | 19 | 16 | 14 | 21 | 20 | 12 | 8 | 12 | 1 | 1 | 2 |
| S507 | Korean | 38,38 | 15,15 | 14,21 | 20,20 | 13 | 12 | 14 | 31 | 25 | 9 | 14 | 14 | 14 | 12 | 12 | 14 | 28 | 19 | 33 | 16 | 16 | 10 | 24 | 39.2 | 10 | 13 | 12 | 18 | 21 | 20 | 15 | 16 | 22 | 22 | 10 | 8 | 10 | 2 | 2 | 2 |
| S508 | Korean | 35,38 | 14,16 | 10,18 | 21,21 | 15 | 12 | 14 | 30 | 22 | 10 | 13 | 13 | 14 | 13 | 12 | 12 | 24 | 18 | 30 | 15 | 19 | 11 | 21 | 41 | 11 | 11 | 13 | 14 | 19 | 18 | 16 | 15 | 21 | 20 | 12 | 8 | 12 | 1 | 2 | 2 |
| S509 | Korean | 38,38 | 16,16 | 12,13 | 21,21 | 14 | 12 | 13 | 29 | 23 | 10 | 15 | 14 | 14 | 10 | 11 | 12 | 29 | 19 | 27 | 16 | 16 | 11 | 23 | 37 | 12 | 12 | 12 | 14 | 17 | 18 | 15 | 14 | 20 | 21 | 12 | 8 | 12 | 2 | 2 | 2 |
| S510 | Korean | 35,40 | 14,15 | 10,19 | 20,21 | 16 | 12 | 14 | 29 | 22 | 10 | 13 | 13 | 14 | 13 | 12 | 14 | 25 | 18 | 30 | 15 | 17 | 11 | 23 | 41 | 11 | 11 | 13 | 14 | 17 | 19 | 16 | 16 | 20 | 21 | 13 | 8 | 11 | 1 | 2 | 2 |
| S511 | Korean | 37,41 | 14,15 | 13,17 | 21,23 | 16 | 12 | 12 | 29 | 25 | 10 | 13 | 12 | 15 | 10 | 12 | 13 | 23 | 20 | 35 | 15 | 18 | 9 | 24 | 39 | 12 | 11 | 12 | 14 | 19 | 21 | 17 | 15 | 19 | 20 | 12 | 8 | 12 | 1 | 2 | 2 |
| S512 | Korean | 38,38 | 16,16 | 11,18 | 20,21 | 16 | 13 | 13 | 30 | 23 | 10 | 11 | 14 | 14 | 10 | 11 | 12 | 26 | 21 | 30 | 15 | 16 | 11 | 25 | 39 | 12 | 12 | 13 | 17 | 16 | 18 | 17 | 15 | 24 | 21 | 9 | 8 | 11 | 2 | 2 | 2 |
| S513 | Korean | 37,38 | 15,16 | 14,18 | 23,23 | 17 | 12 | 12 | 27 | 25 | 11 | 13 | 12 | 14 | 10 | 11 | 12 | 24 | 19 | 27 | 14 | 18 | 10 | 25 | 38 | 12 | 10 | 12 | 16 | 19.3 | 18 | 16 | 15 | 22 | 21 | 10 | 8 | 12 | 1 | 2 | 2 |
| S514 | Korean | 35,39 | 14,15 | 9,19 | 21,22 | 15 | 12 | 13 | 29 | 22 | 10 | 13 | 13 | 14 | 13 | 12 | 12 | 25 | 18 | 30 | 15 | 18 | 11 | 19 | 39 | 11 | 10 | 12 | 15 | 21 | 19 | 16 | 15 | 21 | 20 | 12 | 8 | 12 | 1 | 2 | 2 |
| S515 | Korean | 37,41 | 14,15 | 11,11 | 20,22 | 16 | 12 | 13 | 28 | 22 | 10 | 14 | 12 | 15 | 10 | 12 | 12 | 26 | 18 | 32 | 16 | 17 | 9 | 23 | 39 | 12 | 11 | 13 | 15 | 17 | 18 | 16 | 15 | 21 | 20 | 10 | 8 | 12 | 1 | 2 | 2 |
| S516 | Korean | 37,37 | 16,16 | 12,13 | 23,23 | 14 | 12 | 14 | 30 | 23 | 10 | 15 | 13 | 14 | 10 | 11 | 12 | 27 | 19 | 26 | 15 | 15 | 11 | 22 | 36 | 12 | 12 | 13 | 14 | 17 | 16 | 15 | 14 | 21 | 21 | 13 | 8 | 12 | 2 | 2 | 2 |
| S517 | Korean | 35,39 | 15,16 | 10,17 | 19,23 | 17 | 12 | 14 | 30 | 23 | 11 | 13 | 13 | 14 | 12 | 12 | 14 | 24 | 18 | 29 | 15 | 16 | 11 | 24 | 39 | 11 | 11 | 12 | 14 | 21 | 21 | 16 | 15 | 18 | 21 | 12 | 8 | 12 | 1 | 2 | 2 |
| S518 | Korean | 36,38 | 15,16 | 12,18 | 23,23 | 16 | 12 | 12 | 27 | 24 | 11 | 13 | 13 | 14 | 10 | 11 | 12 | 24 | 20 | 26 | 14 | 17 | 10 | 26 | 38 | 12 | 11 | 13 | 17 | 18 | 16 | 16 | 15 | 18 | 21 | 9 | 8 | 11 | 1 | 2 | 2 |
| S519 | Korean | 34,39 | 14,16 | 10,21 | 22,23 | 15 | 12 | 14 | 30 | 24 | 11 | 13 | 14 | 14 | 13 | 11 | 14 | 25 | 17 | 28 | 15 | 17 | 11 | 22 | 38 | 12 | 11 | 12 | 12 | 18 | 19 | 16 | 15 | 24 | 20 | 12 | 8 | 12 | 1 | 2 | 2 |
| S520 | Korean | 36,40 | 13,14,16 | 9,11 | 21,22,23 | 15 | 12 | 13 | 28 | 21 | 10 | 15 | 12 | 15 | 11 | 12 | 12 | 27 | 19 | 32 | 16 | 16 | 9 | 23 | 38 | 12 | 12 | 14 | 15 | 18 | 19 | 16 | 15 | 24 | 20 | 10 | 8 | 12 | 1 | 2 | 2 |
| S521 | Korean | 35,39 | 13,15 | 10,20 | 21,22 | 15 | 12 | 14 | 30 | 22 | 10 | 13 | 13 | 14 | 13 | 12 | 12 | 25 | 18 | 29 | 15 | 18 | 10 | 21 | 38 | 11 | 11 | 11 | 14 | 19 | 17 | 16 | 15 | 21 | 21 | 12 | 8 | 12 | 1 | 2 | 2 |
| S522 | Korean | 35,40 | 15,16 | 10,17 | 20,21 | 15 | 12 | 14 | 29 | 23 | 10 | 13 | 13 | 14 | 13 | 12 | 13 | 25 | 18 | 30 | 15 | 18 | 11 | 23 | 41 | 11 | 11 | 12 | 14 | 18 | 17 | 16 | 15 | 21 | 22 | 13 | 8 | 11 | 1 | 2 | 2 |
| S523 | Korean | 37,38 | 12,13 | 15,20 | 20,21 | 17 | 13 | 12 | 29 | 24 | 10 | 13 | 12 | 15 | 10 | 11 | 13 | 24 | 20 | 31 | 15 | 17 | 11 | 23 | 39 | 13 | 11 | 14 | 14 | 19 | 17 | 17 | 15 | 20 | 21 | 11 | 8 | 12 | 1 | 2 | 2 |
| S524 | Korean | 38,38 | 15,15 | 15,22 | 21,21 | 13 | 12 | 14 | 30 | 24 | 9 | 14 | 14 | 14 | 12 | 11 | 13 | 27 | 19 | 31 | 16 | 17 | 10 | 24 | 37.2 | 10 | 12 | 11 | 19 | 18 | 20 | 15 | 16 | 22 | 22 | 10 | 8 | 10 | 2 | 2 | 2 |
| S525 | Korean | 36,40 | 12,12 | 14,21 | 21,21 | 17 | 13 | 12 | 28 | 24 | 11 | 13 | 12 | 14 | 10 | 11 | 14 | 24 | 19 | 29 | 15 | 16 | 11 | 23 | 39 | 12 | 11 | 14 | 14 | 19 | 19 | 17 | 15 | 21 | 21 | 11 | 8 | 12 | 1 | 2 | 2 |
| S526 | Korean | 35,38 | 14,17 | 10,17 | 21,21 | 15 | 12 | 14 | 31 | 22 | 10 | 13 | 13 | 14 | 13 | 12 | 12 | 24 | 18 | 29 | 15 | 18 | 11 | 21 | 39 | 11 | 11 | 12 | 14 | 19 | 18 | 16 | 15 | 20 | 20 | 12 | 8 | 11 | 1 | 2 | 2 |
| S527 | Korean | 36,38 | 12,13 | 14,22 | 21,21 | 17 | 13 | 12 | 30 | 24 | 10 | 13 | 12 | 15 | 10 | 11 | 13 | 24 | 20 | 32 | 15 | 16 | 11 | 23 | 38 | 12 | 11 | 13 | 14 | 18 | 18 | 17 | 15 | 20 | 21 | 11 | 8 | 12 | 1 | 2 | 2 |
| S528 | Korean | 35,39 | 15,16 | 10,17 | 19,23 | 17 | 12 | 15 | 31 | 23 | 11 | 13 | 13 | 14 | 12 | 12 | 14 | 24 | 18 | 29 | 15 | 16 | 11 | 24 | 39 | 11 | 11 | 12 | 14 | 21 | 21 | 16 | 15 | 18 | 21 | 12 | 8 | 12 | 1 | 2 | 2 |
| S529 | Korean | 36,40 | 15,16 | 10,17 | 20,21 | 15 | 12 | 14 | 29 | 23 | 10 | 13 | 13 | 14 | 13 | 12 | 13 | 25 | 18 | 30 | 15 | 18 | 11 | 23 | 41 | 11 | 11 | 12 | 14 | 18 | 17 | 16 | 15 | 20 | 22 | 13 | 8 | 11 | 1 | 2 | 2 |
| S530 | Korean | 36,39 | 13,14 | 12,16 | 21,23 | 15 | 12 | 12 | 29 | 23 | 11 | 12 | 12 | 15 | 10 | 12 | 13 | 25 | 18 | 34 | 15 | 19 | 9 | 23 | 35 | 12 | 11 | 12 | 12 | 16 | 20 | 16 | 14 | 23 | 20 | 11 | 8 | 12 | 1 | 2 | 2 |
| S531 | Korean | 36,40 | 14,16 | 10,17 | 19,20 | 15 | 12 | 14 | 29 | 23 | 10 | 13 | 13 | 14 | 13 | 12 | 14 | 25 | 18 | 31 | 15 | 17 | 11 | 23 | 40 | 11 | 11 | 12 | 14 | 18 | 17 | 16 | 15 | 21 | 22 | 13 | 8 | 11 | 1 | 2 | 2 |
| S532 | Korean | 36,39 | 14,17 | 11,17 | 18,23 | 15 | 12 | 12 | 29 | 24 | 10 | 13 | 12 | 14 | 10 | 12 | 13 | 24 | 19 | 31 | 14 | 19 | 10 | 24 | 43 | 13 | 11 | 15 | 16 | 17 | 17 | 16 | 15 | 21 | 21 | 11 | 8 | 12 | 1 | 2 | 2 |
| S533 | Korean | 35,40 | 15,16 | 10,17 | 20,21 | 16 | 12 | 14 | 29 | 22 | 10 | 13 | 13 | 14 | 13 | 12 | 13 | 25 | 18 | 28 | 15 | 18 | 11 | 23 | 42 | 11 | 11 | 12 | 14 | 17.2 | 18 | 16 | 15 | 20 | 21 | 13 | 8 | 11 | 1 | 2 | 2 |
| S534 | Korean | 35,36,37 | 14,16 | 12,19 | 21,22 | 17 | 12 | 13 | 30 | 24 | 10 | 13 | 12 | 14 | 10 | 12 | 12 | 25 | 19 | 31 | 14 | 18 | 10 | 24 | 38 | 12 | 11 | 14 | 16 | 19 | 21 | 16 | 15 | 23 | 23 | 10 | 8 | 10 | 1 | 2 | 2 |
| S535 | Korean | 35,40 | 15,16 | 10,18 | 20,21 | 15 | 12 | 14 | 29 | 23 | 10 | 14 | 13 | 14 | 13 | 12 | 14 | 25 | 18 | 30 | 15 | 17 | 11 | 24 | 39 | 11 | 11 | 11 | 14 | 18 | 18 | 16 | 15 | 23 | 22 | 13 | 8 | 11 | 1 | 2 | 2 |
| S536 | Korean | 35,40 | 15,16 | 10,19 | 21,23 | 16 | 12 | 14 | 29 | 23 | 10 | 13 | 13 | 14 | 13 | 12 | 14 | 25 | 18 | 31 | 15 | 17 | 10 | 23 | 41 | 11 | 11 | 12 | 14 | 18 | 17 | 16 | 15 | 21 | 22 | 13 | 8 | 11 | 1 | 2 | 2 |
| S537 | Korean | 36,39 | 15,15 | 11,19 | 20,21 | 15 | 12 | 14 | 29 | 24 | 10 | 13 | 13 | 14 | 13 | 12 | 14 | 25 | 18 | 31 | 15 | 16 | 12 | 23 | 39 | 11 | 11 | 12 | 14 | 18 | 18 | 16 | 15 | 22 | 21 | 13 | 8 | 12 | 1 | 2 | 2 |
| S538 | Korean | 34,38 | 13,14 | 12,19 | 20,25 | 14 | 10 | 12 | 28 | 24 | 10 | 14 | 12 | 15 | 11 | 11 | 11 | 23 | 20 | 32 | 15 | 19 | 9 | 23 | 36 | 11 | 11 | 12 | 14 | 19 | 18 | 16 | 14 | 22 | 22 | 11 | 8 | 13 | 1 | 1 | 1 |
| S539 | Korean | 35,38 | 14,16 | 12,20 | 22,23 | 17 | 13 | 12 | 28 | 25 | 10 | 13 | 12 | 14 | 9 | 14 | 12 | 24 | 19 | 33 | 14 | 17 | 10 | 25 | 38 | 13 | 12 | 13 | 17 | 17 | 20 | 16 | 15 | 20 | 21 | 10 | 8 | 10 | 1 | 2 | 2 |
| S540 | Korean | 38,38 | 16,16 | 12,17 | 23,23 | 15 | 13 | 13 | 29 | 23 | 10 | 11 | 14 | 14 | 10 | 10 | 14 | 26 | 21 | 28 | 15 | 16 | 10 | 24 | 37 | 11 | 14 | 12 | 18 | 16 | 17 | 17 | 15 | 20 | 21 | 9 | 8 | 12 | 2 | 2 | 2 |
| S541 | Korean | 35,42 | 15,16 | 10,18 | 20,21 | 15 | 12 | 14 | 30 | 23 | 10 | 13 | 14 | 14 | 13 | 11 | 13 | 25 | 18 | 32 | 15 | 17 | 11 | 23 | 39 | 12 | 11 | 14 | 16 | 18 | 18 | 16 | 15 | 21 | 21 | 14 | 8 | 11 | 1 | 2 | 2 |
| S542 | Korean | 35,39 | 13,15 | 10,20 | 21,22 | 15 | 12 | 14 | 30 | 22 | 10 | 13 | 13 | 14 | 13 | 12 | 12 | 25 | 18 | 29 | 15 | 18 | 10 | 21 | 38 | 11 | 11 | 11 | 14 | 18 | 18 | 16 | 15 | 21 | 22 | 12 | 8 | 12 | 1 | 2 | 2 |
| S543 | Korean | 35,39 | 13,16 | 12,18 | 18,20 | 16 | 13 | 14 | 30 | 23 | 9 | 11 | 15 | 15 | 10 | 11 | 14 | 25 | 21 | 29 | 15 | 16 | 10 | 25 | 38 | 11 | 11 | 11 | 19 | 16 | 19 | 17 | 15 | 21 | 21 | 8 | 8 | 11 | 2 | 2 | 2 |
| S544 | Korean | 36,38 | 12,14 | 15,23 | 20,21 | 17 | 13 | 12 | 30 | 24 | 10 | 13 | 12 | 15 | 10 | 11 | 13 | 23 | 20 | 31 | 16 | 16 | 11 | 23 | 38 | 12 | 11 | 13 | 14 | 19 | 17 | 17 | 15 | 22 | 21 | 11 | 8 | 12 | 1 | 2 | 2 |
| S545 | Korean | 37,37 | 16,16 | 10,18 | 20,20 | 16 | 13 | 13 | 30 | 23 | 10 | 11 | 15 | 14 | 10 | 12 | 13 | 25 | 20 | 29 | 15 | 16 | 11 | 24 | 39 | 11 | 12 | 12 | 19 | 17 | 19 | 17 | 15 | 22 | 21 | 8 | 8 | 11 | 2 | 2 | 2 |
| S546 | Korean | 36,37 | 15,16 | 11,18 | 19,20 | 15 | 13 | 14 | 30 | 23 | 10 | 11 | 14 | 14 | 10 | 11 | 15 | 25 | 21 | 30 | 15 | 19 | 11 | 27 | 40 | 11 | 12 | 12 | 18 | 20 | 18 | 17 | 15 | 20 | 21 | 8 | 8 | 11 | 2 | 2 | 2 |
| S547 | Korean | 35,40 | 15,16 | 11,18 | 22,22 | 15 | 12 | 13 | 28 | 23 | 11 | 13 | 13 | 14 | 14 | 13 | 12 | 25 | 18 | 30 | 15 | 16 | 10 | 21 | 36 | 12 | 11 | 12 | 14 | 18 | 17 | 16 | 15 | 20 | 20 | 12 | 8 | 13 | 1 | 2 | 2 |
| S548 | Korean | 36,41 | 16,17 | 10,18 | 20,21 | 17 | 12 | 14 | 29 | 23 | 10 | 13 | 13 | 14 | 13 | 12 | 14 | 25 | 18 | 30 | 15 | 18 | 11 | 23 | 39 | 11 | 11 | 11 | 14 | 18 | 17 | 16 | 15 | 23 | 21 | 13 | 8 | 11 | 1 | 2 | 2 |
| S549 | Korean | 37,39 | 12,14 | 14,19 | 20,22 | 16 | 13 | 12 | 29 | 24 | 10 | 13 | 12 | 14 | 11 | 11 | 12 | 24 | 20 | 33 | 16 | 18 | 11 | 24 | 40 | 11 | 11 | 13 | 14 | 18 | 20 | 17 | 15 | 19 | 21 | 11 | 8 | 12 | 1 | 2 | 2 |
| S550 | Korean | 36,40 | 13,14 | 11,20 | 22,23 | 17 | 12 | 12 | 28 | 22 | 10 | 13 | 12 | 14 | 10 | 12 | 13 | 25 | 18 | 31 | 15 | 18 | 9 | 24 | 39 | 12 | 11 | 13 | 15 | 18 | 18 | 16 | 15 | 21 | 22 | 10 | 8 | 12 | 1 | 2 | 2 |
| S551 | Korean | 34,38 | 13,13 | 13,19 | 20,25 | 14 | 10 | 12 | 28 | 24 | 10 | 14 | 12 | 15 | 11 | 12 | 11 | 23 | 20 | 32 | 15 | 19 | 9 | 24 | 36 | 11 | 11 | 13 | 14 | 18 | 19 | 16 | 14 | 22 | 22 | 11 | 8 | 13 | 1 | 1 | 1 |
| S552 | Korean | 35,40 | 13,13 | 12,17 | 20,25 | 15 | 12 | 12 | 28 | 23 | 10 | 12 | 12 | 15 | 10 | 12 | 13 | 24 | 19 | 31 | 15 | 19 | 9 | 22 | 39 | 13 | 12 | 13 | 16 | 16 | 18 | 16 | 14 | 21 | 22 | 10 | 9 | 13 | 1 | 2 | 2 |
| S553 | Korean | 36,40 | 14,16 | 10,19 | 21,22 | 16 | 12 | 14 | 29 | 23 | 10 | 13 | 13 | 14 | 13 | 12 | 14 | 25 | 18 | 32 | 15 | 17 | 11 | 23 | 43 | 11 | 11 | 12 | 14 | 18 | 17 | 16 | 15 | 21 | 21 | 13 | 8 | 11 | 1 | 2 | 2 |
| S554 | Korean | 36,39 | 12,16 | 12,17 | 21,23 | 17 | 12 | 12 | 29 | 24 | 10 | 13 | 12 | 14 | 10 | 12 | 13 | 24 | 19 | 32 | 13 | 17 | 10 | 20 | 42 | 12 | 11 | 13 | 16 | 17 | 17 | 15 | 15 | 19 | 22 | 10 | 8 | 12 | 1 | 2 | 2 |
| S555 | Korean | 34,39 | 15,15 | 10,19 | 20,21 | 16 | 12 | 12 | 27 | 23 | 10 | 13 | 13 | 14 | 13 | 12 | 12 | 25 | 18 | 30 | 15 | 17 | 10 | 23 | 40 | 11 | 11 | 12 | 14 | 16 | 17 | 16 | 15 | 20 | 21 | 13 | 8 | 11 | 1 | 2 | 2 |
| S556 | Korean | 39,41 | 12,14 | 12,18 | 21,24 | 15 | 12 | 14 | 30 | 24 | 9 | 13 | 14 | 14 | 10 | 12 | 14 | 23 | 18 | 29 | 15 | 15 | 11 | 23 | 38 | 11 | 11 | 12 | 17 | 20 | 18 | 15 | 15 | 21 | 21 | 11 | 8 | 12 | 1 | 2 | 2 |
| S557 | Korean | 35,35 | 12,14 | 10,18 | 21,21 | 16 | 12 | 14 | 29 | 23 | 10 | 13 | 13 | 14 | 13 | 12 | 13 | 25 | 18 | 30 | 15 | 17 | 11 | 23 | 41 | 11 | 11 | 13 | 14 | 18 | 18 | 16 | 15 | 21 | 21 | 13 | 8 | 11 | 1 | 2 | 2 |
| S558 | Korean | 36,38 | 15,17 | 12,18 | 22,24 | 15 | 12 | 12 | 29 | 25 | 10 | 13 | 12 | 14 | 10 | 11 | 12 | 24 | 19 | 32 | 15 | 19 | 10 | 23 | 39 | 13 | 11 | 12 | 16 | 17 | 18 | 16 | 15 | 19 | 22 | 10 | 8 | 11 | 1 | 2 | 2 |
| S559 | Korean | 37,38 | 12,14 | 15,22 | 20,21 | 17 | 13 | 12 | 30 | 24 | 10 | 13 | 12 | 15 | 10 | 11 | 13 | 23 | 20 | 31 | 15 | 16 | 11 | 23 | 38 | 11 | 11 | 13 | 14 | 18 | 17 | 17 | 15 | 22 | 21 | 11 | 8 | 12 | 1 | 2 | 2 |
| S560 | Korean | 35,40 | 15,16 | 10,19 | 21,22 | 16 | 12 | 14 | 29 | 23 | 10 | 13 | 13 | 14 | 13 | 12 | 14 | 25 | 18 | 32 | 15 | 16 | 11 | 23 | 40 | 11 | 11 | 12 | 14 | 18 | 17 | 16 | 15 | 21 | 21 | 13 | 8 | 11 | 1 | 2 | 2 |
| S561 | Korean | 34,38 | 13,14 | 13,20 | 20,25 | 14 | 10 | 12 | 28 | 24 | 10 | 14 | 12 | 15 | 11 | 12 | 11 | 23 | 20 | 32 | 15 | 19 | 9 | 23 | 36 | 11 | 11 | 12 | 14 | 18 | 19 | 16 | 14 | 23 | 23 | 11 | 8 | 13 | 1 | 1 | 1 |
| S562 | Korean | 35,39 | 15,16 | 10,19 | 21,22 | 16 | 12 | 14 | 29 | 23 | 10 | 13 | 13 | 14 | 13 | 12 | 14 | 25 | 18 | 31 | 16 | 18 | 11 | 23 | 41 | 11 | 11 | 12 | 14 | 18 | 17 | 16 | 15 | 23 | 21 | 13 | 8 | 11 | 1 | 2 | 2 |
| S563 | Korean | 37,37 | 16,16 | 11,16 | 20,20 | 16 | 13 | 13 | 31 | 23 | 10 | 11 | 15 | 14 | 10 | 12 | 14 | 26 | 21 | 30 | 16 | 17 | 11 | 24 | 38 | 11 | 12 | 12 | 17 | 19 | 20 | 17 | 15 | 23 | 21 | 9 | 8 | 11 | 2 | 2 | 2 |
| S564 | Korean | 35,36 | 9,16 | 12,18 | 19,21 | 16 | 13 | 13 | 29 | 22 | 10 | 11 | 14 | 14 | 10 | 12 | 13 | 28 | 22 | 31 | 15 | 17 | 11 | 24 | 36 | 11 | 12 | 14 | 17 | 16 | 18 | 16 | 15 | 23 | 22 | 9 | 8 | 11 | 2 | 2 | 2 |
| S565 | Korean | 36,38 | 12,13 | 16,21 | 20,21 | 17 | 13 | 12 | 29 | 24 | 10 | 13 | 12 | 14 | 10 | 11 | 14 | 24 | 20 | 31 | 15 | 16 | 11 | 23 | 37 | 12 | 11 | 13 | 14 | 21 | 19 | 17 | 15 | 20 | 21 | 11 | 8 | 12 | 1 | 2 | 2 |
| S566 | Korean | 35,38 | 14,16 | 12,12 | 21,23 | 15 | 12 | 12 | 28 | 23 | 10 | 12 | 12 | 15 | 12 | 12 | 13 | 23 | 20 | 34 | 16 | 19 | 10 | 21 | 37 | 12 | 11 | 13 | 16 | 19 | 18 | 16 | 14 | 24 | 23 | 11 | 8 | 11 | 1 | 2 | 2 |
| S567 | Korean | 38,38 | 18,18 | 13,13 | 23,23 | 14 | 12 | 14 | 30 | 23 | 10 | 15 | 13 | 14 | 10 | 11 | 12 | 27 | 19 | 27 | 15 | 15 | 11 | 23 | 37 | 12 | 12 | 13 | 14 | 17 | 19 | 15 | 14 | 20 | 21 | 12 | 8 | 13 | 2 | 2 | 2 |
| S568 | Korean | 38,39 | 14,15 | 10,20 | 19,20 | 16 | 12 | 15 | 30 | 24 | 10 | 11 | 15 | 14 | 10 | 12 | 14 | 27 | 21 | 32 | 15 | 16 | 11 | 25 | 42 | 11 | 12 | 13 | 18 | 15 | 17 | 17 | 15 | 24 | 21 | 9 | 8 | 11 | 2 | 2 | 2 |
| S569 | Korean | 37,39 | 12,13 | 15,19 | 20,21 | 17 | 13 | 12 | 27 | 23 | 10 | 13 | 12 | 14 | 10 | 12 | 13 | 24 | 20 | 31 | 15 | 16 | 11 | 23 | 38 | 12 | 12 | 13 | 13 | 20 | 17 | 17 | 15 | 22 | 21 | 11 | 8 | 12 | 1 | 2 | 2 |
| S570 | Korean | 36,41 | 15,17 | 10,18 | 20,21 | 15 | 12 | 13 | 28 | 23 | 10 | 13 | 13 | 14 | 13 | 12 | 12 | 25 | 18 | 30 | 15 | 17 | 11 | 23 | 40 | 11 | 11 | 12 | 15 | 17 | 18 | 16 | 15 | 20 | 22 | 12 | 8 | 11 | 1 | 2 | 2 |
| S571 | Korean | 37,38 | 14,15 | 12,17 | 21,23 | 17 | 12 | 13 | 28 | 23 | 10 | 14 | 13 | 14 | 10 | 12 | 12 | 24 | 20 | 34 | 15 | 18 | 10 | 25 | 36 | 12 | 11 | 12 | 16 | 19 | 19 | 16 | 17 | 18 | 22 | 11 | 8 | 11 | 1 | 2 | 2 |
| S572 | Korean | 35,41 | 14,18 | 10,19 | 20,21 | 16 | 12 | 14 | 29 | 23 | 11 | 13 | 13 | 14 | 13 | 12 | 15 | 25 | 18 | 31 | 15 | 17 | 11 | 24 | 42 | 11 | 11 | 13 | 14 | 19 | 17 | 16 | 15 | 20 | 21 | 12 | 8 | 11 | 1 | 2 | 2 |
| S573 | Korean | 36,36 | 15,15 | 11,13 | 22,22 | 14 | 12 | 13 | 29 | 23 | 10 | 14 | 13 | 14 | 10 | 11 | 11 | 26 | 19 | 26 | 15 | 15 | 11 | 22 | 36 | 12 | 12 | 12 | 15 | 17 | 19 | 15 | 14 | 25 | 23 | 10 | 8 | 12 | 2 | 2 | 2 |
| S574 | Korean | 37,39 | 15,15 | 14,17 | 23,24 | 18 | 12 | 12 | 28 | 25 | 11 | 13 | 12 | 14 | 10 | 12 | 12 | 24 | 20 | 27 | 14 | 16 | 10 | 23 | 38 | 12 | 11 | 12 | 16 | 20 | 16 | 15 | 15 | 20 | 22 | 10 | 8 | 11 | 1 | 2 | 2 |
| S575 | Korean | 35,39 | 14,14 | 12,16 | 20,22 | 15 | 12 | 12 | 30 | 23 | 10 | 12 | 12 | 14 | 10 | 13 | 12 | 24 | 19 | 32 | 15 | 16 | 9 | 22 | 37 | 12 | 10 | 12 | 16 | 18 | 18 | 16 | 14 | 23 | 20 | 12 | 8 | 11 | 1 | 2 | 2 |
| S576 | Korean | 35,40 | 16,16 | 10,10 | 20,21 | 15 | 13 | 14 | 29 | 23 | 10 | 13 | 13 | 14 | 13 | 11 | 14 | 25 | 18 | 30 | 15 | 18 | 11 | 23 | 37 | 11 | 11 | 12 | 14 | 18 | 17 | 16 | 15 | 20 | 21 | 13 | 8 | 11 | 1 | 2 | 2 |
| S577 | Korean | 37,40 | 13,15 | 12,13 | 21,22 | 16 | 12 | 11 | 27 | 23 | 10 | 14 | 13 | 14 | 10 | 11 | 11 | 24 | 18 | 33 | 16 | 15 | 10 | 24 | 37 | 10 | 12 | 12 | 15 | 19 | 14 | 15 | 16 | 22 | 18 | 11 | 8 | 11 | 1 | 2 | 2 |
| S578 | Korean | 36,41 | 14,15 | 11,11 | 20,22 | 15 | 12 | 13 | 28 | 22 | 10 | 14 | 12 | 15 | 10 | 12 | 12 | 26 | 19 | 31 | 16 | 17 | 9 | 23 | 39 | 12 | 11 | 14 | 15 | 18 | 18 | 16 | 15 | 21 | 21 | 10 | 8 | 12 | 1 | 2 | 2 |
| S579 | Korean | 35,39 | 15,16 | 10,19 | 21,22 | 15 | 12 | 14 | 29 | 23 | 10 | 13 | 13 | 14 | 14 | 12 | 12 | 24 | 18 | 31 | 15 | 17 | 11 | 23 | 41 | 11 | 11 | 12 | 14 | 17 | 17 | 16 | 15 | 20 | 21 | 13 | 8 | 11 | 1 | 2 | 2 |
| S580 | Korean | 34,39 | 13,14 | 13,13 | 21,22 | 15 | 12 | 12 | 29 | 22 | 10 | 14 | 13 | 14 | 10 | 11 | 12 | 25 | 18 | 30 | 15 | 15 | 11 | 23 | 38 | 10 | 12 | 13 | 14 | 20 | 18 | 15 | 14 | 19 | 21 | 11 | 8 | 11 | 1 | 2 | 2 |
| S581 | Korean | 35,39 | 15,16 | 10,19 | 21,22 | 16 | 12 | 14 | 29 | 24 | 10 | 13 | 13 | 14 | 13 | 13 | 14 | 25 | 18 | 30 | 15 | 17 | 12 | 23 | 40 | 11 | 11 | 12 | 14 | 18 | 17 | 16 | 15 | 21 | 21 | 13 | 8 | 11 | 1 | 2 | 2 |
| S582 | Korean | 38,40 | 13,14 | 12,17 | 21,23 | 15 | 12 | 14 | 30 | 24 | 10 | 13 | 13 | 14 | 10 | 11 | 14 | 27 | 18 | 30 | 15 | 15 | 12 | 23 | 41 | 11 | 11 | 12 | 18 | 20 | 17 | 15 | 15 | 21 | 21 | 11 | 8 | 12 | 1 | 2 | 2 |
| S583 | Korean | 39,39 | 14,14 | 15,19 | 21,21 | 14 | 12 | 14 | 30 | 24 | 9 | 14 | 14 | 14 | 12 | 12 | 14 | 27 | 19 | 30 | 17 | 17 | 10 | 24 | 37.2 | 10 | 12 | 13 | 19 | 20 | 18 | 15 | 16 | 21 | 20 | 9 | 8 | 10 | 2 | 2 | 2 |
| S584 | Korean | 37,38 | 12,14 | 15,20 | 20,20 | 16 | 13 | 12 | 29 | 24 | 10 | 13 | 12 | 14 | 10 | 11 | 12 | 24 | 20 | 30 | 15 | 18 | 11 | 23 | 39 | 12 | 11 | 13 | 14 | 18 | 17 | 17 | 15 | 21 | 21 | 11 | 8 | 13 | 1 | 2 | 2 |
| S585 | Korean | 36,39 | 12,13 | 14,22 | 20,21 | 15 | 13 | 12 | 28 | 24 | 10 | 13 | 12 | 14 | 10 | 11 | 14 | 24 | 20 | 33 | 16 | 16 | 11 | 22 | 38 | 12 | 11 | 13 | 14 | 20 | 20 | 17 | 15 | 20 | 22 | 11 | 8 | 12 | 1 | 2 | 2 |
| S586 | Korean | 36,39 | 15,15 | 14,17 | 23,25 | 15 | 10 | 12 | 28 | 24 | 10 | 14 | 12 | 15 | 11 | 12 | 12 | 23 | 20 | 32 | 14 | 16 | 9 | 24 | 36 | 13 | 11 | 12 | 14 | 16 | 19 | 16 | 14 | 20 | 21 | 11 | 8 | 12 | 1 | 1 | 1 |
| S587 | Korean | 37,37 | 14,17 | 10,20 | 19,20 | 15 | 12 | 14 | 29 | 24 | 10 | 11 | 15 | 14 | 10 | 13 | 13 | 27 | 21 | 32 | 15 | 15 | 10 | 26 | 40 | 11 | 12 | 12 | 17 | 15 | 18 | 17 | 15 | 23 | 22 | 9 | 8 | 11 | 2 | 2 | 2 |
| S588 | Korean | 35,39 | 14,14 | 9,18 | 22,22 | 15 | 12 | 14 | 32 | 22 | 10 | 13 | 13 | 14 | 13 | 12 | 12 | 25 | 18 | 30 | 15 | 18 | 10 | 19 | 37 | 11 | 11 | 12 | 14 | 20 | 18 | 16 | 15 | 21 | 20 | 12 | 8 | 12 | 1 | 2 | 2 |
| S589 | Korean | 34,38 | 13,14 | 13,19 | 20,24 | 15 | 10 | 12 | 28 | 24 | 10 | 14 | 12 | 15 | 11 | 12 | 11 | 23 | 20 | 32 | 15 | 19 | 9 | 23 | 35 | 11 | 11 | 12 | 13 | 18 | 18 | 16 | 14 | 22 | 22 | 11 | 8 | 13 | 1 | 1 | 1 |
| S590 | Korean | 40,40 | 14,15 | 12,12 | 19,20 | 16 | 15 | 13 | 29 | 25 | 10 | 11 | 13 | 14 | 10 | 10 | 13 | 26 | 22 | 27 | 15 | 19 | 10 | 25 | 39 | 11 | 12 | 12 | 15 | 17 | 18 | 16 | 16 | 20 | 21 | 10 | 8 | 11 | 2 | 2 | 2 |
| S591 | Korean | 37,38 | 12,13 | 15,21 | 20,21 | 17 | 13 | 12 | 30 | 24 | 10 | 13 | 12 | 15 | 10 | 11 | 13 | 24 | 20 | 30 | 15 | 16 | 11 | 23 | 42 | 12 | 11 | 13 | 14 | 19 | 18 | 17 | 15 | 21 | 21 | 11 | 8 | 12 | 1 | 2 | 2 |
| S592 | Korean | 35,37 | 14,17 | 11,18 | 20,21 | 16 | 13 | 13 | 29 | 23 | 11 | 11 | 14 | 14 | 10 | 12 | 13 | 26 | 21 | 30 | 15 | 16 | 11 | 24 | 38 | 12 | 13 | 13 | 17 | 16 | 18 | 17 | 15 | 21 | 21 | 9 | 8 | 11 | 2 | 2 | 2 |
| S593 | Korean | 36,39 | 15,16 | 10,19 | 21,22 | 16 | 12 | 14 | 29 | 23 | 10 | 13 | 13 | 14 | 13 | 12 | 13 | 25 | 18 | 31 | 15 | 17 | 11 | 23 | 40 | 12 | 11 | 12 | 14 | 18 | 17 | 16 | 15 | 21 | 21 | 12 | 8 | 12 | 1 | 2 | 2 |
| S594 | Korean | 37,39 | 13,16 | 11,11 | 20,21 | 17 | 14 | 13 | 30 | 23 | 9 | 11 | 14 | 14 | 10 | 11 | 13 | 28 | 20 | 28 | 15 | 17 | 10 | 25 | 37 | 12 | 12 | 13 | 14 | 19 | 17 | 15 | 16 | 20 | 22 | 10 | 8 | 12 | 2 | 2 | 2 |
| S595 | Korean | 35,40 | 15,16 | 10,17 | 20,21 | 17 | 12 | 15 | 30 | 23 | 10 | 13 | 13 | 14 | 13 | 12 | 14 | 25 | 18 | 30 | 15 | 17 | 10 | 23 | 41 | 13 | 11 | 12 | 14 | 17 | 17 | 16 | 15 | 21 | 21 | 13 | 8 | 11 | 1 | 2 | 2 |

**Table S4. The detailed variant information at various Y-STR loci.**

| Variant type | Y-STR loci | variants | number |
| --- | --- | --- | --- |
| micro-variants | DYF387S1 | 34.1,38 | 1 |
|  | DYF387S1 | 34.2,39 | 1 |
|  | DYF387S1 | 36,42.3 | 1 |
|  | DYF387S1 | 38.3,42.2 | 1 |
|  | DYF404S1 | 12.2,16 | 1 |
|  | DYF404S1 | 13.2,13.2 | 1 |
|  | DYF404S1 | 13.2,14 | 3 |
|  | DYF404S1 | 15.2,15.2 | 1 |
|  | DYF404S1 | 15.2,16.3 | 1 |
|  | DYS527 | 17.3,24.3 | 1 |
|  | DYS527 | 19.2,20.2 | 2 |
|  | DYS527 | 19.2,22.2 | 1 |
|  | DYS527 | 20.2,20.2 | 2 |
|  | DYS527 | 20.2,22.2 | 1 |
|  | DYS527 | 23.2,23.2 | 1 |
|  | DYS444 | 11.2 | 1 |
|  | DYS444 | 11.3 | 1 |
|  | DYS444 | 12.2 | 3 |
|  | DYS447 | 24.3 | 2 |
|  | DYS447 | 25.3 | 3 |
|  | DYS447 | 26.3 | 3 |
|  | DYS449 | 30.2 | 3 |
|  | DYS449 | 31.2 | 3 |
|  | DYS449 | 33.1 | 1 |
|  | DYS518 | 35.2 | 1 |
|  | DYS518 | 37.2 | 4 |
|  | DYS518 | 38.2 | 4 |
|  | DYS518 | 39.2 | 3 |
|  | DYS518 | 40.2 | 1 |
|  | DYS522 | 13.3 | 4 |
|  | DYS570 | 17.2 | 2 |
|  | DYS570 | 19.3 | 11 |
|  | DYS576 | 16.3 | 1 |
|  | DYS576 | 19.2 | 1 |
|  | DYS593 | 14.3 | 8 |
|  | DYS596 | 13.5 | 4 |
|  | DYS596 | 14.5 | 2 |
|  | DYS627 | 17.2 | 2 |
|  | DYS627 | 19.2 | 2 |
|  | DYS627 | 22.1 | 1 |
|  | DYS645 | 7.2 | 3 |
|  | DYS645 | 7.3 | 5 |
| Copy number variations | DYF387S1 | 34,35,36 | 1 |
|  | DYF387S1 | 35,36,37 | 3 |
|  | DYF387S1 | 35,39,40 | 1 |
|  | DYF387S1 | 35,39,41 | 1 |
|  | DYF387S1 | 36,38,41 | 1 |
|  | DYF404S1 | 13,14,15 | 2 |
|  | DYF404S1 | 13,14,16 | 1 |
|  | DYF404S1 | 13,15,16 | 1 |
|  | DYF404S1 | 14,15,16 | 2 |
|  | DYS385 | 11,12,13 | 1 |
|  | DYS385 | 12,17,19 | 1 |
|  | DYS385 | 13,14,18 | 1 |
|  | DYS385 | 13,16,20 | 1 |
|  | DYS385 | 13,17,19 | 1 |
|  | DYS527 | 19,20,21 | 1 |
|  | DYS527 | 19,22,23 | 1 |
|  | DYS527 | 20,23,24 | 1 |
|  | DYS527 | 21,22,23 | 1 |
|  | DYS527 | 22,23,24 | 1 |
|  | DYS19 | 16,17 | 1 |

**Table S5. The allele frequencies and GD values of single-copy loci in the 41-plex Y-STR Panel (n=595).**

| Allele | Allele | DYS19 | DYS388 | DYS389I | DYS389II | DYS390 | DYS391 | DYS392 | DYS393 | DYS437 | DYS438 | DYS439 | DYS444 | DYS447 | DYS448 | DYS449 | DYS456 | DYS458 | DYS460 | DYS481 | DYS518 | DYS522 | DYS533 | DYS549 | DYS557 | DYS570 | DYS576 | DYS593 | DYS596 | DYS627 | DYS635 | DYS643 | DYS645 |
| --- | --- | --- | --- | --- | --- | --- | --- | --- | --- | --- | --- | --- | --- | --- | --- | --- | --- | --- | --- | --- | --- | --- | --- | --- | --- | --- | --- | --- | --- | --- | --- | --- | --- |
| 6 | 6 |  |  |  |  |  |  |  |  |  |  |  |  |  |  |  |  |  |  |  |  |  |  |  |  |  |  |  |  |  |  |  | 0.0017 |
| 7 | 7 |  |  |  |  |  |  |  |  |  |  |  |  |  |  |  |  |  |  |  |  |  |  |  |  |  |  |  |  |  |  |  | 0.0050 |
| 7.2 | 7.2 |  |  |  |  |  |  |  |  |  |  |  |  |  |  |  |  |  |  |  |  |  |  |  |  |  |  |  |  |  |  |  | 0.0050 |
| 7.3 | 7.3 |  |  |  |  |  |  |  |  |  |  |  |  |  |  |  |  |  |  |  |  |  |  |  |  |  |  |  |  |  |  |  | 0.0084 |
| 8 | 8 |  |  |  |  |  | 0.0017 |  |  |  | 0.0017 |  |  |  |  |  |  |  | 0.0017 |  |  |  |  |  |  |  |  |  |  |  |  | 0.0252 | 0.9412 |
| 9 | 9 |  |  |  |  |  | 0.0471 |  |  |  | 0.0151 |  | 0.0034 |  |  |  |  |  | 0.2235 |  |  | 0.0101 | 0.0034 |  |  |  |  |  |  |  |  | 0.1143 | 0.0387 |
| 10 | 10 |  | 0.1429 | 0.0017 |  |  | 0.7681 | 0.0168 | 0.0017 |  | 0.6151 | 0.0521 | 0.0067 |  |  |  |  |  | 0.3479 |  |  | 0.0706 | 0.0723 | 0.0017 |  |  |  |  |  |  |  | 0.2538 |  |
| 11 | 11 |  |  | 0.0050 |  |  | 0.1832 | 0.1630 | 0.0050 |  | 0.1866 | 0.3328 | 0.0605 |  |  |  |  |  | 0.3798 |  |  | 0.4235 | 0.5899 | 0.0773 | 0.0017 |  |  |  |  |  |  | 0.3546 |  |
| 11.2 | 11.2 |  |  |  |  |  |  |  |  |  |  |  | 0.0017 |  |  |  |  |  |  |  |  |  |  |  |  |  |  |  |  |  |  |  |  |
| 11.3 | 11.3 |  |  |  |  |  |  |  |  |  |  |  | 0.0017 |  |  |  |  |  |  |  |  |  |  |  |  |  |  |  |  |  |  |  |  |
| 12 | 12 | 0.0034 | 0.6639 | 0.4403 |  |  |  | 0.0941 | 0.4756 |  | 0.0319 | 0.4857 | 0.3613 |  |  |  | 0.0084 |  | 0.0420 |  |  | 0.3866 | 0.3042 | 0.5529 | 0.0118 |  |  |  |  |  |  | 0.1647 |  |
| 12.2 | 12.2 |  |  |  |  |  |  |  |  |  |  |  | 0.0050 |  |  |  |  |  |  |  |  |  |  |  |  |  |  |  |  |  |  |  |  |
| 13 | 13 | 0.0336 | 0.1714 | 0.2723 |  |  |  | 0.4353 | 0.3109 | 0.0067 | 0.1462 | 0.1025 | 0.3412 |  |  |  | 0.0218 | 0.0017 | 0.0050 |  |  | 0.0924 | 0.0252 | 0.2924 | 0.0336 | 0.0017 |  |  | 0.0034 |  |  | 0.0773 |  |
| 13.3 | 13.3 |  |  |  |  |  |  |  |  |  |  |  |  |  |  |  |  |  |  |  |  | 0.0067 |  |  |  |  |  |  |  |  |  |  |  |
| 13.5 | 13.5 |  |  |  |  |  |  |  |  |  |  |  |  |  |  |  |  |  |  |  |  |  |  |  |  |  |  |  | 0.0067 |  |  |  |  |
| 14 | 14 | 0.1832 | 0.0151 | 0.2689 |  |  |  | 0.2403 | 0.1664 | 0.6975 | 0.0034 | 0.0252 | 0.1983 |  |  |  | 0.1630 | 0.0067 |  |  |  | 0.0101 | 0.0050 | 0.0639 | 0.4134 | 0.0101 | 0.0050 |  | 0.2975 | 0.0017 |  | 0.0084 |  |
| 14.1 | 14.1 |  |  |  |  |  |  |  |  |  |  |  |  |  |  |  |  |  |  |  |  |  |  |  | 0.0034 |  |  |  |  |  |  |  |  |
| 14.3 | 14.3 |  |  |  |  |  |  |  |  |  |  |  |  |  |  |  |  |  |  |  |  |  |  |  |  |  |  | 0.0134 |  |  |  |  |  |
| 14.5 | 14.5 |  |  |  |  |  |  |  |  |  |  |  |  |  |  |  |  |  |  |  |  |  |  |  |  |  |  |  | 0.0034 |  |  |  |  |
| 15 | 15 | 0.4050 | 0.0050 | 0.0118 |  |  |  | 0.0437 | 0.0370 | 0.2824 |  | 0.0017 | 0.0185 |  |  |  | 0.6303 | 0.0857 |  |  |  |  |  | 0.0118 | 0.1916 | 0.0303 | 0.0151 | 0.2000 | 0.5849 | 0.0017 |  | 0.0017 |  |
| 16 | 16 | 0.2487 | 0.0017 |  |  |  |  | 0.0050 | 0.0034 | 0.0134 |  |  | 0.0017 |  |  |  | 0.1429 | 0.1916 |  |  |  |  |  |  | 0.1462 | 0.1462 | 0.0723 | 0.5664 | 0.0924 | 0.0017 |  |  |  |
| 16.2 | 16.2 |  |  |  |  |  |  |  |  |  |  |  |  |  |  |  |  |  |  |  |  |  |  |  | 0.0017 |  |  |  |  |  |  |  |  |
| 16.3 | 16.3 |  |  |  |  |  |  |  |  |  |  |  |  |  |  |  |  |  |  |  |  |  |  |  |  |  | 0.0017 |  |  |  |  |  |  |
| 16,17 | 16,17 | 0.0017 |  |  |  |  |  |  |  |  |  |  |  |  |  |  |  |  |  |  |  |  |  |  |  |  |  |  |  |  |  |  |  |
| 17 | 17 | 0.1227 |  |  |  |  |  | 0.0017 |  |  |  |  |  | 0.0017 | 0.0168 |  | 0.0252 | 0.2958 |  |  |  |  |  |  | 0.1227 | 0.1782 | 0.2487 | 0.2118 | 0.0101 | 0.0202 |  |  |  |
| 17.2 | 17.2 |  |  |  |  |  |  |  |  |  |  |  |  |  |  |  |  |  |  |  |  |  |  |  |  | 0.0034 |  |  |  | 0.0034 |  |  |  |
| 18 | 18 | 0.0017 |  |  |  |  |  |  |  |  |  |  |  |  | 0.2420 |  | 0.0084 | 0.2504 |  |  |  |  |  |  | 0.0319 | 0.2874 | 0.3261 | 0.0084 | 0.0017 | 0.0689 | 0.0017 |  |  |
| 19 | 19 |  |  |  |  |  |  |  |  |  |  |  |  |  | 0.3193 |  |  | 0.1193 |  | 0.0067 |  |  |  |  | 0.0286 | 0.1899 | 0.2034 |  |  | 0.0941 | 0.0471 |  |  |
| 19.1 | 19.1 |  |  |  |  |  |  |  |  |  |  |  |  |  |  |  |  |  |  |  |  |  |  |  | 0.0017 |  |  |  |  |  |  |  |  |
| 19.2 | 19.2 |  |  |  |  |  |  |  |  |  |  |  |  |  |  |  |  |  |  |  |  |  |  |  |  |  | 0.0017 |  |  | 0.0034 |  |  |  |
| 19.3 | 19.3 |  |  |  |  |  |  |  |  |  |  |  |  |  |  |  |  |  |  |  |  |  |  |  |  | 0.0185 |  |  |  |  |  |  |  |
| 20 | 20 |  |  |  |  |  |  |  |  |  |  |  |  |  | 0.2655 |  |  | 0.0336 |  | 0.0134 |  |  |  |  | 0.0101 | 0.0958 | 0.0924 |  |  | 0.2202 | 0.2639 |  |  |
| 20.1 | 20.1 |  |  |  |  |  |  |  |  |  |  |  |  |  |  |  |  |  |  |  |  |  |  |  | 0.0017 |  |  |  |  |  |  |  |  |
| 21 | 21 |  |  |  |  | 0.0118 |  |  |  |  |  |  |  | 0.0017 | 0.1227 |  |  | 0.0101 |  | 0.0739 |  |  |  |  |  | 0.0319 | 0.0269 |  |  | 0.2353 | 0.3899 |  |  |
| 22 | 22 |  |  |  |  | 0.0908 |  |  |  |  |  |  |  | 0.0101 | 0.0303 |  |  | 0.0050 |  | 0.1076 |  |  |  |  |  | 0.0050 | 0.0050 |  |  | 0.1798 | 0.1950 |  |  |
| 22.1 | 22.1 |  |  |  |  |  |  |  |  |  |  |  |  |  |  |  |  |  |  |  |  |  |  |  |  |  |  |  |  | 0.0017 |  |  |  |
| 23 | 23 |  |  |  |  | 0.4286 |  |  |  |  |  |  |  | 0.1866 | 0.0034 |  |  |  |  | 0.3261 |  |  |  |  |  | 0.0017 | 0.0017 |  |  | 0.1059 | 0.0706 |  |  |
| 24 | 24 |  |  |  | 0.0017 | 0.3076 |  |  |  |  |  |  |  | 0.2655 |  | 0.0017 |  |  |  | 0.2538 |  |  |  |  |  |  |  |  |  | 0.0538 | 0.0286 |  |  |
| 24.3 | 24.3 |  |  |  |  |  |  |  |  |  |  |  |  | 0.0034 |  |  |  |  |  |  |  |  |  |  |  |  |  |  |  |  |  |  |  |
| 25 | 25 |  |  |  |  | 0.1462 |  |  |  |  |  |  |  | 0.2824 |  | 0.0034 |  |  |  | 0.1092 |  |  |  |  |  |  |  |  |  | 0.0084 | 0.0017 |  |  |
| 25.3 | 25.3 |  |  |  |  |  |  |  |  |  |  |  |  | 0.0050 |  |  |  |  |  |  |  |  |  |  |  |  |  |  |  |  |  |  |  |
| 26 | 26 |  |  |  | 0.0017 | 0.0101 |  |  |  |  |  |  |  | 0.1092 |  | 0.0118 |  |  |  | 0.0571 |  |  |  |  |  |  |  |  |  |  | 0.0017 |  |  |
| 26.3 | 26.3 |  |  |  |  |  |  |  |  |  |  |  |  | 0.0050 |  |  |  |  |  |  |  |  |  |  |  |  |  |  |  |  |  |  |  |
| 27 | 27 |  |  |  | 0.0790 | 0.0050 |  |  |  |  |  |  |  | 0.0840 |  | 0.0605 |  |  |  | 0.0319 |  |  |  |  |  |  |  |  |  |  |  |  |  |
| 28 | 28 |  |  |  | 0.2975 |  |  |  |  |  |  |  |  | 0.0286 |  | 0.0672 |  |  |  | 0.0151 |  |  |  |  |  |  |  |  |  |  |  |  |  |
| 29 | 29 |  |  |  | 0.3227 |  |  |  |  |  |  |  |  | 0.0134 |  | 0.1210 |  |  |  | 0.0050 |  |  |  |  |  |  |  |  |  |  |  |  |  |
| 30 | 30 |  |  |  | 0.2387 |  |  |  |  |  |  |  |  | 0.0017 |  | 0.2000 |  |  |  |  |  |  |  |  |  |  |  |  |  |  |  |  |  |
| 30.2 | 30.2 |  |  |  |  |  |  |  |  |  |  |  |  |  |  | 0.0050 |  |  |  |  |  |  |  |  |  |  |  |  |  |  |  |  |  |
| 31 | 31 |  |  |  | 0.0471 |  |  |  |  |  |  |  |  |  |  | 0.1563 |  |  |  |  |  |  |  |  |  |  |  |  |  |  |  |  |  |
| 31.2 | 31.2 |  |  |  |  |  |  |  |  |  |  |  |  |  |  | 0.0050 |  |  |  |  |  |  |  |  |  |  |  |  |  |  |  |  |  |
| 32 | 32 |  |  |  | 0.0101 |  |  |  |  |  |  |  |  | 0.0017 |  | 0.1899 |  |  |  |  |  |  |  |  |  |  |  |  |  |  |  |  |  |
| 33 | 33 |  |  |  | 0.0017 |  |  |  |  |  |  |  |  |  |  | 0.0790 |  |  |  |  | 0.0067 |  |  |  |  |  |  |  |  |  |  |  |  |
| 33.1 | 33.1 |  |  |  |  |  |  |  |  |  |  |  |  |  |  | 0.0017 |  |  |  |  |  |  |  |  |  |  |  |  |  |  |  |  |  |
| 34 | 34 |  |  |  |  |  |  |  |  |  |  |  |  |  |  | 0.0622 |  |  |  |  | 0.0185 |  |  |  |  |  |  |  |  |  |  |  |  |
| 35 | 35 |  |  |  |  |  |  |  |  |  |  |  |  |  |  | 0.0252 |  |  |  |  | 0.0874 |  |  |  |  |  |  |  |  |  |  |  |  |
| 35.2 | 35.2 |  |  |  |  |  |  |  |  |  |  |  |  |  |  |  |  |  |  |  | 0.0017 |  |  |  |  |  |  |  |  |  |  |  |  |
| 36 | 36 |  |  |  |  |  |  |  |  |  |  |  |  |  |  | 0.0050 |  |  |  |  | 0.1378 |  |  |  |  |  |  |  |  |  |  |  |  |
| 37 | 37 |  |  |  |  |  |  |  |  |  |  |  |  |  |  | 0.0050 |  |  |  |  | 0.1765 |  |  |  |  |  |  |  |  |  |  |  |  |
| 37.2 | 37.2 |  |  |  |  |  |  |  |  |  |  |  |  |  |  |  |  |  |  |  | 0.0067 |  |  |  |  |  |  |  |  |  |  |  |  |
| 38 | 38 |  |  |  |  |  |  |  |  |  |  |  |  |  |  |  |  |  |  |  | 0.1849 |  |  |  |  |  |  |  |  |  |  |  |  |
| 38.2 | 38.2 |  |  |  |  |  |  |  |  |  |  |  |  |  |  |  |  |  |  |  | 0.0067 |  |  |  |  |  |  |  |  |  |  |  |  |
| 39 | 39 |  |  |  |  |  |  |  |  |  |  |  |  |  |  |  |  |  |  |  | 0.1529 |  |  |  |  |  |  |  |  |  |  |  |  |
| 39.2 | 39.2 |  |  |  |  |  |  |  |  |  |  |  |  |  |  |  |  |  |  |  | 0.0050 |  |  |  |  |  |  |  |  |  |  |  |  |
| 40 | 40 |  |  |  |  |  |  |  |  |  |  |  |  |  |  |  |  |  |  |  | 0.0992 |  |  |  |  |  |  |  |  |  |  |  |  |
| 40.2 | 40.2 |  |  |  |  |  |  |  |  |  |  |  |  |  |  |  |  |  |  |  | 0.0017 |  |  |  |  |  |  |  |  |  |  |  |  |
| 41 | 41 |  |  |  |  |  |  |  |  |  |  |  |  |  |  |  |  |  |  |  | 0.0672 |  |  |  |  |  |  |  |  |  |  |  |  |
| 42 | 42 |  |  |  |  |  |  |  |  |  |  |  |  |  |  |  |  |  |  |  | 0.0319 |  |  |  |  |  |  |  |  |  |  |  |  |
| 43 | 43 |  |  |  |  |  |  |  |  |  |  |  |  |  |  |  |  |  |  |  | 0.0101 |  |  |  |  |  |  |  |  |  |  |  |  |
| 44 | 44 |  |  |  |  |  |  |  |  |  |  |  |  |  |  |  |  |  |  |  | 0.0050 |  |  |  |  |  |  |  |  |  |  |  |  |
| GD | GD | 0.7255 | 0.5101 | 0.6606 | 0.7431 | 0.6930 | 0.3749 | 0.7163 | 0.6491 | 0.4343 | 0.5651 | 0.6405 | 0.7108 | 0.7961 | 0.7536 | 0.8671 | 0.5555 | 0.7915 | 0.6841 | 0.7969 | 0.8701 | 0.6585 | 0.5545 | 0.5995 | 0.7539 | 0.8180 | 0.7770 | 0.5951 | 0.5616 | 0.8370 | 0.7335 | 0.7642 | 0.1128 |

**Table S6. The haplotype frequency and haplotype diversity of multi-copy loci in the 41-plex Y-STR Panel (n=595).**

| DYF387S1 | | DYF404S1a/b1 | | DYS385a/b | | DYS527a/b | |
| --- | --- | --- | --- | --- | --- | --- | --- |
| Haplotype | Frequence | Haplotype | Frequence | Haplotype | Frequence | Haplotype | Frequence |
| 30,39 | 0.0017 | 9,16 | 0.0034 | 9,11 | 0.0017 | 16,22 | 0.0017 |
| 34,34 | 0.0017 | 9,17 | 0.0084 | 9,16 | 0.0017 | 17.3,24.3 | 0.0017 |
| 34,35,36 | 0.0017 | 11,11 | 0.0084 | 9,18 | 0.0017 | 18,20 | 0.0034 |
| 34,36 | 0.0067 | 11,12 | 0.0017 | 9,19 | 0.0067 | 18,21 | 0.0034 |
| 34,37 | 0.0050 | 11,13 | 0.0017 | 9,21 | 0.0017 | 18,22 | 0.0017 |
| 34,38 | 0.0168 | 11,14 | 0.0067 | 10,10 | 0.0050 | 18,23 | 0.0017 |
| 34,39 | 0.0118 | 12,12 | 0.0050 | 10,12 | 0.0017 | 18,24 | 0.0017 |
| 34,40 | 0.0134 | 12,13 | 0.0454 | 10,16 | 0.0017 | 19,19 | 0.0067 |
| 34,41 | 0.0017 | 12,14 | 0.0202 | 10,17 | 0.0336 | 19,20 | 0.0689 |
| 34.1,38 | 0.0017 | 12,15 | 0.0134 | 10,18 | 0.0403 | 19,20,21 | 0.0017 |
| 34.2,39 | 0.0017 | 12,16 | 0.0067 | 10,19 | 0.0420 | 19,21 | 0.0185 |
| 35,35 | 0.0151 | 12,17 | 0.0034 | 10,20 | 0.0235 | 19,22 | 0.0084 |
| 35,36 | 0.0084 | 12.2,16 | 0.0017 | 10,21 | 0.0084 | 19,22,23 | 0.0017 |
| 35,36,37 | 0.0050 | 13,13 | 0.1126 | 11,11 | 0.0252 | 19,23 | 0.0084 |
| 35,37 | 0.0387 | 13,14 | 0.0924 | 11,12 | 0.0286 | 19,24 | 0.0050 |
| 35,38 | 0.0521 | 13,14,15 | 0.0034 | 11,12,13 | 0.0017 | 19,25 | 0.0034 |
| 35,39 | 0.0975 | 13,14,16 | 0.0017 | 11,13 | 0.0118 | 19.2,20.2 | 0.0034 |
| 35,39,40 | 0.0017 | 13,15 | 0.0353 | 11,14 | 0.0101 | 19.2,22.2 | 0.0017 |
| 35,39,41 | 0.0017 | 13,15,16 | 0.0017 | 11,15 | 0.0017 | 20,20 | 0.0387 |
| 35,40 | 0.0571 | 13,16 | 0.0218 | 11,16 | 0.0118 | 20,21 | 0.1059 |
| 35,41 | 0.0118 | 13,17 | 0.0050 | 11,17 | 0.0303 | 20,22 | 0.0370 |
| 35,42 | 0.0034 | 13.2,13.2 | 0.0017 | 11,18 | 0.0353 | 20,23 | 0.0437 |
| 36,36 | 0.0336 | 13.2,14 | 0.0050 | 11,19 | 0.0168 | 20,23,24 | 0.0017 |
| 36,37 | 0.0319 | 14,14 | 0.0908 | 11,20 | 0.0084 | 20,24 | 0.0336 |
| 36,38 | 0.0773 | 14,15 | 0.1076 | 11,21 | 0.0017 | 20,25 | 0.0151 |
| 36,38,41 | 0.0017 | 14,15,16 | 0.0034 | 12,12 | 0.0202 | 20,26 | 0.0017 |
| 36,39 | 0.0723 | 14,16 | 0.0807 | 12,13 | 0.0235 | 20,27 | 0.0017 |
| 36,40 | 0.0336 | 14,17 | 0.0319 | 12,14 | 0.0034 | 20.2,20.2 | 0.0034 |
| 36,41 | 0.0151 | 14,18 | 0.0034 | 12,15 | 0.0134 | 20.2,22.2 | 0.0017 |
| 36,42.3 | 0.0017 | 14,19 | 0.0017 | 12,16 | 0.0303 | 21,21 | 0.0739 |
| 37,37 | 0.0739 | 15,15 | 0.0840 | 12,17 | 0.0471 | 21,22 | 0.0891 |
| 37,38 | 0.0739 | 15,16 | 0.0891 | 12,17,19 | 0.0017 | 21,22,23 | 0.0017 |
| 37,39 | 0.0538 | 15,17 | 0.0168 | 12,18 | 0.0639 | 21,23 | 0.0723 |
| 37,40 | 0.0151 | 15,18 | 0.0067 | 12,19 | 0.0437 | 21,24 | 0.0370 |
| 37,41 | 0.0067 | 15.2,15.2 | 0.0017 | 12,20 | 0.0151 | 21,25 | 0.0050 |
| 38,38 | 0.0538 | 15.2,16.3 | 0.0017 | 12,21 | 0.0084 | 21,26 | 0.0034 |
| 38,39 | 0.0286 | 16,16 | 0.0487 | 13,13 | 0.0252 | 22,22 | 0.0655 |
| 38,40 | 0.0168 | 16,17 | 0.0151 | 13,14 | 0.0067 | 22,23 | 0.0756 |
| 38,41 | 0.0034 | 17,17 | 0.0084 | 13,14,18 | 0.0017 | 22,23,24 | 0.0017 |
| 38.3,42.2 | 0.0017 | 18,18 | 0.0017 | 13,15 | 0.0050 | 22,24 | 0.0336 |
| 39,39 | 0.0185 |  |  | 13,16 | 0.0084 | 22,25 | 0.0034 |
| 39,40 | 0.0084 |  |  | 13,16,20 | 0.0017 | 23,23 | 0.0588 |
| 39,41 | 0.0050 |  |  | 13,17 | 0.0168 | 23,24 | 0.0218 |
| 40,40 | 0.0118 |  |  | 13,17,19 | 0.0017 | 23,25 | 0.0118 |
| 40,41 | 0.0034 |  |  | 13,18 | 0.0336 | 23.2,23.2 | 0.0017 |
| 42,42 | 0.0017 |  |  | 13,19 | 0.0555 | 24,24 | 0.0101 |
|  |  |  |  | 13,20 | 0.0202 | 24,25 | 0.0017 |
|  |  |  |  | 13,21 | 0.0118 | 25,25 | 0.0017 |
|  |  |  |  | 13,22 | 0.0017 | 25,26 | 0.0017 |
|  |  |  |  | 13,23 | 0.0017 | 30,30 | 0.0017 |
|  |  |  |  | 14,14 | 0.0034 |  |  |
|  |  |  |  | 14,15 | 0.0017 |  |  |
|  |  |  |  | 14,16 | 0.0034 |  |  |
|  |  |  |  | 14,17 | 0.0252 |  |  |
|  |  |  |  | 14,18 | 0.0303 |  |  |
|  |  |  |  | 14,19 | 0.0151 |  |  |
|  |  |  |  | 14,20 | 0.0034 |  |  |
|  |  |  |  | 14,21 | 0.0084 |  |  |
|  |  |  |  | 14,22 | 0.0050 |  |  |
|  |  |  |  | 14,23 | 0.0017 |  |  |
|  |  |  |  | 14,25 | 0.0017 |  |  |
|  |  |  |  | 15,15 | 0.0034 |  |  |
|  |  |  |  | 15,16 | 0.0034 |  |  |
|  |  |  |  | 15,17 | 0.0084 |  |  |
|  |  |  |  | 15,19 | 0.0101 |  |  |
|  |  |  |  | 15,20 | 0.0101 |  |  |
|  |  |  |  | 15,21 | 0.0101 |  |  |
|  |  |  |  | 15,22 | 0.0067 |  |  |
|  |  |  |  | 15,23 | 0.0050 |  |  |
|  |  |  |  | 16,16 | 0.0034 |  |  |
|  |  |  |  | 16,17 | 0.0034 |  |  |
|  |  |  |  | 16,19 | 0.0017 |  |  |
|  |  |  |  | 16,20 | 0.0034 |  |  |
|  |  |  |  | 16,21 | 0.0034 |  |  |
|  |  |  |  | 17,17 | 0.0050 |  |  |
|  |  |  |  | 17,20 | 0.0017 |  |  |
|  |  |  |  | 19,19 | 0.0034 |  |  |
|  |  |  |  | 21,21 | 0.0017 |  |  |
|  |  |  |  | 21,22 | 0.0017 |  |  |
| HD | 0.9500 |  | 0.9302 |  | 0.9732 |  | 0.9435 |
